# Influence of Steric Effect on the Pseudo-Multicomponent Synthesis of *N*-Aroylmethyl-4-Arylimidazoles

**DOI:** 10.3390/molecules27041165

**Published:** 2022-02-09

**Authors:** Nerith Rocio Elejalde-Cadena, Mayra García-Olave, David Figueroa, Pietro Vidossich, Gian Pietro Miscione, Jaime Portilla

**Affiliations:** 1 Bioorganic Compounds Research Group, Department of Chemistry, Universidad de los Andes, Carrera 1 No. 18A-10, Bogotá 111711, Colombia; nr.elejalde10@uniandes.edu.co (N.R.E.-C.); m.garciao@uniandes.edu.co (M.G.-O.); 2 COBO-Computational Bio-Organic Chemistry Bogotá, Department of Chemistry, Universidad de los Andes, Cra 1 No. 18A-12, Bogotá 111711, Colombia; dr.figueroa10@uniandes.edu.co (D.F.); gp.miscione57@uniandes.edu.co (G.P.M.); 3 Laboratory of Molecular Modeling and Drug Discovery, Istituto Italiano di Tecnologia, Via Morego 30, 16163 Genova, Italy; Pietro.vidossich@iit.it

**Keywords:** amidines, *N*-aroylmethylimidazoles, DFT calculations, pseudo-MCRs, steric effect

## Abstract

A pseudo-three-component synthesis of *N*-aroylmethylimidazoles 3 with three new C–N bonds formed regioselectively under microwave conditions was developed. Products were obtained by reacting two equivalents of aroylmethyl bromide (ArCOCH_2_Br, **1**) with the appropriate amidine salt (RCN_2_H_3_.HX, **2**) and with K_2_CO_3_ as a base in acetonitrile. The bicomponent reaction also occurred, giving the expected 4(5)-aryl-1*H*-imidazoles **4**. Notably, the ratio of products **3** and **4** is governed by steric factors of the amidine **2** (i.e., R = H, CH_3_, Ph). Therefore, a computational study was carried out to understand the reaction course regarding product ratio (**3**/**4**), regioselectivity, and the steric effects of the amidine substituent group.

## 1. Introduction

**Figure 1:**
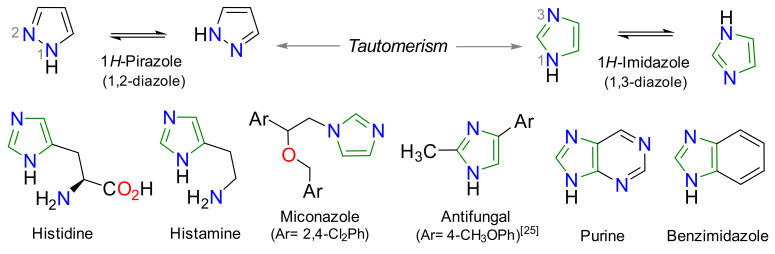
Diazole structure and examples of imidazoles (green rings) of general interest.

Diazoles are five-membered *N*-heterocyclic compounds having two nitrogen atoms, one pyrrole-like and the other pyridine-like [1,2]. Compounds bearing the diazole ring are of particular interest to different fields of chemistry, industry, and medicine because of their relevance in the needs of society [3,4,5] (Figure 1). Consequently, various methods for the synthesis and functionalization of diazoles have been established [1,2,3,4,5,6,7].

Despite the impact of diazoles, there are scarce examples of naturally occurring compounds with the pyrazolic ring (1,2-diazole) [1]; however, the imidazole ring (1,3-diazole) is recurrent in diverse natural compounds (e.g., vitamin B-12, purines, histidine, histamine, etc.) [2,7]. The imidazole core is one of the most common among nitrogen-containing pharmaceuticals [8] and has been found to exhibit anti-inflammatory [9], antifungal [10], and antitumor activity [11], among other effects (Figure 1). Moreover, it presents applications in catalysis [12], materials science [13], agrochemicals [14], and chemosensors [15]. Thus, the methods for imidazole synthesis are extensive and can be classified in three general ways: (i) cyclocondensation reactions (the most usual), (ii) a scaffold approach, and (iii) multicomponent reactions (MCRs) [2,7,16,17,18,19,20,21,22]. Several methods have been perfected using microwave-assisted organic synthesis (MAOS) as a powerful tool for various chemical transformations with superior results compared to conventional methods [20,23,24,25,26].

**Scheme 1:**
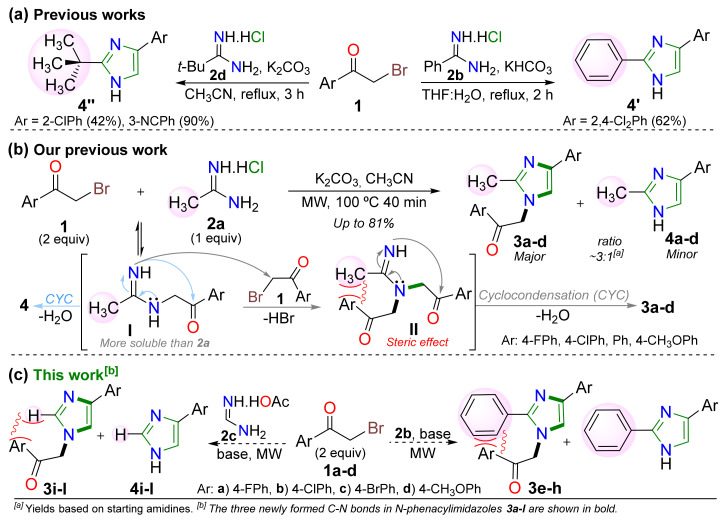
Synthesis of imidazoles starting from aroylmethyl bromides **1** and amidines (**a**) **2b**/**2d**, (**b**) **2a**, and (**c**) **2b**/**2c**.

It is important to note that few studies have been reported to obtain imidazoles using aroylmethyl bromides 1 and amidines **2** [21,22] (Scheme 1a), specifically *N*-substituted derivatives that we even obtained by a pseudo-multicomponent reaction (pseudo-MCR) [24,26] (Scheme 1b). Likewise, there are few reports on imidazole derivatives synthesis based on pseudo-MCRs and MAOS. In a pseudo-MCR two or more components are identical, which could be a limitation regarding the scope and functional flexibility. However, these reactions have the advantage of being efficient in obtaining products with molecular complexity. In particular, we developed a pseudo-three-component synthesis of *N*-aroylmethylimidazoles **3a–d** under microwave (MW) conditions in good yields by reacting two equivalents of 1 with acetamidine hydrochloride (**2a**) [24,25,26]. This reaction also allowed us to obtain the expected 1*H*-imidazoles **4** as minor products (Scheme 1b). Possibly, the better solubility in organic solvents and the major steric effect in the cyclization step from amidines **2b** [21] and **2d** [22] (Scheme 1a) vs. **2a** [24,25,26] (Scheme 1b) direct the reaction course.

From these interesting findings and in search of testing our hypothesis of the steric effect, we decided to carry out the reaction using benzamidine hydrochloride (**2b**) and formamidinium acetate (**2c**) to ensure a good size difference of the substituent at position 2 of the imidazole ring (Scheme 1c). In addition, we performed a detailed computational study to understand the reaction course regarding the obtained results in the regioselective synthesis of *N*-aroylmethyl-4-arylimidazoles **3a–l** and the expected 4(5)-aryl-1*H*-imidazoles **4**. In the same way, this theoretical study would validate the proposed mechanism in our previous work. Ultimately, the new compounds are imidazole antifungal analogs [24,25,26,27], thus possessing significant biological potential. For example, by simple carbonyl group reduction, the aroylmethyl group of **3e–l** can be converted to the privileged 2-aryl-2-hydroxyethyl moiety present in various antifungal agents [24,26,27].

## 2. Results and Discussion

**Scheme 2:**
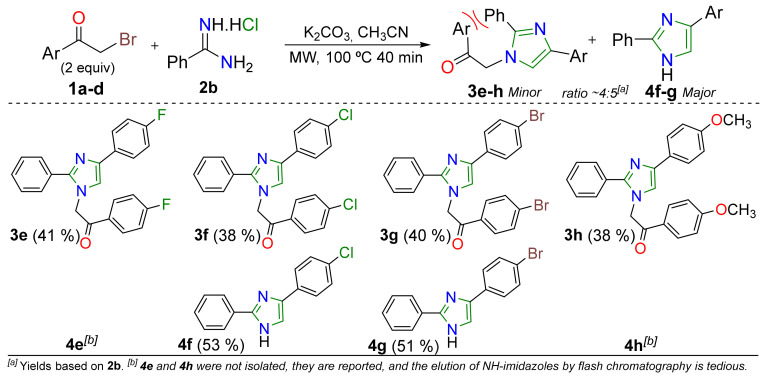
Synthesis of *N*-aroylmethyl-4-arylimidazoles **3e–h** and 4(5)-aryl-1*H*-imidazoles **4f–g**.

Considering our previous work on the MW-assisted synthesis of *N*-aroylmethyl-2-methylimidazoles **3a–d** by a pseudo-MCR [24,25,26], we planned to study a similar protocol using the amidines **2b–c** instead of **2a**. This investigation was carried out to determine the steric effect influence of amidine salts **2a–c** on the *N*-aroylmethyl-4-arylimidazoles **3a–l** synthesis. The study began with the reaction between 2 equivalents of the respective α-bromoketone 1a–d with one equivalent of benzamidine **2b**. Gratifyingly, the desired *N*-aroylmethyl-4-aryl-2-phenylimidazoles **3e–h** together with 4(5)-aryl-2-phenyl-1*H*-imidazoles **4f–g** were obtained in good yields in an estimated 4:5 ratio (Scheme 2). Products were purified by flash chromatography on silica gel; the first eluted fraction (eluent: DCM) had the new compounds **3e–h**, while the second fraction (very slow, with a gradual MeOH increase up to DCM/MeOH 20:1 *v*/*v*) had the 1*H*-imidazoles **4a–g** (major product).

Results obtained for the 2-phenylimidazoles **3e–h** and **4f–g** synthesis agree with the expected steric and solubility effects since the 2-phenyl-1*H*-imidazoles are favored with **2b** while *N*-substituted products are preferred using acetamidine **2a**. Possibly, the better solubility in acetonitrile of **2b** with regard to **2a** (seven vs. two carbon atoms) favors both its interaction with the substrate **1a–b** and the equimolar reaction, which would not happen with **2a**. Moreover, the steric effect in the cyclization step from amidines **2a** or **2b** (CH_3_ vs. Ph [21], see Scheme 1) also favors 1*H*-imidazoles **4f–g** over products **3e–h**. Compounds **4e–h** have been reported in the literature through other synthetic ways; thus, we considered it sufficient to isolate only products **4f** and **4g** to extrapolate and generalize the proportions of products. This was developed based on our previous work, where we managed to isolate 2-methyl-*NH*-imidazoles in approximate yields (around 20%). In general, the elution of *NH*-imidazoles by column chromatography is tedious because they tend to become significantly adsorbed on the silica gel; the process may take an entire day. However, the pseudo-MCR is more important because it allows access to novel *N*-substituted imidazoles.

**Scheme 3:**
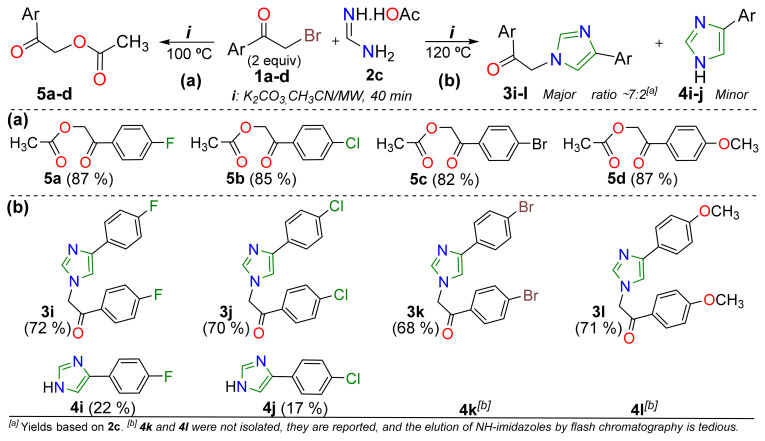
Synthesis of (**a**) aroylmethyl acetates **5a–d** and (**b**) imidazoles **3i–l** and **4i–j**.

Once the 2-phenylimidazoles **3e–h** and **4f–g** were obtained, we carried out the reaction between two equivalents of 1a–d with one equivalent of formamidinium acetate (**2c**) under similar conditions. Although four products were formed, they did not correspond to the desired imidazoles but the aroylmethyl acetates **5a–d** (Scheme 3a). These products were obtained by the reaction of the acetate group in **2c** with **1a–d** under these conditions. Thus, we increased the reaction temperature to 120 °C to favor the imidazoles’ formation, possibly from **5a–d**, with the formamidine in the reaction medium. Gratifyingly, the *N*-aroylmethylimidazoles **3i–l** and 1*H*-imidazoles **4i–j** were obtained in high yields and an estimated 7:2 ratio under these conditions (Scheme 3b). These results also agree with the steric effects; indeed, the *N*-substituted products are obtained in a higher proportion than when amidines **2a–b** were used (**3a–l**/**4a–l**, H (3.5:1) vs. CH_3_ (3:1) and Ph (1:1.25), Scheme 1b, Scheme 2, and Scheme 3b). Structures of all products obtained were characterized by ^1^H and ^13^C NMR studies and HRMS analysis (see the Experimental Section and Supporting Information for details).

**Scheme 4:**
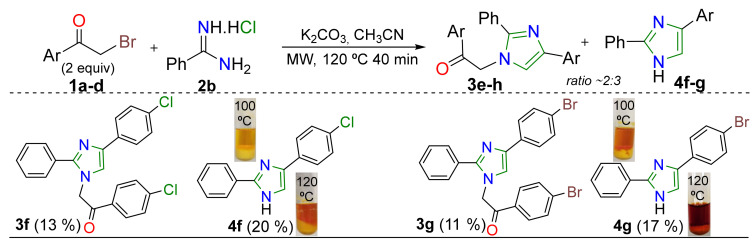
Microwave-assisted synthesis at 120 °C of 2-phenylimidazoles **3f**, **4f**, **3g**, and **4g**. Photographs of the reaction mixtures at 100 °C (top) and 120 °C (bottom) are shown.

Regarding the imidazoles’ synthesis **3** and **4** via the reaction of aroylmethyl bromide 1 with amidines **2**, a plausible and general mechanism in Scheme 4 is depicted [24]. The *N*-substituted imidazoles **3a–l** are formed by a regioselective pseudo-MCR of three steps (di-*N*-alkylation **i**/**iib**, cyclocondensation **iii**), while 1*H*-imidazoles **4** are obtained through a two-step process (*N*-alkylation **i**, cyclocondensation **iia**). It starts with a nucleophilic attack of the amidine **2** on substrate **1** (or the ketoester **5** in situ generated) to form the intermediate A, which can undergo a cyclocondensation reaction via the alcohol 4* to afford products **4** or a second *N*-alkylation with **1** (or **5**) giving the cyclization intermediate B. This intermediate can then involve a cyclocondensation by attacking its nucleophilic nitrogen atom on a carbonyl group, with the later loss of a water molecule from 4*. It is important to emphasize that due to steric factors, the *N*-alkylation of A (intermolecular) or cyclocondensation of B (intramolecular) may be disadvantaged (Scheme 4).

Despite the promising results in synthesizing imidazoles **3a–l** and **4**, we carried out some examples starting from benzamidine hydrochloride (**2b**) at 120 °C, as in the reaction with formamidine acetate (**2c**). In those reactions, microwave irradiation of reagents (substrates **3b**/**4**-Cl and **3c**/**4**-Br were tested) led to the formation of the expected imidazoles in poor yields, and the **3**/**4** product ratio turned out to be somewhat lower (Scheme 4 vs. Scheme 2). Possibly, the higher temperature promotes the reaction mixture deterioration; Scheme 4 shows photographs of the reaction mixtures at 100 °C and 120 °C, the one at 120 °C being a little darker due to its possible decomposition. These results were not observed using **2c** because we believe the reaction occurs via ketoesters **5a–d** as 1,2-bis-electrophilic substrates; indeed, the obtention of these ketoesters led us to develop the reaction at 120 °C.

**Scheme 5:**
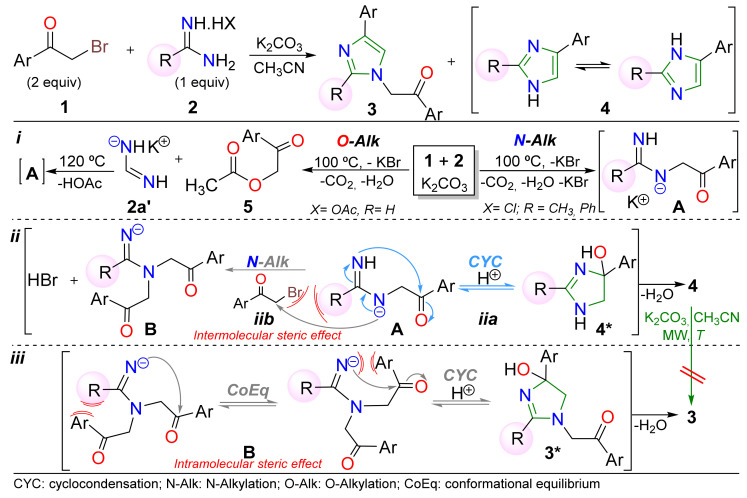
Plausible mechanism for the formation of *N*-aroylmethylimidazoles **3** and 1*H*-imidazoles **4** (**i,ii,iii**).

On only considering our experimental and literature research, it is possible to conclude that this reaction type is governed by the steric effect of the R group attached to the starting amidine. Studies have established that the smaller the R group in **2a–d**, the more favored is the *N*-alkylation reaction of A (i.e., H > CH_3_ > Ph > *t*-Bu) regarding the intramolecular cyclocondensation reaction; indeed, our new findings correspond with results reported in the literature [21,22,24,25,26]. Remarkably, these reactions occur with high regioselectivity, and steric factors govern their course regarding yields and product ratio (imidazoles **3** vs. **4**) (Scheme 5). It is important to mention that the formation of **3** from *NH*-imidazoles **4** was discarded because the *N*-alkylation of **4** under the established conditions did not take place; thus, a stronger base such as NaH in acetone must be used [24].

**Scheme 6:**
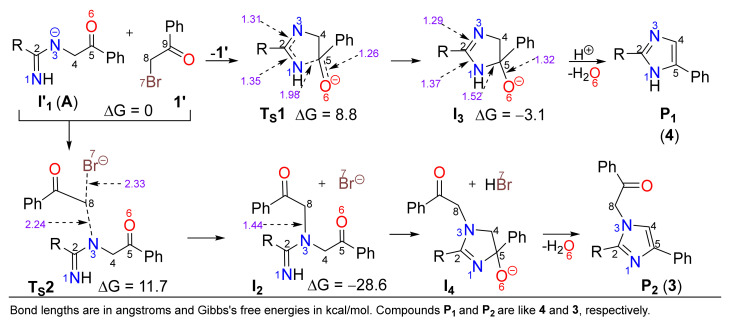
Schematic representation of the structure of critical points determined on path 1, yielding 1*H*-imidazoles (**4**, type P_1_) and path 2, leading to *N*-substituted imidazoles (**3**, type P_2_).

**Figure 2:**
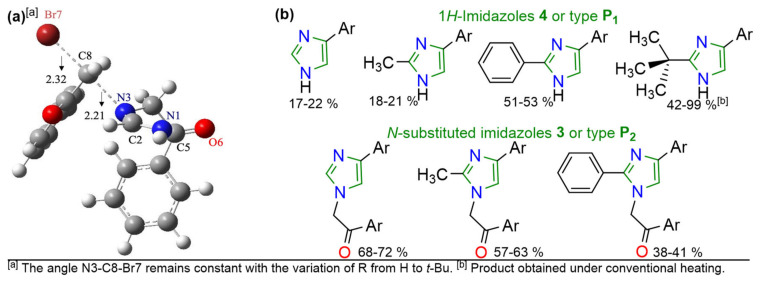
(**a**) Ball and stick representation of T_S_2 using formamidine hydrochloride (**2c**) as reagent. (**b**) General structure of imidazoles compared by the ratio of products P_1_/P_2_ or **4**/**3**.

Intrigued by the observed reactivity, we carried out a computational DFT study to unveil its molecular determinants. The computational results are summarized in Scheme 6, Figure 2, and Table 1 (see the Supporting Information for more computational details).

We considered a model system including the phenacyl bromide (**1**’) and the anion I’_1_ (A in Scheme 4). We determined that the initial reactive species is I’_1_, deriving from the deprotonation of intermediate I_1_ using the CO_3_^2−^ present in the reaction medium. This anion has a negative and nucleophilic nitrogen atom (N3). The deprotonation at sp^2^ nitrogen atom (N1) was ruled out on energy grounds (Scheme S1). The key step of the mechanism is the competence between the intramolecular cyclocondensation reaction (path 1: I’_1_ → T_S_1 → I_3_, route iia in Scheme 5) and the *N*-alkylation reaction (path 2: I’_1_ → T_S_2 → I_2_, route iib in Scheme 4), which eventually lead to 4-aryl-1*H*-imidazoles P_1_ or *N*-substituted 4-aryl-1*H*-imidazoles P_2_ (compounds 4 or 3 in Scheme 4), respectively (Scheme 6 and Figure 2).

We performed calculations of the transition states T_S_1 and Ts2 with amidines, featuring different R groups to investigate steric effects. The results, reported in Table 1 together with the experimental ratio (%P_1_/%P_2_ or **4** vs. **3**), clearly indicate that the more cumbersome R, the more favored is path1, in agreement with experimental data. Analyzing the transition states geometries, a notable change in the N1-C2-N3-C8 dihedral (137° to 105°) passing from H to *t*-Bu can be noted. These variations reflect the growing unfavorable steric interaction of the R group with the substrate **1**’, as the size of this substituent group increases (*t*-Bu > Ph > CH_3_ > H in size).

In addition, we performed calculations of the transition states T_S_1 and Ts2 with amidines featuring different R groups to investigate steric effects. The results, reported in Table 1 together with the experimental ratio (%P_1_/%P_2_ or **4** vs. **3**), clearly indicate that the more cumbersome R, the more favored is path1, in agreement with experimental data. Analyzing the transition state geometries, a notable change in the N1-C2-N3-C8 dihedral (137° to 105°) passing from H to *t*-Bu can be noted. These variations reflect the growing unfavorable steric interaction of the R group with the substrate **1**’, as the size of this substituent group increases (*t*-Bu > Ph > CH_3_ > H in size).

As expected, with lower P_1_/P_2_ (or imidazoles **4** vs. **3**) ratios (Entries 1 and 2), a small ΔΔG (T_S_1−T_S_2) was found. In particular, with R = CH_3_, the calculations predicted a very slight preference for P_1_ (instead of the experimentally observed slight preference for P_2_); we can consider this small discrepancy as an effect of the intrinsic DFT error. The different nature and solvent interaction of the two tested amidine salts (**2a/**chloride/CH_3_ vs. **2c**/acetate/H) may also cause this slight divergence. Importantly, a clear pattern is evidenced (the more cumbersome R, the more favored the P_1_ formation), which facilitates the reaction control to obtain the desired compounds, 1*H*-imidazoles or *N*-substituted imidazoles (Table 1 and Figure 2b).

Concerning the intermediates following T_S_1 and Ts2, we found that I_3_ was higher in energy than I_2_ for every combination of reactants considered, probably due to the constrained ring conformation and the negative charge on the oxygen atom. In the following steps, which are assumed not to be rate-limiting, I_3_ is stabilized by protonation of O6 and the aromaticity engendered through a water molecule elimination. On the other hand, I_2_ converts to I_4_ through an intramolecular cyclization. Consequently, P_2_ is obtained by the same final route proposed for the intermediate of cyclization I_3_, a cyclocondensation reaction with the loss of a water molecule (Scheme 6). Notably, these theoretical analyses allowed us to validate the proposed mechanism to obtain imidazoles **3** and **4** (Scheme 5).

An alternative pathway (path 3) to obtain the *N*-substituted imidazoles **3** (1,4-di- or 1,2,4-tri-substituted compounds, type P_2_) involves the interaction of the nitrogen atom of the anion A (type I’_1_) with the carbonyl group of the α-bromoketone (I’_1_ → T_S_3 → I_5_), was also examined. We found that only with formamidine (R=H), is the initial transition state along this reaction channel, T_S_3, competitive with T_S_1 and T_S_2. However, after analyzing the next steps involving second deprotonation and an intramolecular nucleophilic substitution, we could rule out path 3 on energy grounds (Scheme S2a). On the other hand, we also considered a pathway that would lead to the 1,5-di- or 1,2,5-tri-substituted regioisomers, type P_3_ (Scheme S2b). Likewise, in this case, the calculated energies were higher than those of path 1, and consequently, this pathway is not competitive (Table S1, Supplementary Materials).

## 3. Materials and Methods

### 3.1. General

Synthesis and melting points. Reagents were purchased from commercial sources and used without further purification. All starting materials were weighed and handled in the presence of air at room temperature. Reactions were monitored by thin-layer chromatography (TLC) visualized by a UV lamp (254 nm or 365 nm). Flash chromatography was performed on silica gel (230–400 mesh). MW-assisted reactions were performed in a CEM Discover SP-focused microwave (ν = 2.45 GHz) reactor bearing a built-in pressure measurement sensor and a vertically focused IR temperature sensor. Sealed reaction vessels (10 mL, max pressure = 300 psi) containing a Teflon-coated stir bar (obtained from CEM) were used for these reactions. The temperature, power, and time settings were controlled and used for all reactions. Melting points were conducted using a capillary melting point apparatus and are uncorrected.

Characterization methods. NMR spectroscopic data were performed in a Bruker Avance 400 (Universidad de los Andes, Bogotá, Colombia) at 298 K using TMS (0.00 ppm) or the residual non-deuterated solvent as the internal reference. ^1^H (400 MHz) and ^13^C (101 MHz) NMR spectroscopic data were recorded in CDCl_3_ (δ_H_ = 7.26 ppm/δ_C_ = 77.0 ppm), DMSO-*d*_6_ (δ_H_ = 2.50 ppm/δ_C_ = 39.5 ppm), or CD_3_OD (δ_C_ = 49.0 ppm). DEPT-135 spectra were used to assign the carbon signals. Chemical shifts (δ) were reported in ppm, and the coupling constants (*J*) in Hz. The following abbreviations were used for multiplicities: s = singlet, d = doublet, t = triplet, and m = multiplet. The high-resolution mass spectra (HRMS) were recorded using an Agilent Technologies Q-TOF 6520 spectrometer (Universidad de los Andes, Bogotá, Colombia) by electrospray ionization (ESI). Aroylmethyl bromides **1a–d** were prepared by methods developed in our laboratory [24,27].

### 3.2. General Procedures

*Synthesis of 4-aryl-2-phenylimidazoles***3e–h***and***4f–g**. A mixture of benzamidine hydrochloride (**2b**, 82 mg, 0.52 mmol), potassium carbonate anhydrous (K_2_CO_3_, 141 mg, 1.02 mmol), and the appropriate aroylmethyl bromide **1a–d** (1.00 mmol) in acetonitrile anhydrous (1.0 mL) was subjected to microwave irradiation at 100 °C (150 W, monitored by an IR temperature sensor) and maintained at this temperature for 40 min in a sealed tube containing a Teflon-coated magnetic stirring bar. The resulting reaction mixture was cooled to 50 °C by airflow and was neutralized with dilute hydrochloric acid (HCl, 10%) and then partitioned between water (5.0 mL) and ethyl acetate (2 × 10.0 mL). The organic layer was washed with brine (2 × 5.0 mL) and dried over anhydrous magnesium sulfate (MgSO_4_). Then, the solvent was removed under reduced pressure, and the residue purified by flash chromatography on silica gel (eluent: first CH_2_Cl_2_ and then CH_2_Cl_2_/CH_3_OH 20:1 *v*/*v*). The first fraction eluted contained *N*-aroylmethylimidazoles **3e–h** (minor product, 38–41%), while the second fraction contained 1*H*-imidazoles **4b–c** (major product, 51–53%).

*Synthesis of 4-arylimidazoles***3i–l***and***4i–j**, *and aroylmethyl acetates***5a–d**. A mixture of formamidinium acetate (**2c**, 54 mg, 0.52 mmol), K_2_CO_3_ anhydrous (141 mg, 1.02 mmol), and the appropriate substrate **1a–d** (1.00 mmol) in acetonitrile anhydrous (1.0 mL) was subjected to microwave irradiation at 120 °C (150 W, monitored by an IR temperature sensor) and maintained at this temperature for 40 min in a sealed tube containing a Teflon-coated magnetic stirring bar. The reaction treatment and purification of the products were carried out in the same way as in the previous reaction. The first fraction eluted by flash chromatography contained *N*-aroylmethylimidazoles **3i–l** (major product, 68–72%), while the second fraction contained compounds **4i–j** (minor product, 17–22%). Aroylmethyl acetates **5a–d** were obtained when this reaction was carried out at 100 °C under similar conditions; however, the residue obtained was purified by flash chromatography (eluent: CH_2_Cl_2_) to afford the pure products **5a–d** in high yields (82–87%).

### 3.3. Characterization Data

*4-(4-Fluorophenyl)-1-(2-(4-fluorophenyl)-2-etanone)-2-phenyl-1H-imidazole* (**3e**): Light yellow (77 mg, 41%, 0.5 mmol); mp 279–280 °C. ^1^H NMR (400 MHz, CD_3_OD): *δ* = 5.54 (s, 2H), 7.01 (t, *J* = 8.8 Hz, 2H), 7.16 (t, *J* = 8.7 Hz, 2H), 7.34–7.43 (m, 6H), 7.70 (m, 2H), 7.99 (m, 2H) ppm. ^13^C{^1^H} NMR (101 MHz, CD_3_OD): *δ* = 54.3 (CH_2_), 116.2/116.4 (d, *J* = 22.0 Hz, CH), 117.0/117.2 (d, *J* = 22.7 Hz, CH), 127.8/127.9 (d, *J* = 8.8 Hz, CH), 129.9 (CH), 130.1 (CH), 130.7 (CH), 131.1 (C), 131.6 (d, *J* = 2.9 Hz, C), 132.2/132.3 (d, *J* = 8.8 Hz, CH), 132.3 (d, *J* = 3.0 Hz, C), 141.0 (C), 150.6 (C), 162.2/164.7 (d, *J* = 241.3 Hz), 166.5/169.0 (d, *J* = 254.6 Hz), 193.2 (C=O) ppm. HRMS (ESI+): calcd. for C_23_H_17_F_2_N_2_O^+^ 375.1303 [M + H]^+^; found 375.1311.

*4-(4-Chlorophenyl)-1-(2-(4-chlorophenyl)-2-etanone)-2-phenhyl-1H-imidazole* (**3f**): Light white (77 mg, 38%, 0.5 mmol); mp >300 °C. ^1^H NMR (400 MHz, CD_3_OD): *δ* = 5.86 (s, 3H), 7.46–7.56 (m, 6H), 7.59–7.63 (m, 3H), 7.70 (d, *J* = 8.5 Hz, 2H), 7.93 (m, 3H) ppm. ^13^C{^1^H} NMR (101 MHz, CD_3_OD): *δ* = 55.8 (CH_2_), 121.9 (CH), 128.5 (CH), 130.5 (CH), 130.6 (CH), 130.8 (CH), 130.9 (CH), 131.1 (CH), 132.2 (C), 133.7 (C), 134.1 (C), 136.2 (C), 142.3 (C), 156.9 (C), 191.3 (C=O) ppm. HRMS (ESI+): calcd. for C_23_H_17_^35^C_l2_N_2_O^+^ 407.0712 [M + H]^+^; found 407.0715.

*4-(4-Bromophenyl)-1-(2-(4-bromophenyl)-2-etanone)-2-phenhyl-1H-imidazole* (**3g**): Light white (99 mg, 40%, 0.5 mmol); mp >300 °C. ^1^H NMR (400 MHz, CD_3_OD): *δ* = 5.77 (s, 2H), 7.46–7.58 (m, 7H), 7.63 (m, 4H), 7.83 (m, 3H), ppm. ^13^C{^1^H} NMR (101 MHz, CD_3_OD): *δ* = 55.8 (CH_2_), 121.9 (CH), 123.6 (C), 125.1 (C), 127.0 (C), 128.6 (CH), 130.2 (CH), 130.8 (CH), 131.0 (C), 131.1 (CH), 133.6 (CH), 133.8 (CH), 134.0 (CH), 134.1 (C), 148.7 (C), 157.6 (C), 191.6 (C=O) ppm. HRMS (ESI+): calcd. for C_23_H_17_^79^Br_2_N_2_O^+^ 494.9702 [M + H]^+^; found 494.9704.

*4-(4-Methoxyphenyl)-1-(2-(4-methoxyphenyl)-2-etanone)-2-phenhyl-1H-imidazole *(**3h**): Light yellow (76 mg, 38%, 0.5 mmol); mp 199–200 °C. ^1^H NMR (400 MHz, DMSO-*d*_6_): *δ* = 2.33 (s, 3H), 5.25 (s, 2H), 7.06 (s, 1H), 7.30 (d, J = 8.5 Hz, 2H), 7.52 (d, *J* = 8.6 Hz, 2H), 7.66 (d, *J* = 8.5 Hz, 2H), 7.91 (d, *J* = 8.6 Hz, 2H) ppm. 13C{1H} NMR (101 MHz, DMSO-*d*_6_): *δ* = 53.2 (CH_2_), 55.1 (CH_3_), 55.7 (CH_3_), 114.0 (CH), 114.3 (CH), 118.6 (CH), 125.6 (CH), 127.0 (C), 127.1 (C), 128.0 (CH), 128.6 (CH), 128.7 (CH), 130.5 (CH), 130.6 (C), 139.5 (C), 147.4 (C), 158.1 (C), 163.9 (C), 191.4 (C=O) ppm. HRMS (ESI+): calcd. for C_25_H_23_N_2_O_3_^+^ 399.1703 [M + H]^+^; found 399.1695.

*4-(4-Fluorophenyl)-1-(2-(4-fluorophenyl)-2-etanone)-1H-imidazole* (**3i**): White solid (107 mg, 72%, 0.5 mmol); mp 242–243 °C. ^1^H NMR (400 MHz, DMSO-*d*_6_): *δ* = 5.76 (s, 2H), 7.18 (t, *J* = 8.7 Hz, 2H), 7.44 (t, *J* = 8.7 Hz, 2H), 7.55 (s, 1H), 7.67 (s, 1H), 7.77 (m, 2H), 8.14 (m, 2H) ppm. ^13^C{^1^H} NMR (101 MHz, DMSO-*d*_6_): *δ* = 52.9 (CH_2_), 117.1 (CH), 115.3/115.5 (d, *J* = 21.3 Hz, CH), 116.0/116.2 (d, *J* = 22.0 Hz, CH), 126.0/126.1 (d, *J* = 7.3 Hz, CH), 131.1/131.2 (d, *J* = 9.6 Hz, CH), 131.3 (d, *J* = 3.7 Hz, C), 132.2 (d, *J* = 3.7 Hz, C), 139.0 (CH), 139.2 (C), 162.4/164.9 (d, *J* = 244.0 Hz, C), 166.6/169.2 (d, *J* = 259.7 Hz, C), 192.2 (C=O) ppm. HRMS (ESI+): calcd. for C_17_H_13_F_2_N_2_O^+^ 299.0990 [M + H]^+^; found 299.0997.

*4-(4-Chlorophenyl)-1-(2-(4-chlorophenyl)-2-etanone)-1H-imidazole* (**3j**): Yellow solid (116 mg, 70%, 0.5 mmol); Yield 116 mg (0.5 mmol), 70%; mp 254–255 °C. ^1^H NMR (400 MHz, DMSO-*d*_6_): *δ* = 5.78 (s, 2H), 7.61 (m, 3H), 7.70 (m, 3H), 8.01 (d, *J* = 8.5 Hz, 2H), 8.07 (d, *J* = 8.8 Hz, 2H) ppm. ^13^C{^1^H} NMR (101 MHz, DMSO-*d*_6_): *δ* = 53.0 (CH_2_), 117.7 (C), 128.3 (CH), 129.9 (CH), 130.0 (CH), 130.7 (CH), 133.2 (C), 133.1 (C), 136.5 (C), 137.5 (C), 139.0 (C), 139.2 (C), 192.6 (C=O) ppm. HRMS (ESI+): calcd. for C_17_H_13_^35^Cl_2_N_2_O^+^ 331.0399 [M + H]^+^; found 331.0400.

*4-(4-Bromophenyl)-1-(2-(4-bromophenyl)-2-etanone)-1H-imidazole* (**3k**): Light white (143 mg, 68%, 0.5 mmol); mp 163–164 °C. ^1^H NMR (400 MHz, DMSO-*d*_6_): *δ* = 5.77 (s, 2H), 7.54 (d, *J* = 8.3 Hz, 2H), 7.63 (s, 1H), 7.71 (d, *J* = 8.3 Hz, 2H), 7.84 (m, 3H), 7.99 (d, *J* = 8.5 Hz, 2H), ppm. ^13^C{^1^H} NMR (101 MHz, DMSO-*d*_6_): *δ* = 52.9 (CH_2_), 117.8 (CH), 118.9 (CH), 126.2 (CH), 128.2 (C), 130.0 (CH), 131.4 (CH), 132.1 (CH), 133.4 (C), 133.8 (C), 139.0 (C), 139.2 (CH), 192.8 (C=O) ppm. HRMS (ESI+): calcd. for C_17_H_13_^79^Br_2_N_2_O^+^ 418.9389 [M + H]^+^; found 418.9383.

*4-(4-Methoxyphenyl)-1-(2-(4-methoxyphenyl)-2-etanone)-1H-imidazole* (**3j**): Yellow solid (114 mg, 71%, 0.5 mmol); mp 199–200 °C. ^1^H NMR (400 MHz, DMSO-*d*_6_): *δ* = 3.75 (s, 3H), 3.86 (s, 3H), 5.68 (s, 2H), 6.92 (d, *J* = 8.8 Hz, 2H), 7.11 (d, *J* = 8.7 Hz, 2H), 7.43 (s, 1H), 7.60 (s, 1H), 7.66 (d, *J* = 8.8 Hz, 2H), 8.02 (d, *J* = 8.7 Hz, 2H) ppm. ^13^C{^1^H} NMR (101 MHz, DMSO-*d*_6_): *δ* = 52.5 (CH_2_), 55.0 (CH_3_), 55.7 (CH_3_), 113.9 (CH), 114.2 (CH), 116.0 (CH), 125.4 (CH), 126.5 (C), 126.6 (C), 130.4 (CH), 138.7 (CH), 140.0 (C), 157.8 (C), 163.7 (C), 191.9 (C=O) ppm. HRMS (ESI+): calcd. for C_19_H_19_N_2_O_3_^+^ 323.1390 [M + H]+; found 323.1401.

*4-(4-Chlorophenyl)-2-phenhyl-1H-imidazole* (**4f**): White solid. Yield 68 mg (0.5 mmol), 53%; mp 269–271 °C (Lit. [28] 273–275 °C). ^1^H NMR (400 MHz, CDCl_3_): *δ* = 7.33–7.43 (m, 6H), 7.70 (d, *J* = 8.3 Hz, 2H), 7.85 (d, *J* = 8.1 Hz, 2H) ppm, NH is absent. ^13^C{^1^H} NMR (101 MHz, CDCl_3_): *δ* = 125.3 (CH), 126.2 (CH), 128.8 (CH), 128.9 (CH), 128.9 (CH), 129.0 (CH), 129.9 (C), 131.7 (C), 132.6 (C), 147.2 (C) and 147.3 (C) ppm. These NMR data match previously reported data [29].

*4-(4-Bromophenyl)-2-phenhyl-1H-imidazole* (**4g**): White solid. Yield 76 mg (0.5 mmol), 51%; mp 179–180 °C (Lit. [30] 179–181 °C). ^1^H NMR (400 MHz, DMSO-*d*_6_): *δ* = 7.37 (t, *J* = 8.0, 1H), 7.47 (t, *J* = 8.0 Hz, 2H), 7.56 (d, *J* = 8, 2H), 7.81 (m, 3H), 7.99 (d, *J* = 8.0, 2H) ppm, NH is absent. ^13^C{^1^H} NMR (101 MHz, DMSO-d6): = 114.8 (CH), 118.9 (C), 124.9 (CH), 126.4 (CH), 128.2 (CH), 128.7 (CH), 129.2 (C), 130.4 (C), 131.3 (CH), 133.9 (C), 146.1 (C) ppm. These NMR data match previously reported data [30].

*4-(4-Fluorophenyl)-1H-imidazole* (**4i**): Light yellow. 18 mg (0.5 mmol), 22%; mp 173–175 °C (Lit. [31] 125–126 °C). Major tautomer: ^1^H NMR (400 MHz, DMSO-*d*_6_): *δ* = 7.32–7.36 (m, 2H), 7.88 (s, 1H), 7.92–8.07 (m, 1H), 8.37 (m, 2H), 12.8 (br s, 1H) ppm. ^13^C{^1^H} NMR (101 MHz, DMSO-*d*_6_): *δ* = 115.0 (CH, *J* = 22.7 Hz), 124.7 (CH), 132.7 (CH, *J* = 7.3 Hz), 134.3 (C, *J* = 2.9 Hz), 136.6 (CH), 140.5 (C) and 164.4 (C, *J* = 25.1 Hz) ppm. Minor tautomer: ^1^H NMR (400 MHz, DMSO-*d*_6_): *δ* = 7.14–7.20 (m, 2H), 7.32–7.36 (m, 1H), 7.62–7.77 (m, 2H), 7.92–8.07 (m, 1H), 13.3 (br s, 1H) ppm. These NMR data match previously reported data [31].

*4-(4-Chlorophenyl)-1H-imidazole* (**4j**): Light yellow. Yield 15 mg (0.5 mmol), 17%; mp 163–165 °C (Lit. [32] 135–136 °C). ^1^H NMR (400 MHz, DMSO-*d*_6_): *δ* = 7.39–8.27 (m, 6H), 12.7 (br s, 1H, NH) ppm. ^13^C{^1^H} NMR (101 MHz, DMSO-*d*_6_): *δ* = 125.0 (CH), 125.8 (CH), 128.2 (CH), 131.8 (CH), 136.8 (C), 138.0 (C) and 140.4 (C) ppm. These NMR data match previously reported data [32].

*2-(4-Fluorophenyl)-2-oxoethyl acetate* (**5a**): Light yellow (89 mg, 87%); mp 48–50 °C (Lit. [33] 48–50 °C). ^1^H NMR (400 MHz, CDCl_3_): *δ* = 2.23 (s, 3H), 5.31 (s, 2H), 7.14–7.19 (m, 2H), 7.93–7.97 (m, 2H) ppm. ^13^C{^1^H} NMR (101 MHz, CDCl_3_): *δ* = 20.6 (CH_3_), 65.8 (CH_2_), 116.1 (CH, *J* = 22.0 Hz), 130.3 (C, *J* = 2.9 Hz), 130.5 (CH, *J* = 9.5 Hz), 166.2 (C, *J* = 256.0 Hz), 170.5 (C=O) and 190.8 (C=O) ppm. These NMR data matched previously reported data [33].

*2-(4-Chlorophenyl)-2-oxoethyl acetate* (**5b**): White solid (94 mg, 85%); mp 68–70 °C (Lit. [33] 70–72 °C). ^1^H NMR (400 MHz, CDCl_3_): *δ* = 2.20 (s, 3H), 5.27 (s, 2H), 7.44 (d, *J* = 8.8 Hz, 2H), 7.83 (d, *J* = 8.8 Hz, 2H) ppm. ^13^C{^1^H} NMR (101 MHz, CDCl_3_): *δ* = 20.4 (CH_3_), 65.7 (CH_2_), 129.0 (CH), 129.1 (CH), 132.4 (C), 140.2 (C), 170.2 (C=O) and 191.0 (C=O) ppm. These NMR data matched previously reported data [33].

*2-(4-Bromophenyl)-2-oxoethyl acetate* (**5c**): Yellow solid (110 mg, 82%); mp 81–83 °C (Lit. [33] 72–74 °C). ^1^H NMR (400 MHz, CDCl_3_): *δ* = 2.20 (s, 3H), 5.27 (s, 2H), 7.44 (d, *J* = 8.8 Hz, 2H), 7.83 (d, *J* = 8.8 Hz, 2H) ppm. ^13^C{^1^H} NMR (101 MHz, CDCl_3_): *δ* = 20.4 (CH_3_), 65.7 (CH_2_), 129.0 (CH), 129.1 (CH), 132.4 (C), 140.2 (C), 170.2 (C=O) and 191.0 (C=O) ppm. These NMR data matched previously reported data [33].

*2-(4-Methoxyphenyl)-2-oxoethyl acetate* (**5d**): Yellow solid (94 mg, 87%); mp 58–59 °C (Lit. [34] 60–61 °C). ^1^H NMR (400 MHz, CDCl_3_): *δ* = 2.22 (s, 3H), 3.87 (s, 3H), 5.29 (s, 2H), 6.95 (d, 2H), 7.89 (d, 2H) ppm. ^13^C{^1^H} NMR (101 MHz, CDCl_3_): *δ* = 20.6 (CH_3_), 55.5 (CH_3_), 65.8 (CH_2_), 114.1 (CH), 127.2 (C), 130.1 (CH), 164.1 (C), 170.5 (C=O) and 190.6 (C=O) ppm. These NMR data matched previously reported data [34].

## 4. Conclusions

To sum up, we synthesized a novel family of *N*-aroylmethyl-4-arylimidazoles **3e–l** via a pseudo-three-component reaction that proceeds with the formation of three new C–N bonds in a regioselective manner. Products were obtained via the reaction of aroylmethyl bromides with amidine salts in a 2:1 ratio; however, the bicomponent reaction (1:1) that allows 1*H*-imidazoles **4** to be formed also occurred. This protocol provides high yields (up to 94% for the mixture of **3** and **4**) using K_2_CO_3_ as a base in acetonitrile under microwave irradiation conditions. Remarkably, the proportion of imidazoles obtained, **3** and **4,** is governed by steric factors of the R group attached to the starting amidine, which together with the proposed reaction mechanism, was validated by DFT calculations; a clear pattern was evidenced: the smaller the R group, the more favored is the formation of the *N*-substituted products **3** (type **P_2_**). In general, the computational and experimental studies were positively complemented to better explain and understand our interesting findings.

## Figures and Tables

**Table 1:** Difference between transition state energies for model products P_1_/P_2_ and ratio of products **4**/**3** [a].

Entry	Substituent (R)	ΔΔG(T_S_1/P_1_–T_S_2/P_2_) (kcal/mol)	Ratio 4/3 (Total Yield %)
**1**	H	0.85	1:3.5 (90) [b]
**2**	CH_3_	−0.78	1:3.0 (81) [b]
**3**	Ph	−2.84	1.25:1 (91) [b]
**4**	*t*-Bu	−7.12	42/99: −/− (42/99) [c]

[a] Compounds type P_1_ are 1*H*-imidazoles 4 and type P_2_ *N*-substituted imidazoles 3. [b] Ratio and approximate total yield of the mixture. Products type P**_2_** were only obtained by us under MW [24,25,26]. [c] Only 1*H*-imidazoles were isolated [21,22].

## Data Availability

The article or Supplementary Materials contains the data for this investigation.

## Supplementary Materials

Supporting information for this article (i.e., copies of NMR spectra, HRMS analysis, and computational details) can be downloaded. Scheme S1: Two possibilities deprotonation routes of I_1_ using K_2_CO_3_; Scheme S2: Possible pathways leading to N-substituted imidazoles P_2_ and P_3_; Table S1: Energies of key transition states in the discussed mechanism of reaction; Figure S1: Reaction profile comparison between the N1-alkylation (→C-Br, blue), N-Addition (→C=O, green), and N3-alkylation mechanisms. The strong black line indicates the change in PES.

## Sample Availability

Samples of the compounds are available from the authors.
