# Supplementary material for: Influence of Steric Effect on the Pseudo-Multicomponent Synthesis of N-Aroylmethyl-4-Arylimidazoles
Source: Molecules. 2022 Feb 9;27(4):1165. doi: 10.3390/molecules27041165 (PMC8874432; doi:10.3390/molecules27041165)
Supplement: Supplementary file 1 [file molecules-27-01165-s001.zip › molecules-1548969-supplementary.pdf]

Steric effect influence on the *pseudo*-multicomponent synthesis of 4-aryl-*N*-phenacylimidazoles

Nerith-Rocio Elejalde[a], Mayra García-Olave[a], David Figueroa[b], Pietro Vidossich[c], Gian Pietro Miscione[b], and Jaime Portilla[a*]

[a] *Bioorganic Compounds Research Group, Department of Chemistry, Universidad de los Andes, Carrera 1 No. 18A-10, 111711 Bogotá, Colombia*

[b] *COBO - Computational Bio-Organic Chemistry Bogotá, Department of Chemistry, Universidad de los Andes, Cra 1 No. 18A-12, 111711 Bogotá, Colombia*

[c] *Laboratory of Molecular Modeling and Drug Discovery, Istituto Italiano di Tecnologia, Via Morego 30, 16163, Genova, Italy*

* Correspondence: jportill@uniandes.edu.co; Tel.: +571-3394949

SUPPORTING INFORMATION

Content:

1. **Overview of substrates and products numbering ……………….……..p. S2**
2. **Copies of NMR spectra for substituted imidazoles 3a-l ……….............p. S3**
3. **HRMS analysis substituted imidazoles 3a-l ……………………………p. S11**
4. **Computational details ……………………..……………………………p. S15**

1. Overview of substrates and products numbering

Substrates and reagents

α-Bromoacetophenones **1a-d** and amidine derivatives **2b** and **2c**

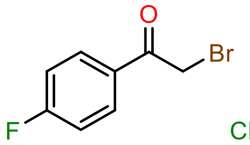
1a

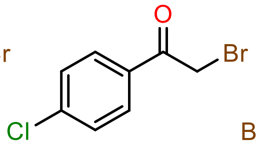
1b

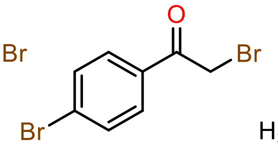
1c

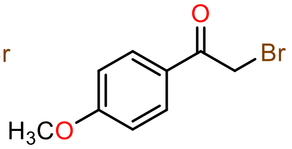
1d

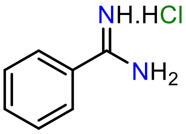
2b

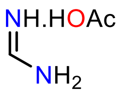
2c

Products

N-Phenacylimidazoles **3e-l**, *NH*-imidazoles **4f**/**4g**/**4i**/**4j**, and α-acetoxyacetophenones **5a-d**

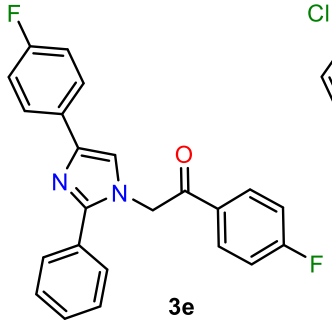
3e

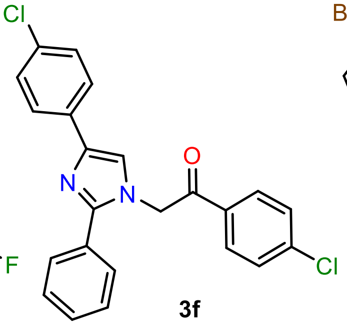
3f

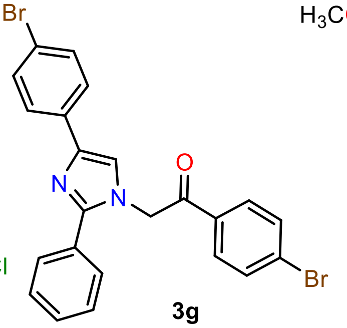
3g

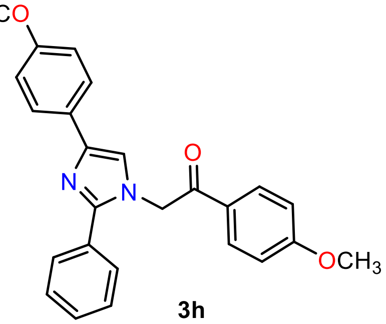
3h

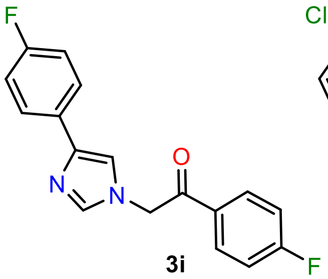
3i

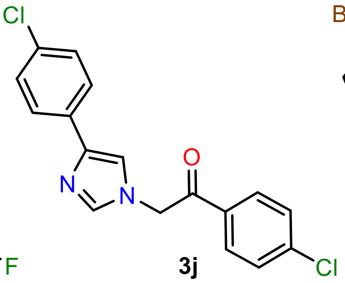
3j

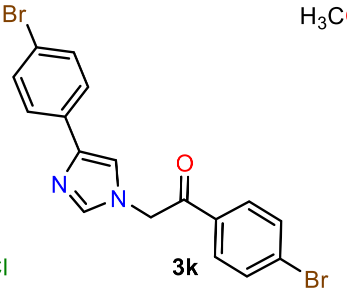
3k

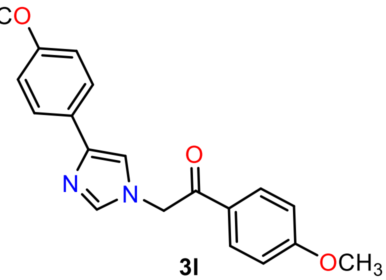
3l

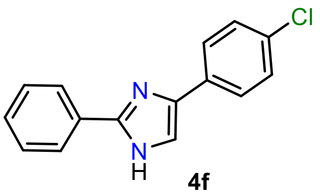
4f

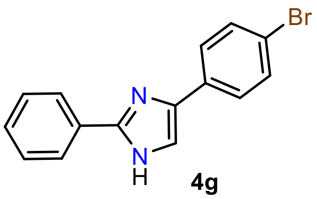
4g

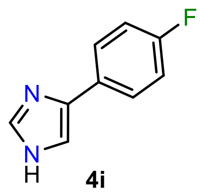
4i

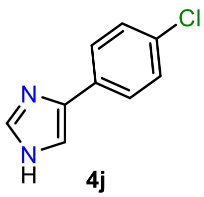
4j

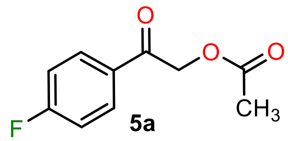
5a

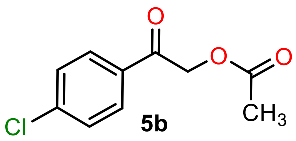
5b

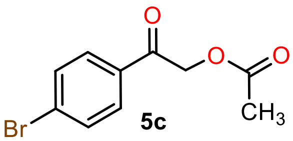
5c

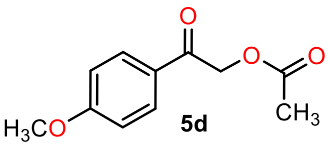
5d

2. Copies of NMR spectra for substituted imidazoles 3e-l

^{1}H and $^{13}C\{^{1}H\}$ NMR spectra of 1-(4-fluorophenyl)-2-(4-(4-fluorophenyl)-2-phenyl-1*H*-imidazol-1-yl)ethan-1-one **3e**

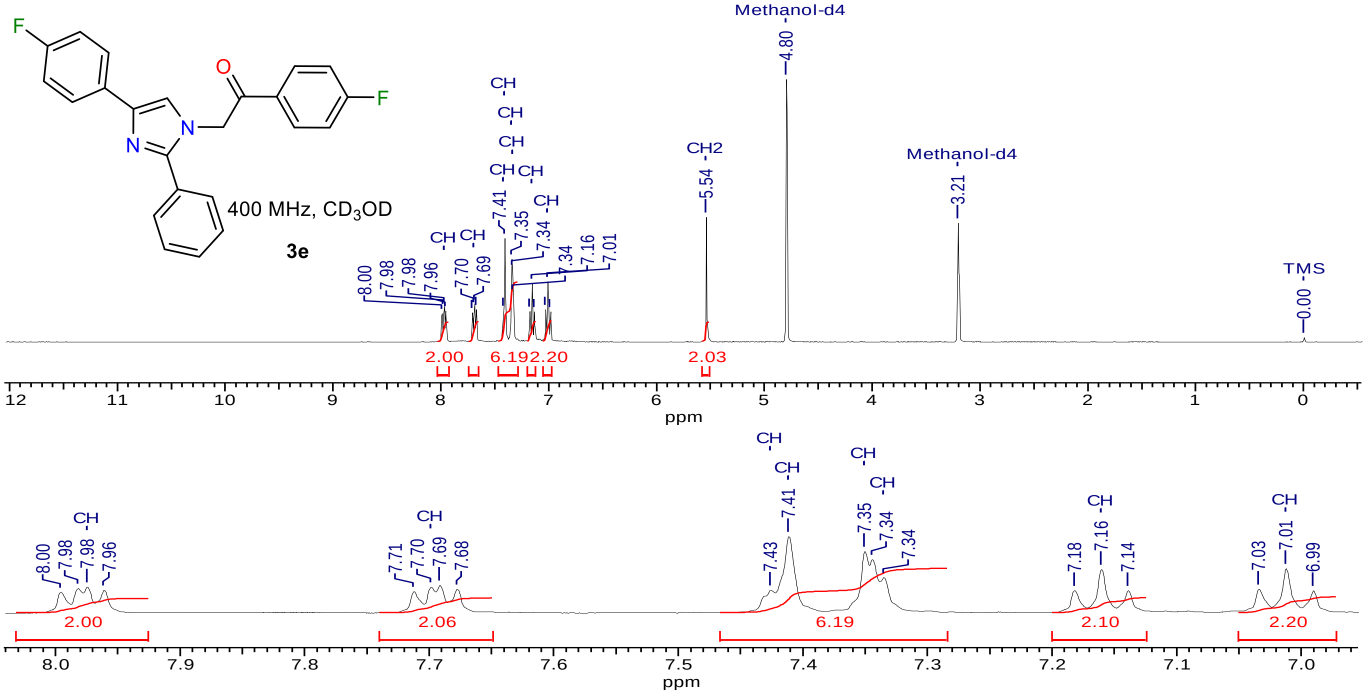

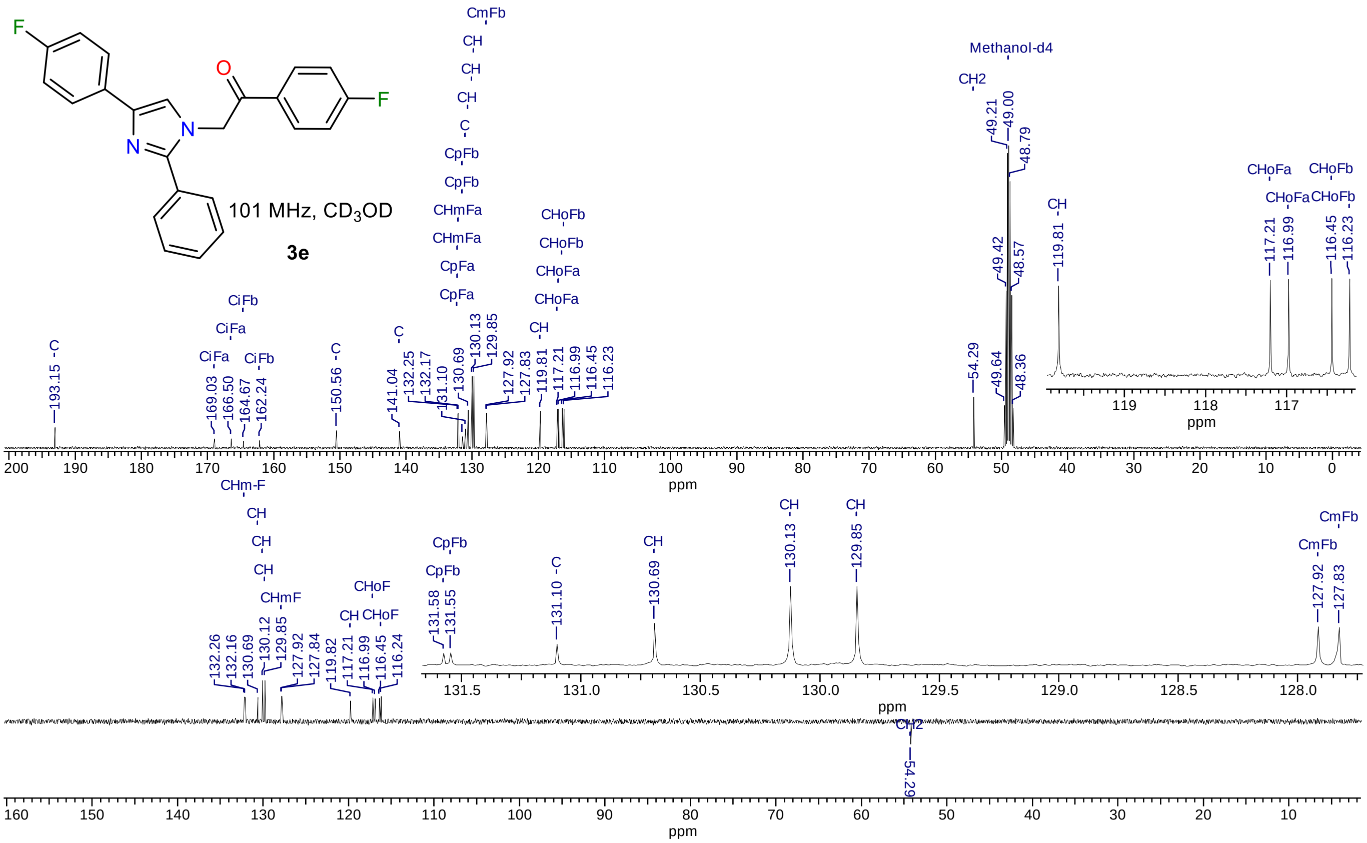

^{1}H and ^{13}C{^{1}H} NMR spectra of 1-(4-chlorophenyl)-2-(4-(4-chlorophenyl)-2-phenyl-1*H*-imidazol-1-yl)ethan-1-one **3f**

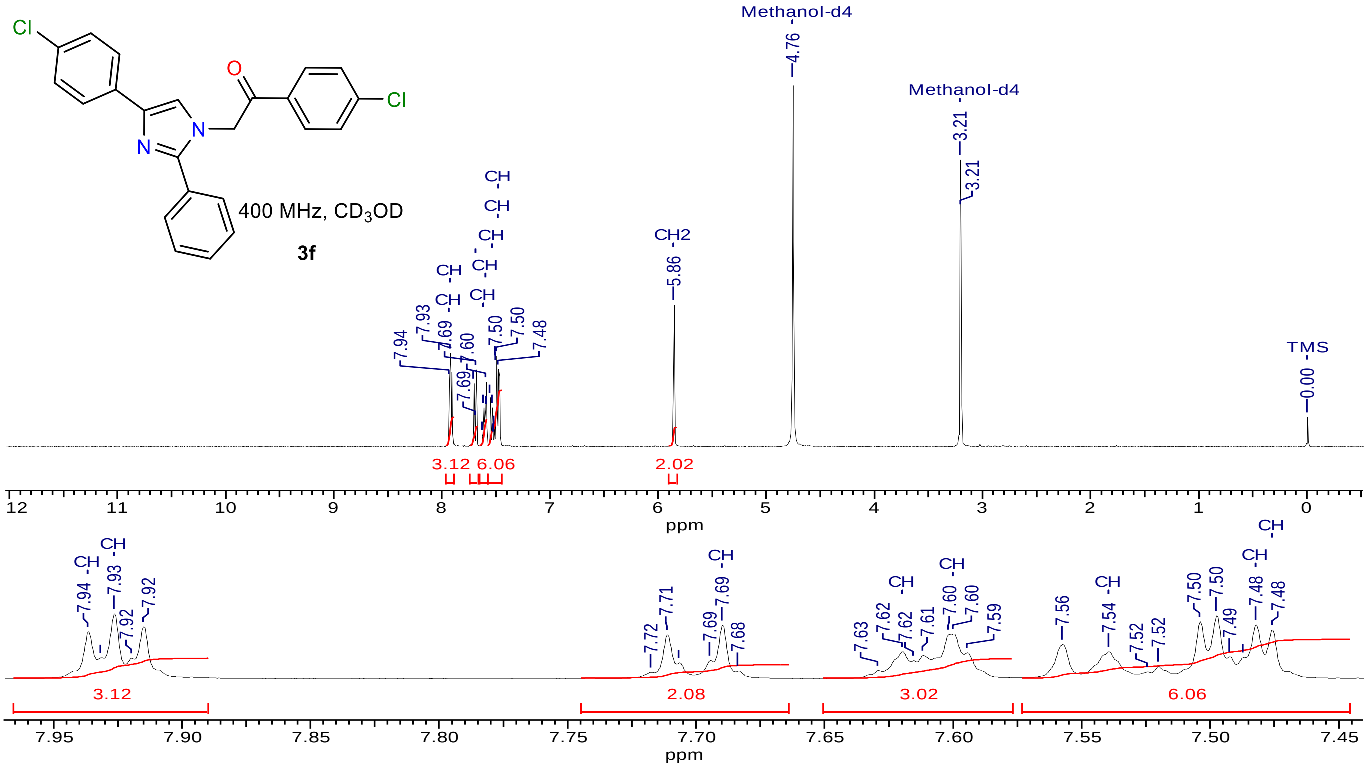

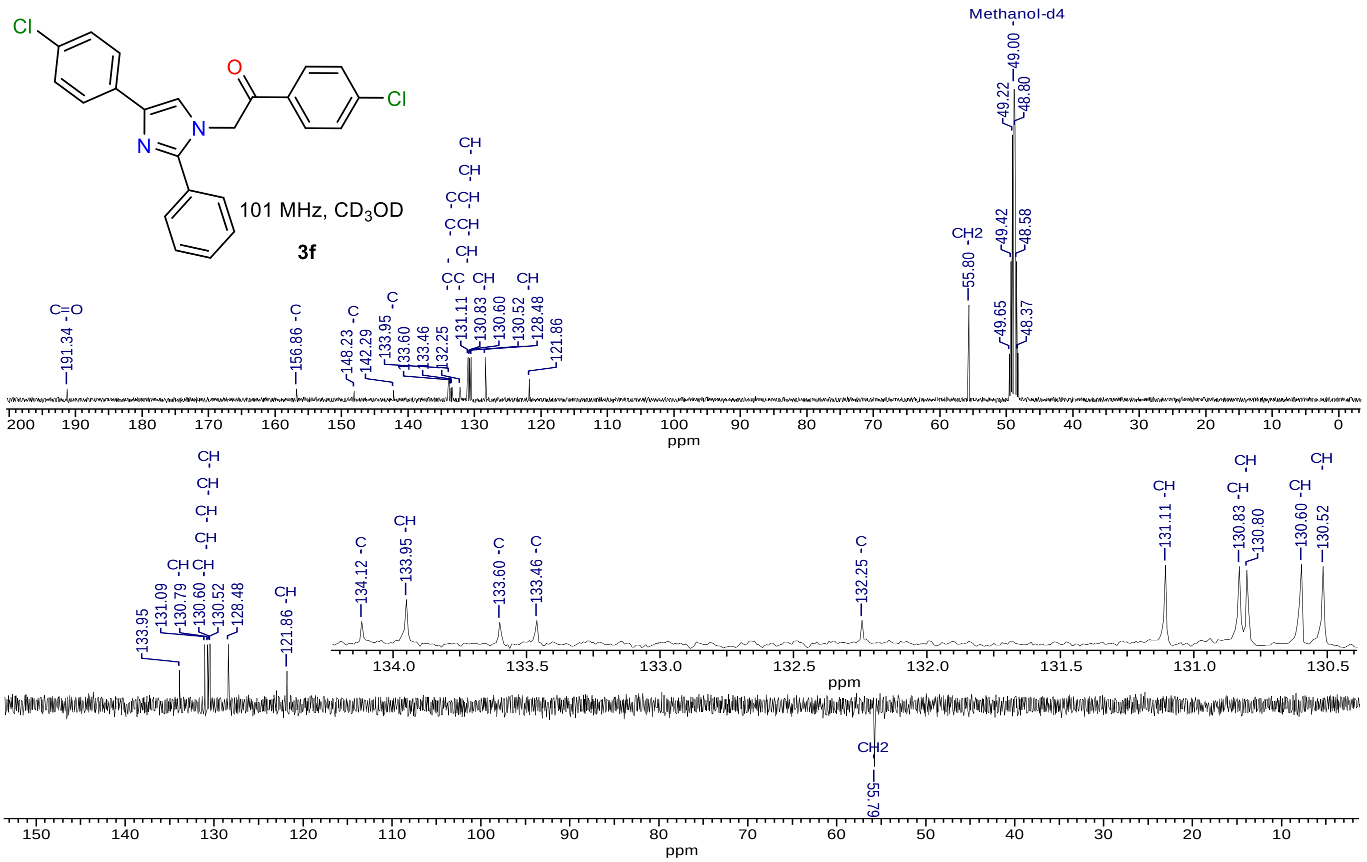

^{1}H and ^{13}C{^{1}H} NMR spectra of 1-(4-bromophenyl)-2-(4-(4-bromophenyl)-2-phenyl-1*H*-imidazol-1-yl)ethan-1-one **3g**

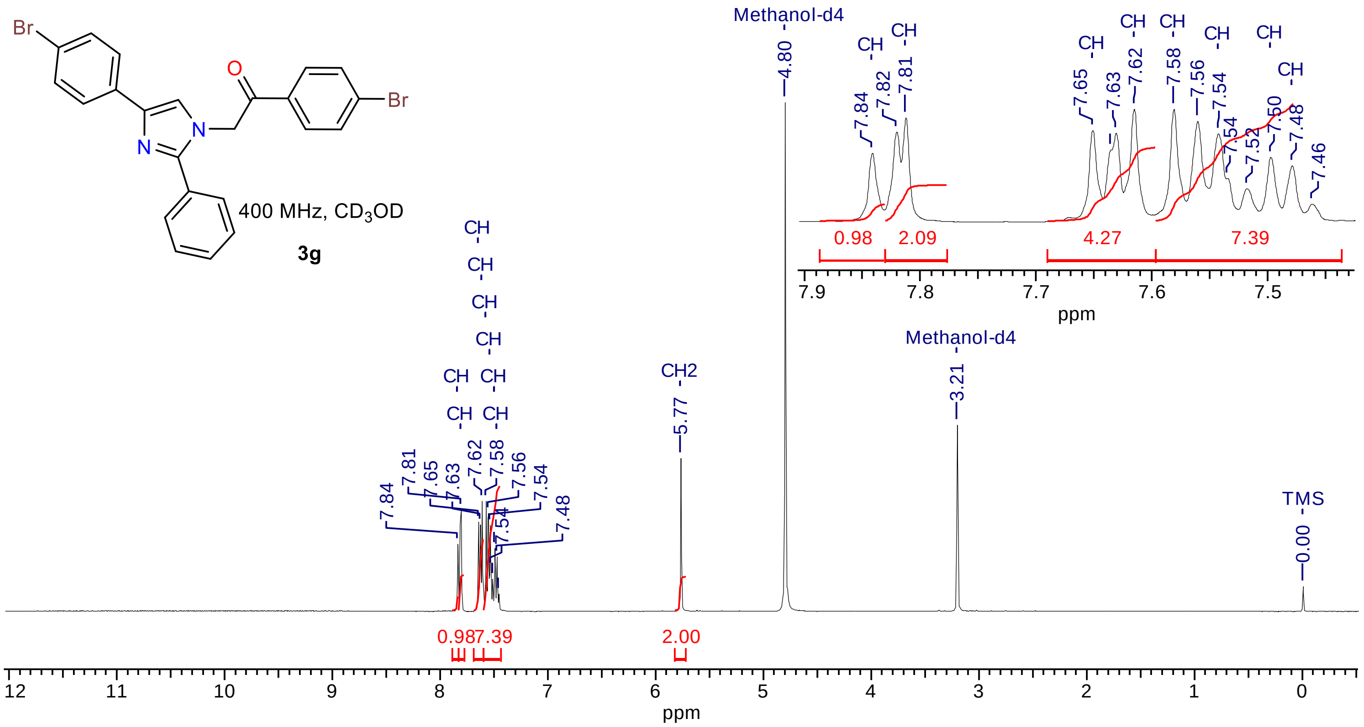

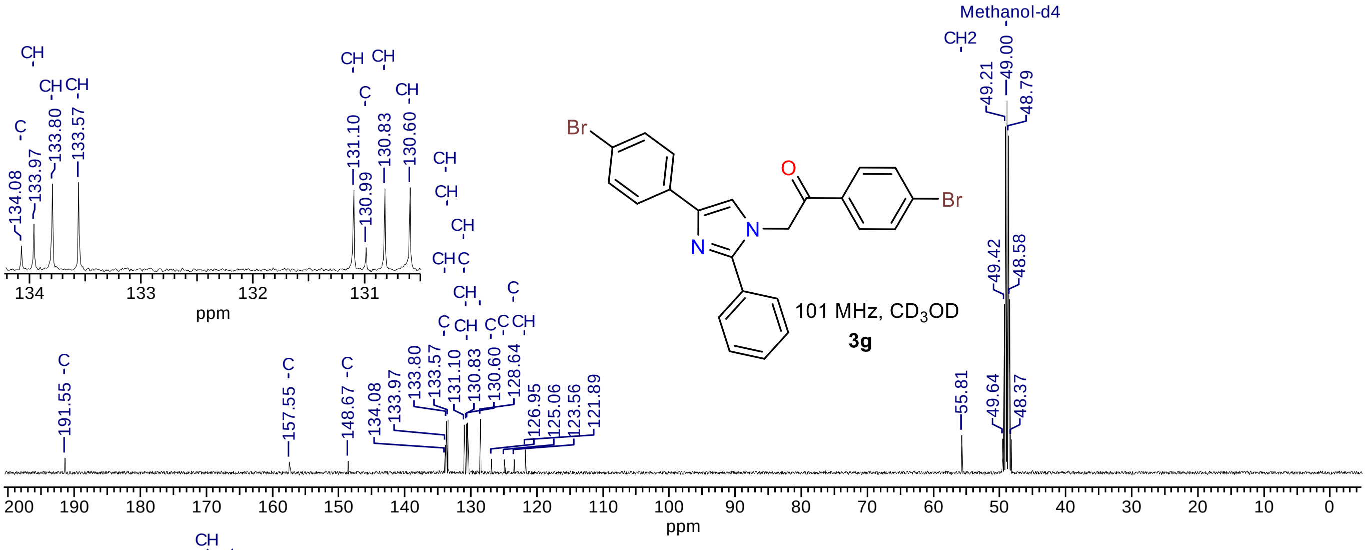

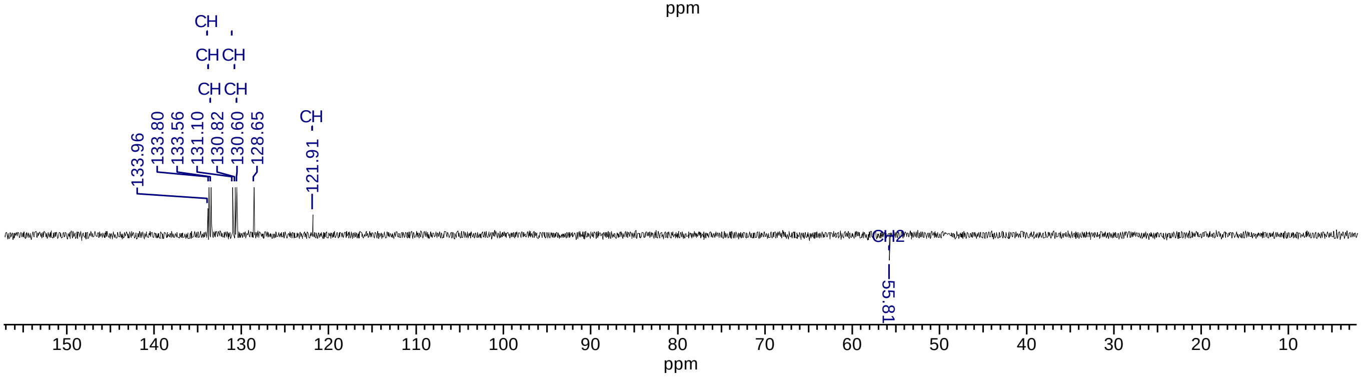

^{1}H and ^{13}C{^{1}H} NMR spectra of 1-(4-methoxyphenyl)-2-(4-(4-methoxyphenyl)-2-phenyl-1*H*-imidazol-1-yl)ethan-1-one **3h**

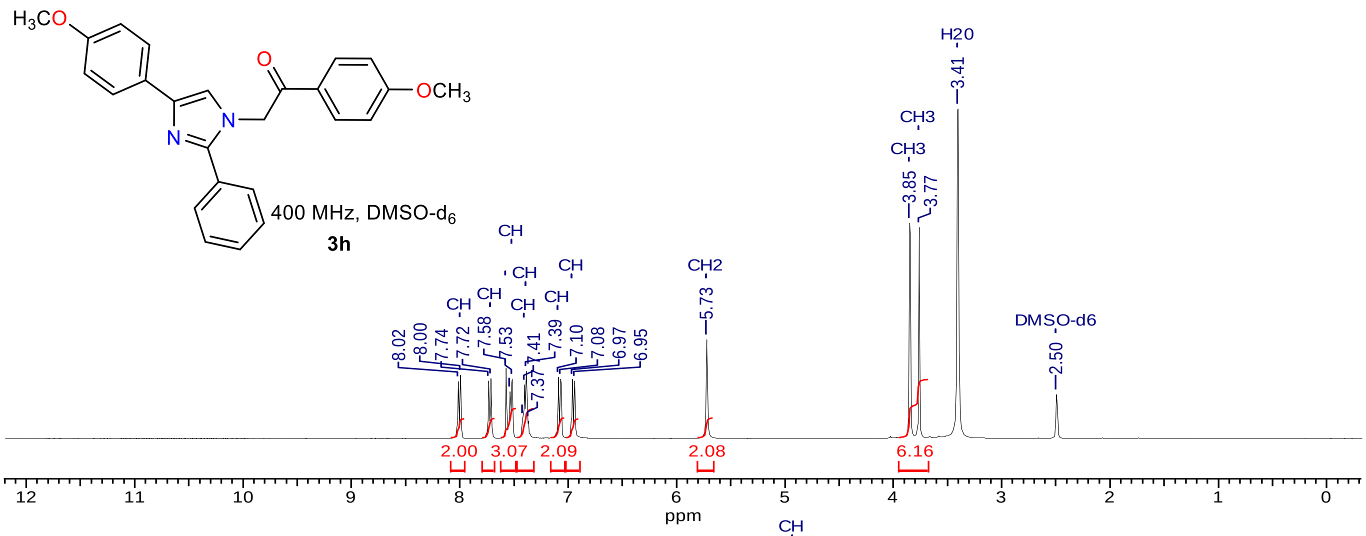

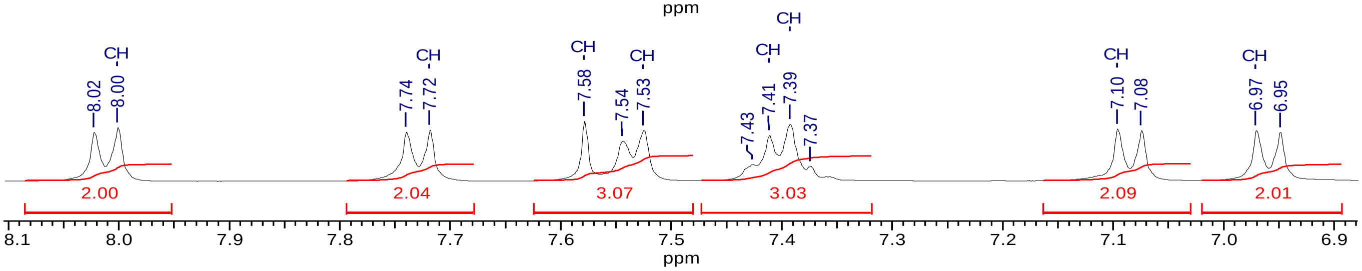

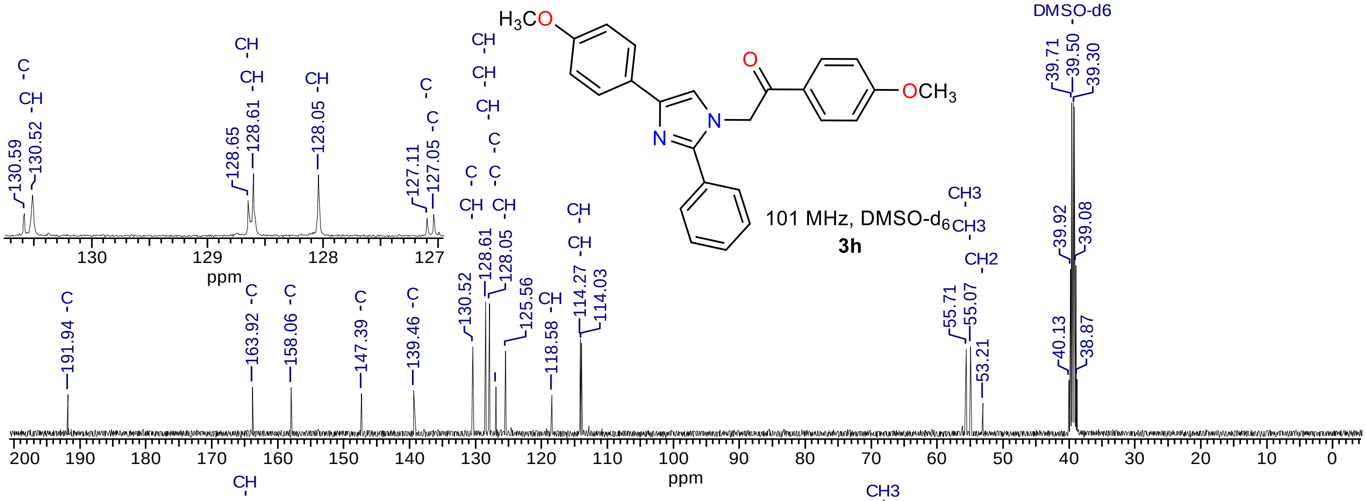

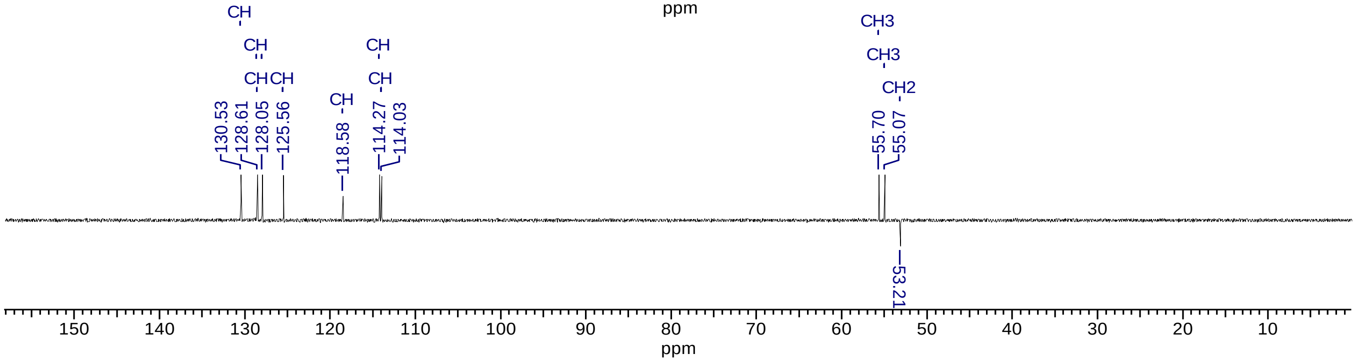

^{1}H and $^{13}C\{^{1}H\}$ NMR spectra of 1-(4-fluorophenyl)-2-(4-(4-fluorophenyl)-1*H*-imidazol-1-yl)ethan-1-one **3i**

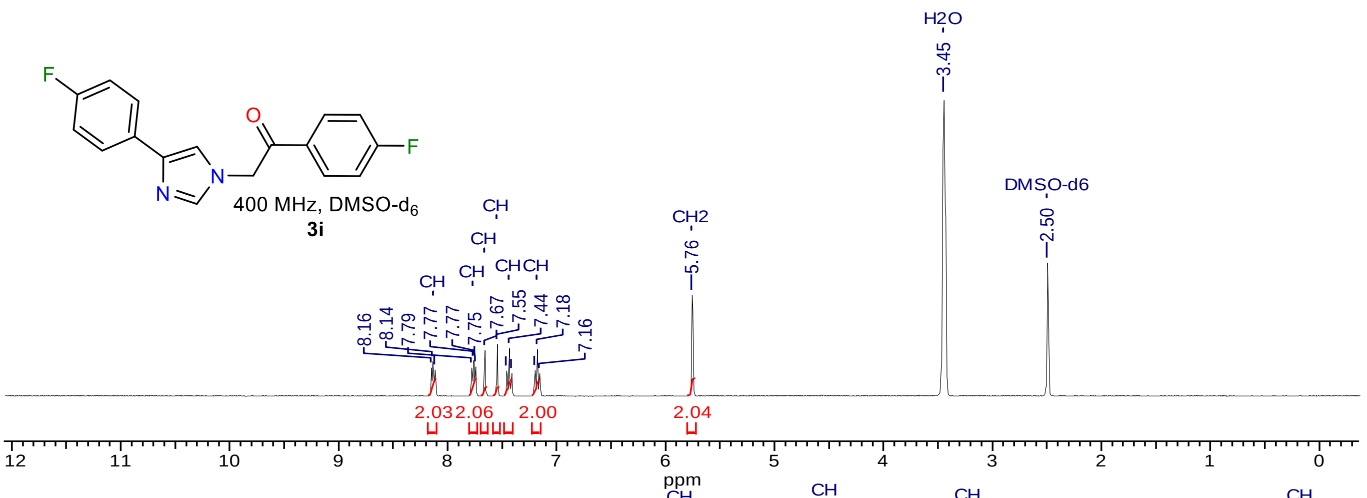

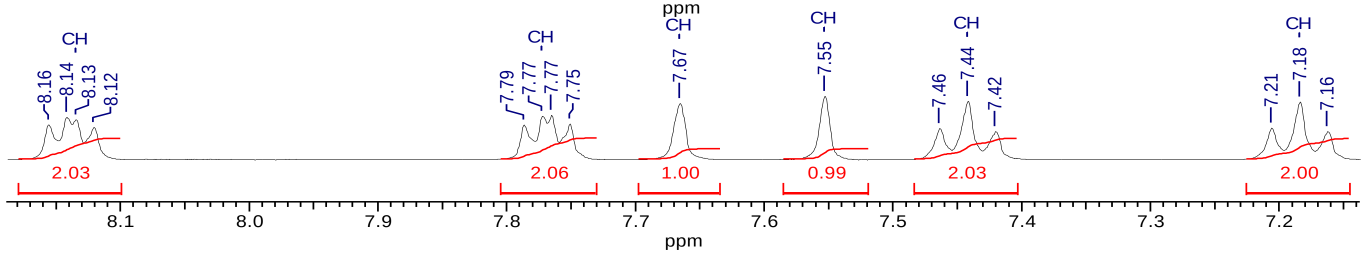

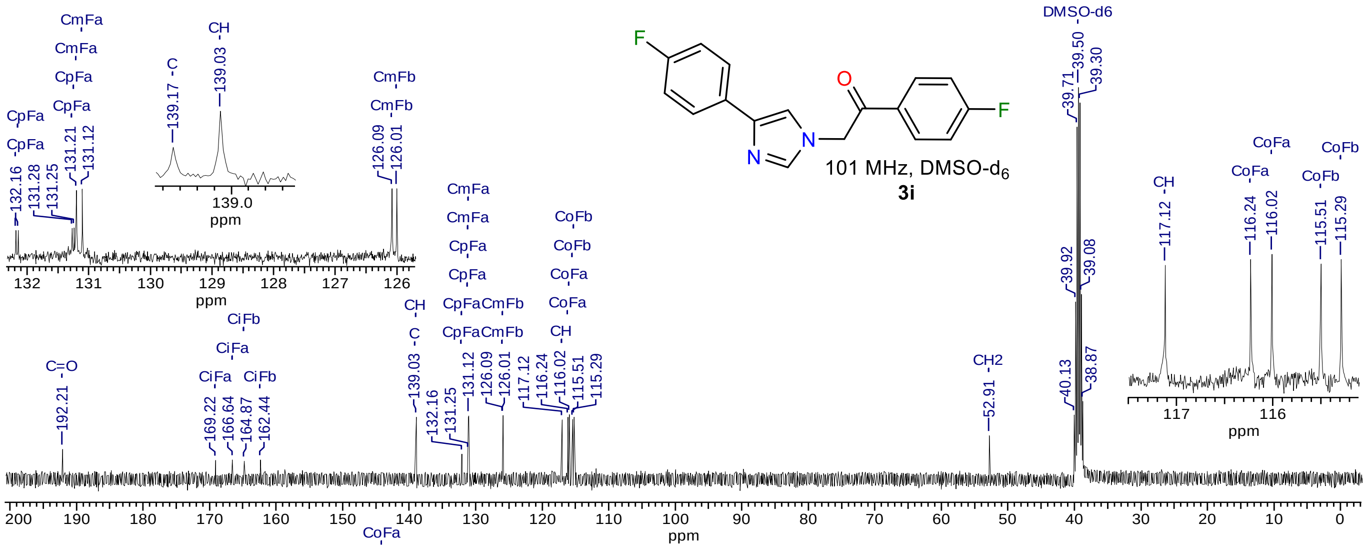

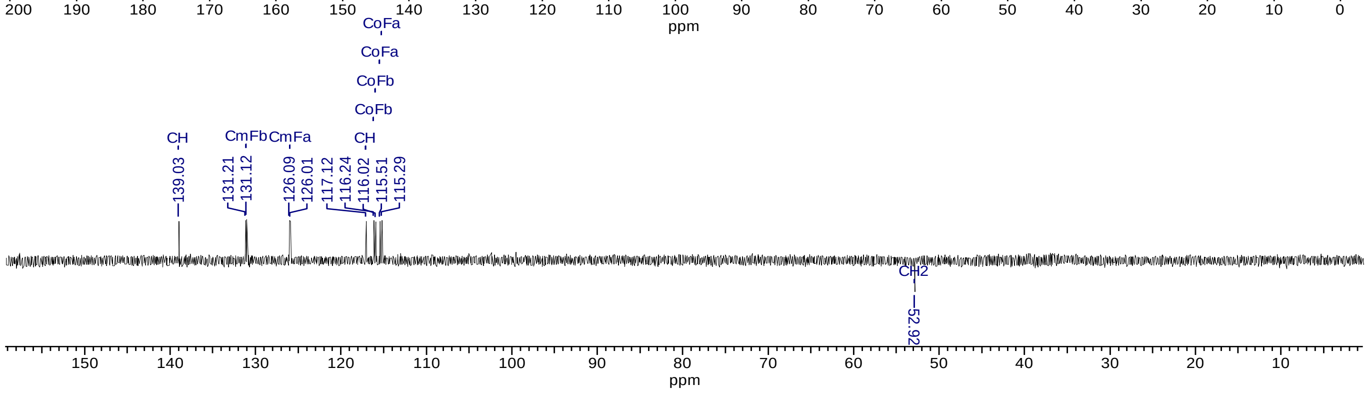

^{1}H and $^{13}C\{^{1}H\}$ NMR spectra of 1-(4-chlorophenyl)-2-(4-(4-chlorophenyl)-1*H*-imidazol-1-yl)ethan-1-one **3j**

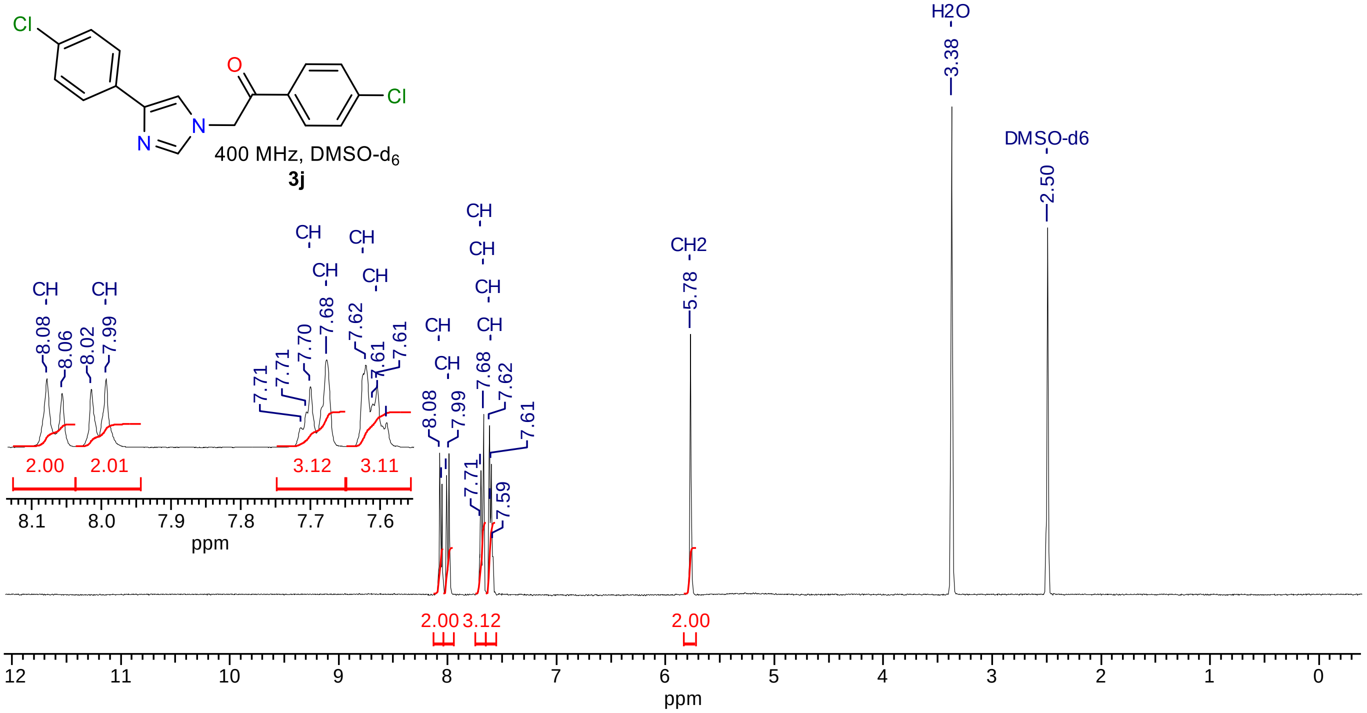

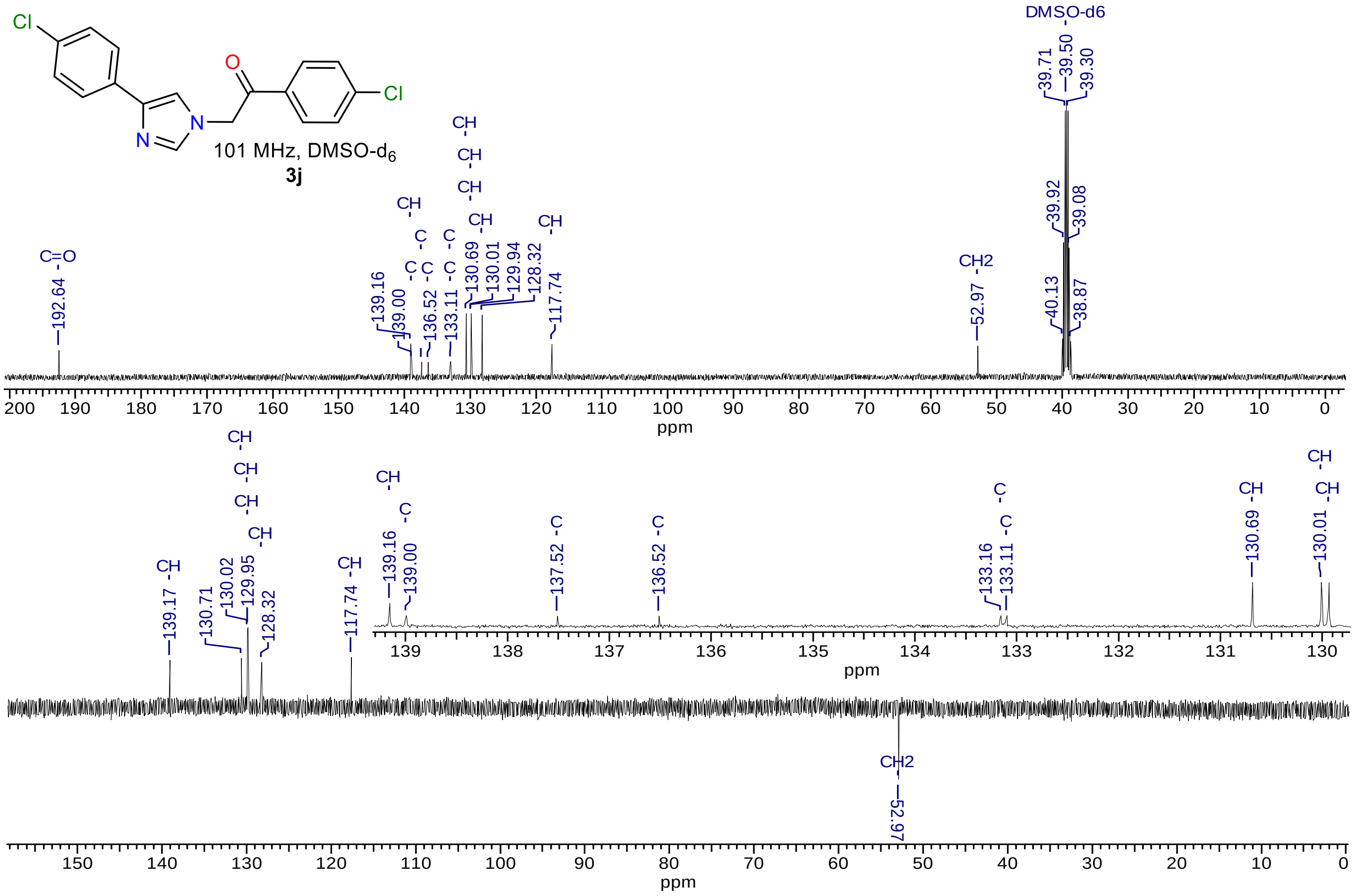

^{1}H and ^{13}C{^{1}H} NMR spectra of 1-(4-bromophenyl)-2-(4-(4-bromophenyl)-1*H*-imidazol-1-yl)ethan-1-one **3k**

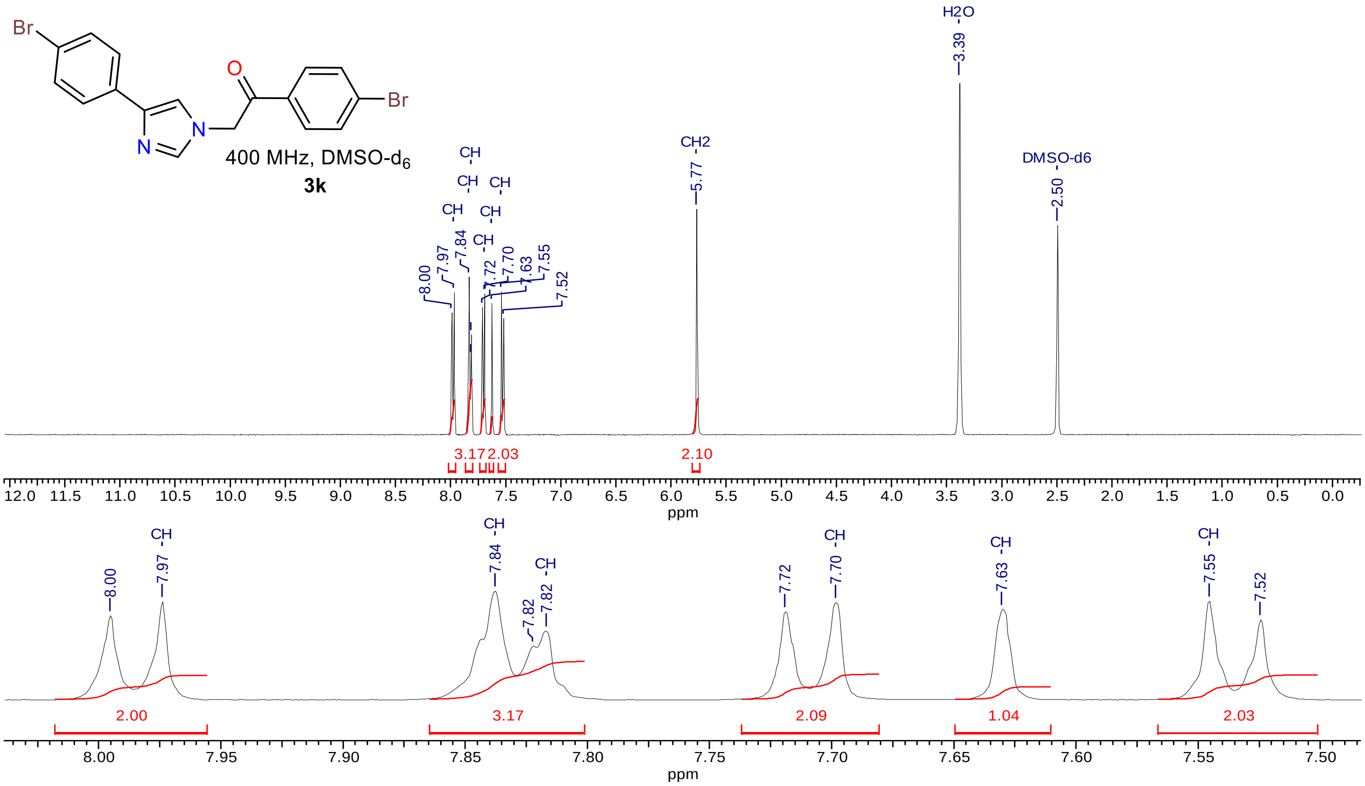

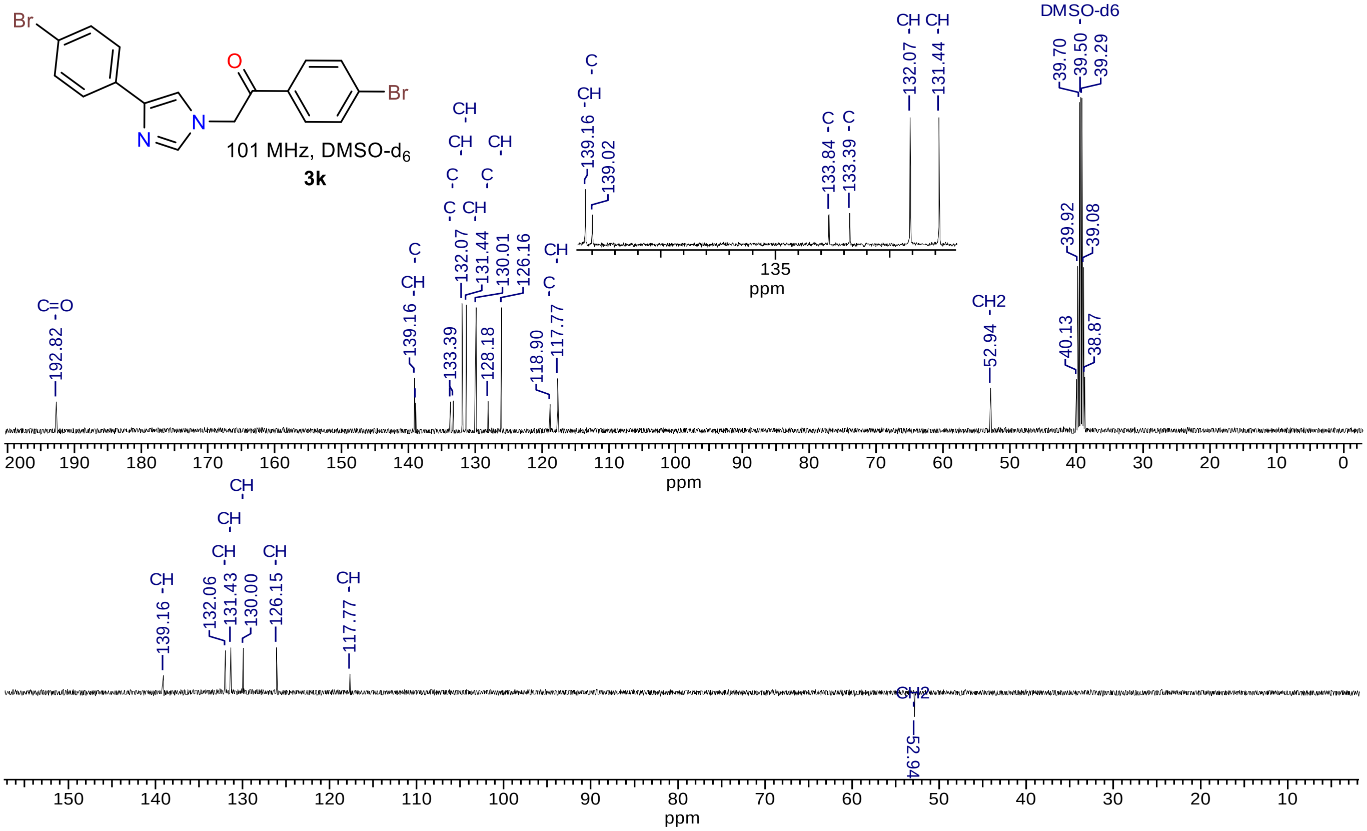

^{1}H and $^{13}C\{^{1}H\}$ NMR spectra of 1-(4-methoxyphenyl)-2-(4-(4-methoxyphenyl)-1*H*-imidazol-1-yl)ethan-1-one **3l**

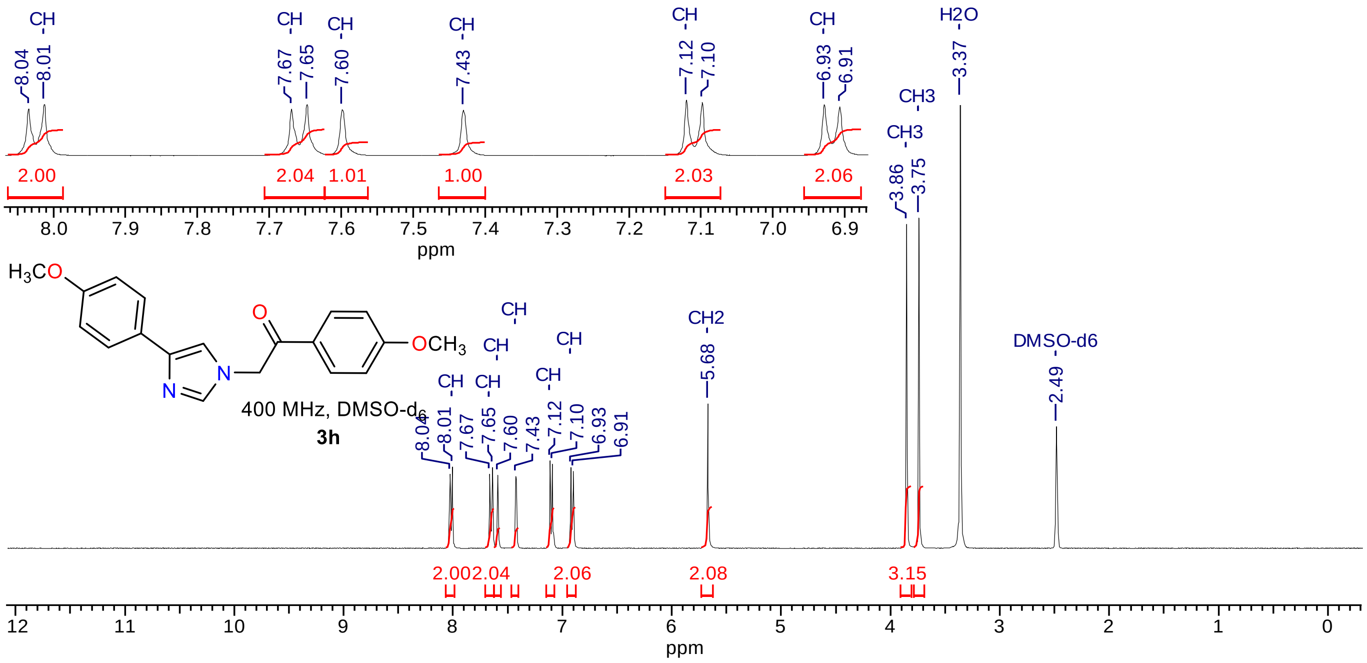

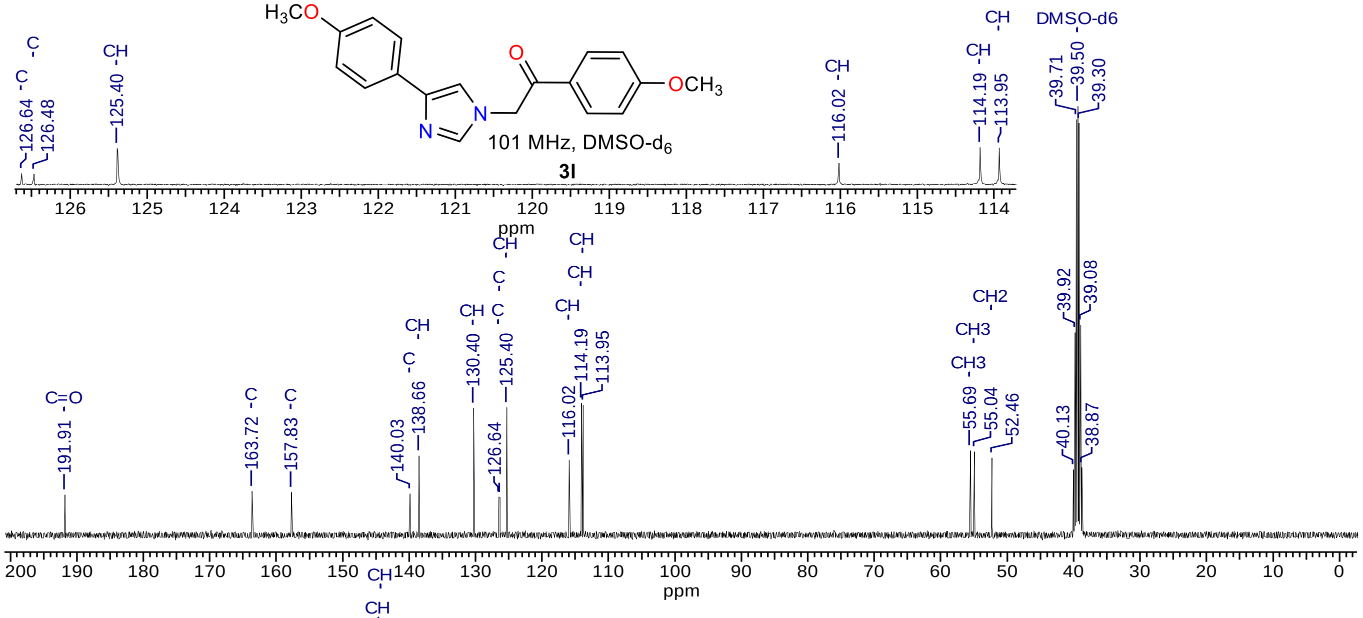

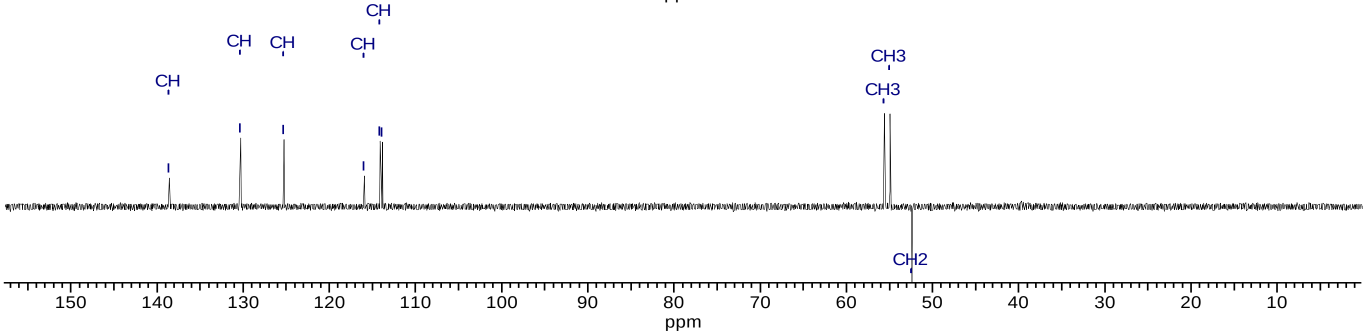

3. HRMS analysis data of substituted imidazoles 3e-l

User Spectra

Fragmentor Voltage	Collision Energy	Ionization Mode
175	0	ESI

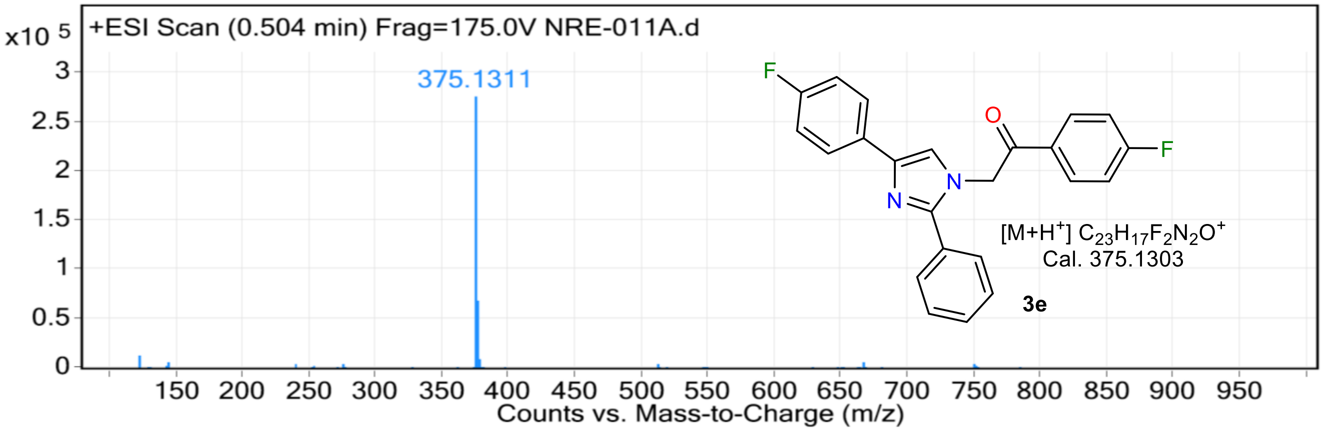

Peak List

m/z	z	Abund
375.1311	1	276064.5
375.2322		13904
375.3094		14595.4
376.1333	1	69914.2

User Spectra

Fragmentor Voltage	Collision Energy	Ionization Mode
125	0	ESI

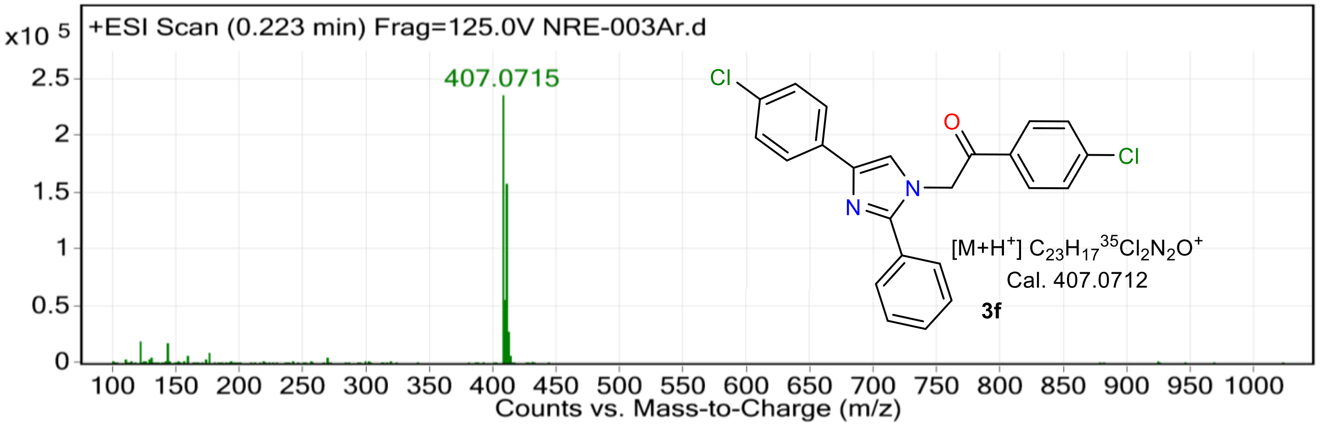

Peak List

m/z	z	Abund
121.0509		19972.5
142.1584		18343.9
407.0715	1	235651.3
408.0744	1	56768.8
409.069	1	157316.5
410.071	1	39741.6
411.0664	1	27668.3

User Spectra

Fragmentor Voltage	Collision Energy	Ionization Mode
175	0	ESI

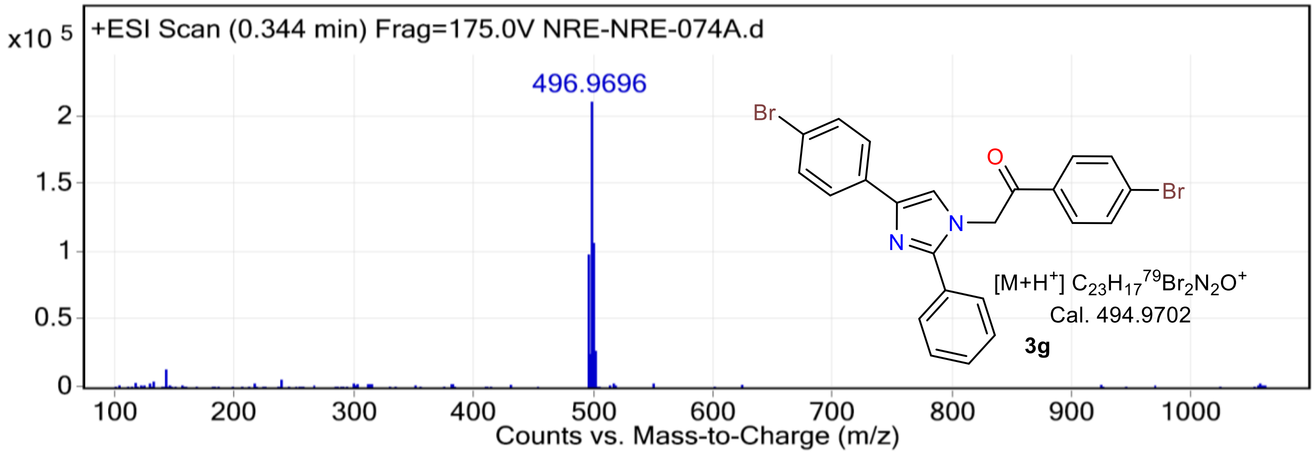

Peak List

m/z	z	Abund
142.1587		13914.6
494.9704	1	99108.8
495.9727	1	25268.8
496.9696	1	211208.2
497.9724	1	51645.8
498.9684	1	107521.3

User Spectra

Fragmentor Voltage	Collision Energy	Ionization Mode
175	0	ESI

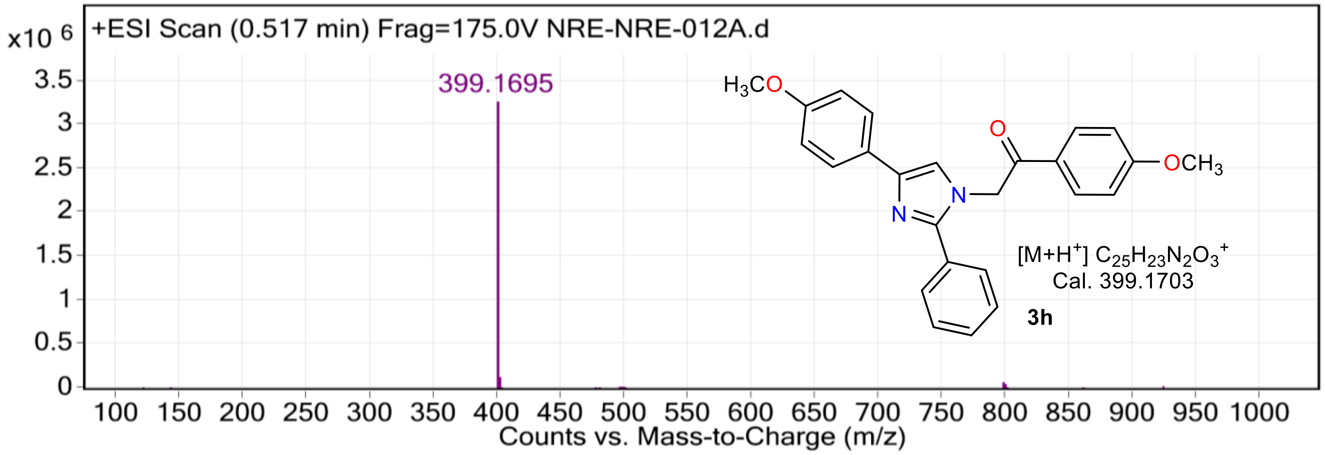

Peak List

m/z	z	Abund
399.1695	1	3261730
399.3414		194217.4
400.1738	1	929067.3

User Spectra

Fragmentor Voltage	Collision Energy	Ionization Mode
175	0	ESI

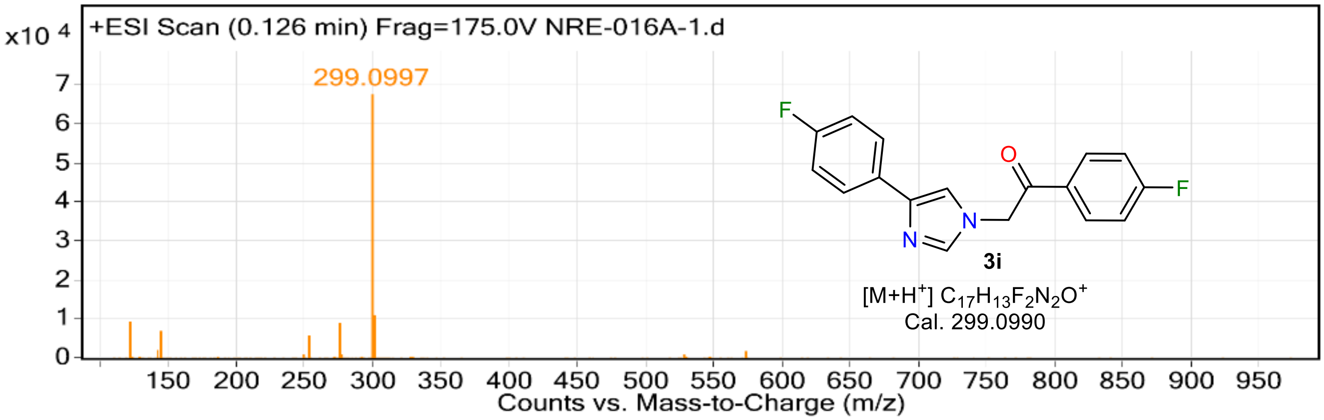

Peak List

m/z	z	Abund
122.0808		9673.3
144.0628		7156.4
253.0562		5983.3
275.0378		9396.3
299.0997	1	67768.4
299.1489		4805.1
299.1792		3496.1
299.2581		3554.1

User Spectra

Fragmentor Voltage	Collision Energy	Ionization Mode
175	0	ESI

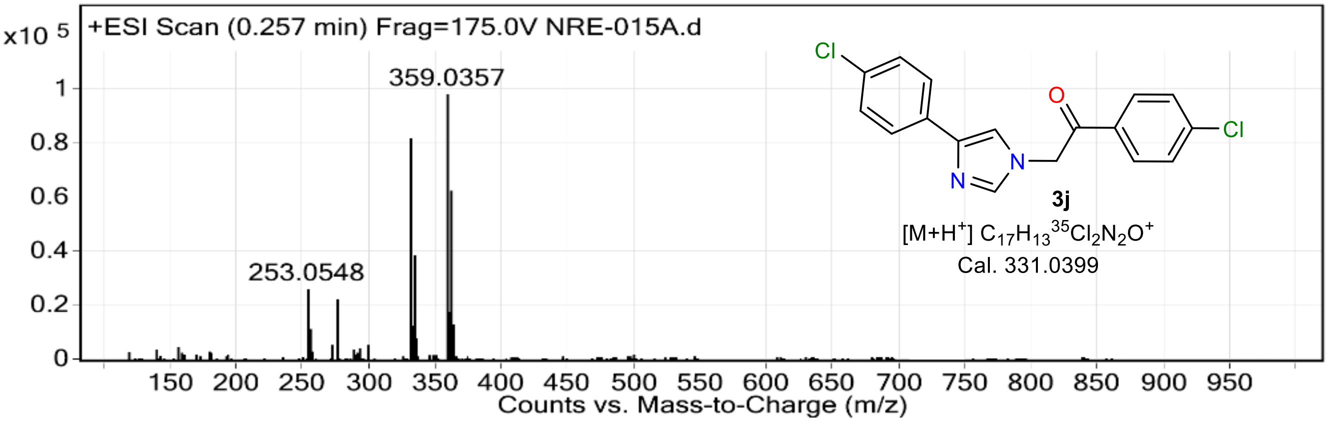

Peak List

m/z	z	Abund
253.0548		26291.7
275.038		22952.6
331.04	1	82548.6
333.0371	1	39124.6
359.0357	1	98576.6
360.0398	1	18375.8

User Spectra

Fragmentor Voltage	Collision Energy	Ionization Mode
175	0	ESI

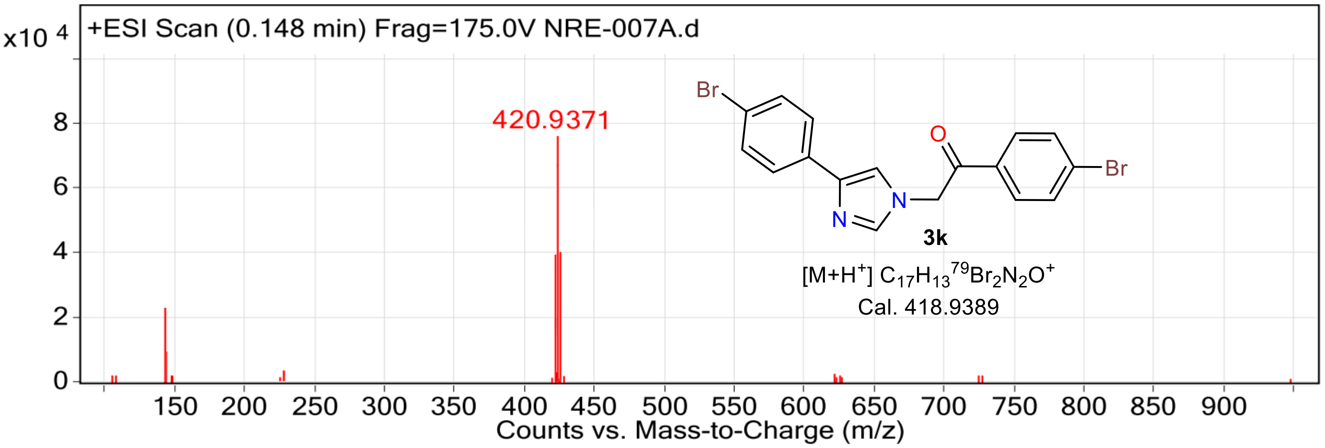

Peak List

m/z	z	Abund
142.15872		23701.2
143.1182		9139
418.93826	1	39391.2
420.9371	1	76488.7
421.93812	1	1739
422.93502	1	40228.8

Fragmentor Voltage	Collision Energy	Ionization Mode
175	0	ESI

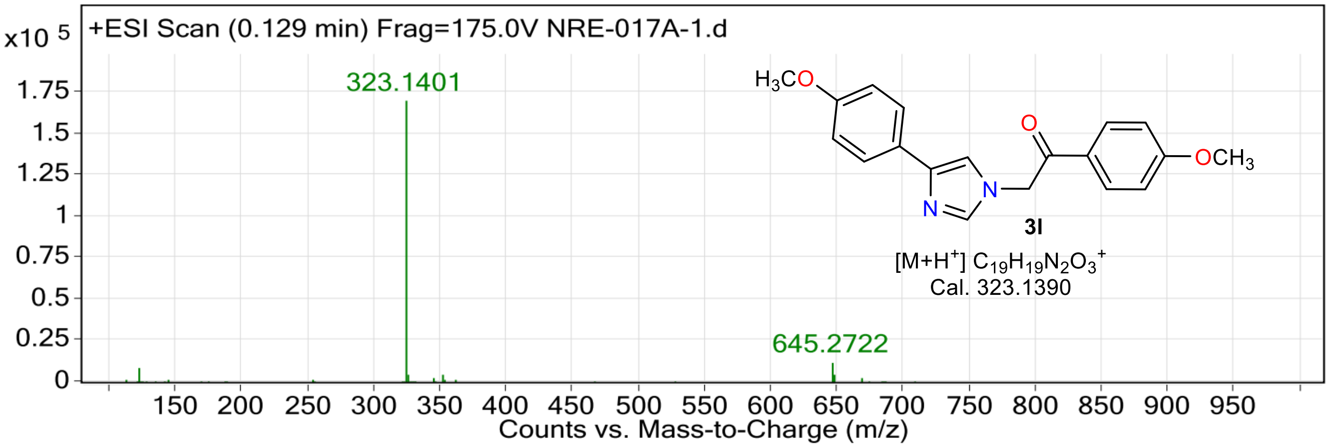

Peak List

m/z	z	Abund
122.0809		8789.4
323.1401	1	170516.4
645.2722		12274

4. Computational details

All calculations were performed using the software Gaussian 16 [28] with the M08-HX functional [29]. The basis set 6-31+G* was used for all atoms. The geometries of all critical points on the Potential Energy Surface (PES) were optimized with the gradient method available in Gaussian 16. Vibrational frequency analyses were carried out to characterize all critical points as minima or transitions states. The effect of solvent used in the reaction (acetonitrile with dielectric constant ε = 35.688) was evaluated using the SMD solvation model.

Electronic Structure Calculations. The 2 equiv of K_2CO_3 used in the experimental condition motivated us to analyze the two deprotonation possibilities of the *N*-aroylmethylamidine **I1** formed in the first step of the synthesis, which lead to two possible starting reactive species (Scheme S1). We considered the reaction pathways starting from the anion **I'1** (Scheme S1) since resulted to be 18.7 kcal/mol more stable than **I''1**.

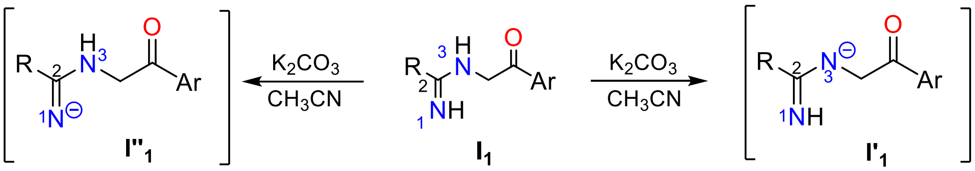

Scheme S1. Two possibilities deprotonation routes of $\mathbf{I_1}$ using K_2CO_3.

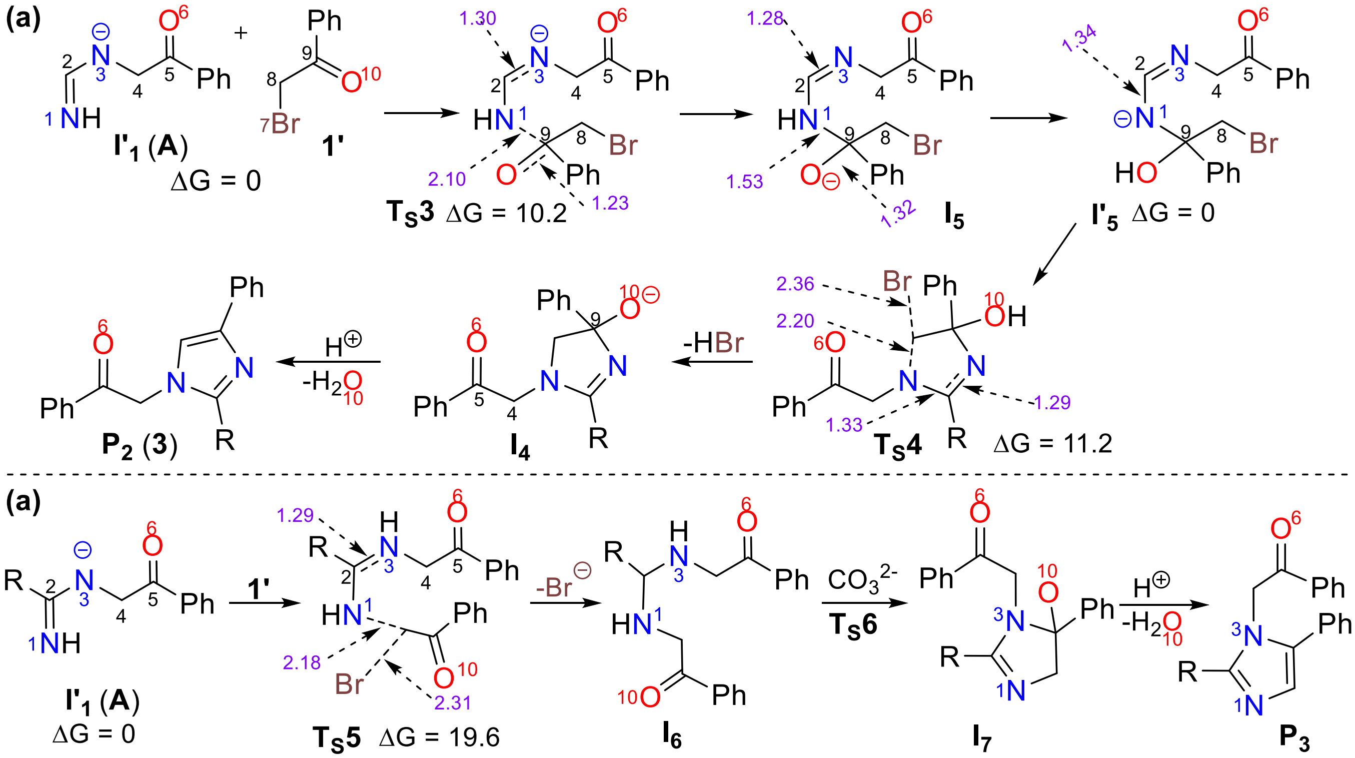

Bond lengths are in angstroms and Gibbs's free energies in kcal/mol. Compound $\mathbf{P_2}$ are like **3**.

Scheme S2. Possible pathways leading to N-substituted imidazoles $\mathbf{P_2}$ and $\mathbf{P_3}$.

As shown in Table S1, **T**s**1** and **T**s**2** have comparable stability while the energy of **T**s**5** is higher in all cases. Therefore, the possibility of obtaining the 1,5-di- or 1,2,5-tri-substituted imidazole is discarded, and the results of our calculations corroborate the observed regioselectivity in this reaction.

Table S1. Energies of key transition states in the discussed mechanism of reaction.[a]

Entry	Substituent R	ΔG (Ts1, (Kcal/mol)	ΔG (Ts2, Kcal/mol)	ΔG (Ts3, Kcal/mol)
1	H	11.7	10.8	10.2
2	CH_3	10.9	11.7	12.3
3	Ph	8.8	11.7	12.6
4	*t*-Bu	5.5	12.6	17.1

[a] ΔG = 0 was set as **I'1** and the phenacyl bromide in a non-interacting model.

When **T**s**3** is competitive (Entry 1), we analyzed the rest of the reaction profile as shown in Figure S1. The deprotonation of I5 to produce **I'**5 implies a change in PES and this prevents a direct comparison transitions states energy. Therefore, the relative energy barrier associated to **T**s**4** (11.2 kcal/mol) and the **I**4 energy (-49.0 kcal/mol) suggest that, when R= H, this alternative pathway could be competitive with path 1, anyway leading to the same product.

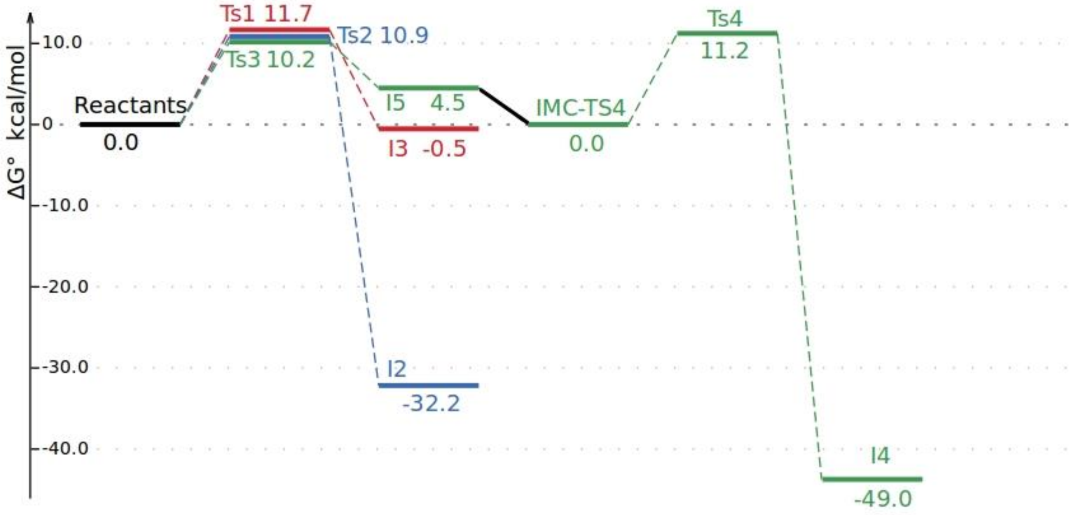

Figure S1. Reaction profile comparison between the *N*1-alkylation (→C-Br, blue), *N*-Addition (→C=O, green), and *N*3-alkylation (red) mechanisms. The strong black line indicates the change in PES.

Cartesian coordinates of all the discussed critical points.

The discussed critial points are R1 = CH_3, R2 = H , R3= Ph , R4= *t*-Bu.

First part	*Second part*	*Third part*	*Fourth part*
Br	C -1.609374 1.367121 2.355888	C -0.281113 -2.113127 -2.758448	H -0.884113 -0.292941 -
1	C -0.110453 -0.989258 2.262522	C -0.336571 -1.346627 -3.931163	1.556387
-2571.996861	H -0.523636 -0.989218 0.141533	C 0.95412 -2.317166 -2.131038	N -2.339527 -1.460427 -
Br 0.36918100	C -1.067665 0.841243 3.528197	C 0.833763 -0.832553 -4.487605	0.625622
0.18860400 0.00000000	H -2.19641 2.287883 2.377838	H -1.28947 -1.187941 -4.437623	C -3.579069 -1.394783 -
R1_I1_p	C -0.315909 -0.338352 3.481574	C 2.126098 -1.79969 -2.684401	0.078786
24	H 0.47962 -1.906954 2.222523	H 0.986605 -2.891917 -1.203298	C -3.987966 -2.533607 0.885826
Energy=-572.195704	H -1.230018 1.348979 4.480723	C 2.066821 -1.055934 -3.865204	N -4.371822 -0.372251 -
C -0.06104 -0.800018 -0.337968	H 0.109939 -0.749939 4.398803	H 0.784611 -0.253087 -5.411179	0.282838
O -0.188014 -2.004993 -	C -2.297593 0.397719 -1.305907	H 3.083915 -1.967396 -2.188619	H -5.235235 -0.475639 0.254253
0.192236	H -2.988366 0.986647 -1.942507	H 2.980212 -0.647123 -4.30154	C -2.730597 -2.796619 -
C -0.349852 -0.158581 -1.663626	H -1.348178 0.303783 -1.872048	Br -2.362367 -4.26185 -4.433919	2.327597
C -0.303491 1.231363 -1.840782	N -2.794674 -0.926351 -	C -4.980599 -2.107514 -0.238292	H -3.390446 -3.37344 -1.690195
C -0.681782 -0.982805 -2.747922	1.012382	C -6.373271 -2.203828 -0.346218	H -3.113661 -1.889745 -
C -0.588034 1.787214 -3.089056	C -3.901851 -0.935728 -0.292384	C -4.251648 -3.170636 0.310172	2.787185
H -0.051895 1.89143 -1.008769	N -4.542916 0.118178 0.190703	C -7.031854 -3.357446 0.081877	C -1.282049 -3.179451 -
C -0.961437 -0.427074 -3.994632	H -5.361407 -0.196555 0.71191	H -6.941657 -1.376898 -	2.321652
H -0.71634 -2.063726 -2.600677	C -2.521435 -3.636291 -1.801998	0.778807	O -0.896959 -4.134626 -1.67028
C -0.915099 0.9602 -4.166269	H -3.026817 -3.892772 -	C -4.914387 -4.316682 0.751511	C -0.322716 -2.369654 -
H -0.553266 2.869852 -3.220445	0.867252	H -3.164064 -3.098506 0.382044	3.145835
H -1.215981 -1.074517 -	H -3.159935 -3.027431 -	C -6.303623 -4.414256 0.634315	C -0.742516 -1.423246 -
4.835412	2.440636	H -8.116112 -3.431342 -	4.091623
H -1.13483 1.397409 -5.14208	C -1.154286 -3.056042 -1.507901	0.017775	C 1.046456 -2.575508 -2.938486
C 0.365842 0.088417 0.810621	O -0.585441 -3.364123 -	H -4.342729 -5.137848 1.187712	C 0.200093 -0.690886 -4.812814
H 1.193245 0.734591 0.471607	0.475085	H -6.819152 -5.315023 0.972433	H -1.804146 -1.258783 -
H -0.473382 0.783673 1.026672	C -0.484757 -2.199729 -2.538053	R3_P1	4.283842
N 0.747056 -0.683709 1.960838	C -1.087635 -1.883002 -3.762888	29	C 1.989317 -1.837249 -3.652816
C 0.749172 -0.003235 3.241148	C 0.776799 -1.672173 -2.235943	Energy=-688.010317	H 1.362745 -3.314528 -2.200111
C 0.811178 -1.075633 4.344418	C -0.44348 -1.03307 -4.661774	C 0.163895 0.171853 -0.190596	C 1.56567 -0.896456 -4.595486
H 1.674717 -1.747401 4.207462	H -2.068323 -2.283414 -	C 1.96304 1.451454 -0.126313	H -0.13264 0.040763 -5.55096
H 0.892626 -0.595659 5.327778	4.025422	C 0.820119 2.229145 -0.105728	H 3.054534 -1.997424 -3.477817
H -0.091878 -1.709658 4.341147	C 1.421544 -0.821388 -3.133675	N -0.284344 1.414981 -0.146262	H 2.300419 -0.319562 -5.160395
N 0.742734 1.242732 3.393631	H 1.244615 -1.928914 -1.283178	H 3.019649 1.69614 -0.107852	Br -3.187024 -4.097807 -
H 0.296741 -1.597146 1.972762	C 0.809485 -0.498305 -4.347771	N 1.524326 0.160463 -0.179738	4.177593
R1_I1	H -0.922141 -0.786072 -	C 0.704907 3.697275 -0.04892	C -2.891333 -3.589959 1.057909
24	5.610828	C -0.559497 4.30518 -0.017912	H -3.245882 -4.368899 1.752749
Energy=-572.225497	H 2.400685 -0.408006 -2.885881	C 1.847135 4.514624 -0.024917	H -2.608429 -4.076195 0.114477
C -0.143572 -0.934589 -0.367061	H 1.308356 0.171262 -5.050949	C -0.678858 5.69473 0.036379	H -1.977645 -3.1457 1.483151
O -0.215514 -2.149984 -	Br -2.210859 -5.334039 -	H -1.452973 3.678818 -0.035644	C -4.231095 -1.89105 2.267998
0.323983	2.750384	C 1.725637 5.902604 0.02955	H -5.07534 -1.184579 2.256912
C -0.40695 -0.225494 -1.675649	H -4.295418 -1.952017 -	H 2.841767 4.06339 -0.049528	H -4.458843 -2.673427 3.009852
C -0.367945 1.171017 -1.798467	0.091461	C 0.461949 6.500735 0.0603	H -3.335108 -1.346932 2.611743
C -0.701777 -1.002856 -2.804503	R2_IMC2_TS1	H -1.671009 6.149866 0.060069	C -5.287499 -3.211764 0.420142
C -0.621513 1.775881 -3.031274	38	H 2.624455 6.521967 0.047242	H -5.158249 -3.740284 -
H -0.141466 1.800012 -0.936073	Energy=-3488.855411	H 0.368285 7.587209 0.102716	0.537498
C -0.954188 -0.399695 -4.035332	C -3.611657 0.55105 0.028572	H 2.128537 -0.655662 -0.210493	H -5.610806 -3.954226 1.167113
H -0.730343 -2.089161 -	O -4.51404 1.520625 0.062603	C -0.685596 -1.030836 -0.244815	H -6.106065 -2.486137 0.295534
2.702318	C -2.482729 0.70093 1.076978	C -0.146731 -2.325891 -0.254916	R4_Ts5.xyz
C -0.914522 0.993343 -4.150584	C -1.548222 -0.319101 1.313531	C -2.079841 -0.872139 -0.286601	50
H -0.589783 2.863375 -3.116844	C -2.354712 1.898956 1.783501	C -0.987707 -3.438365 -0.307219	Energy=-3645.944203

H -1.182245 -1.015079 -4.907483
H -1.112411 1.46886 -5.113109
C 0.198758 -0.077174 0.835403
H 1.063859 0.559404 0.541801
H -0.630878 0.656402 0.950352
N 0.443307 -0.825758 2.031464
C 0.733796 -0.06454 3.077336
C 1.004174 -0.812497 4.377531
H 2.023297 -0.606833 4.740219
H 0.30806 -0.487359 5.166222
H 0.895378 -1.89667 4.242162
N 0.803177 1.266092 3.060642
H 1.042719 1.596455 3.996943
R1_I3_O_neg
24
Energy=-572.229919
C -0.830487 0.162904 -0.154072
C 0.729448 0.35373 -0.293812
C -0.101166 2.359405 0.086694
N -1.190681 1.632741 -0.335451
H 1.247231 -0.378768 0.344031
N 1.024035 1.727272 0.122878
C -0.260443 3.806255 0.445077
H -0.657168 4.377474 -0.406611
H -0.972149 3.919708 1.276452
H 0.7042 4.234753 0.742312
H 1.078198 0.208393 -1.332226
C -1.394785 -0.61771 -1.367053
C -2.030597 -1.84298 -1.148634
C -1.267026 -0.160678 -2.688127
C -2.530354 -2.598285 -2.215885
H -2.12693 -2.1831 -0.115541
C -1.764581 -0.906917 -3.758376
H -0.777515 0.798032 -2.880613
C -2.39939 -2.132704 -3.525826
H -3.024443 -3.553567 -2.023171
H -1.66002 -0.531588 -4.778953
H -2.78901 -2.717634 -4.361509
O -1.221396 -0.319525 1.018192
H -2.087696 1.888989 0.073585
R1_I5
40
Energy=-956.199688
C 2.405743 0.09134 -0.065936
O 3.614732 -0.036746 -0.060026
C 1.768295 1.44729 -0.071841
C 0.376661 1.614321 -0.040617
C 2.601323 2.575061 -0.104324
C -0.172786 2.897635 -0.040389
H -0.290004 0.750818 -0.01519
C 2.051376 3.854497 -0.10594
H 3.683341 2.434184 -0.128452
C 0.662128 4.016703 -0.073528
H -1.256292 3.023047 -0.014797
C -0.510083 -0.142227 2.229717
H -1.64515 -1.268172 0.77665
C -1.317196 2.083435 2.705361
H -3.092707 2.680986 1.592913
C -0.389317 1.064376 2.930073
H 0.207419 -0.948288 2.402767
H -1.232673 3.027268 3.249353
H 0.421752 1.204781 3.647564
C -2.988122 0.277945 -1.399458
H -3.08767 1.185078 -2.015444
H -1.914054 0.020746 -1.346521
N -3.72311 -0.84513 -1.993146
C -4.39352 -1.369946 -1.02336
N -4.22621 -0.834385 0.217428
H -5.00699 -0.882023 0.867538
C -2.766262 -3.721832 -2.536699
H -3.609247 -4.133967 -1.976
H -3.111549 -3.02349 -3.301036
C -1.735314 -3.153182 -1.580928
O -1.7823 -3.464041 -0.404282
C -0.663318 -2.257169 -2.109799
C -0.687902 -1.751592 -3.416935
C 0.34896 -1.850414 -1.229927
C 0.283413 -0.840881 -3.831558
H -1.472042 -2.043164 -4.117691
C 1.32497 -0.947812 -1.646594
H 0.360023 -2.248229 -0.213105
C 1.290337 -0.440115 -2.948967
H 0.254252 -0.442905 -4.846986
H 2.110108 -0.635726 -0.956121
H 2.04872 0.273375 -3.276688
Br -1.915668 -5.225296 -3.461161
H -5.04986 -2.235078 -1.163905
R2_IMC2_TS2
38
Energy=-3488.913293
C -2.912441 1.362813 -0.417073
O -3.427448 2.465029 -0.451617
C -1.854741 1.042611 0.593406
C -0.764186 0.23365 0.259272
C -1.942449 1.603721 1.873453
C 0.237933 -0.013275 1.19969
H -0.678183 -0.176989 -0.748498
C -0.951189 1.342598 2.817911
H -2.800144 2.232268 2.123073
C 0.141698 0.535999 2.480272
H 1.095449 -0.633363 0.929906
H -1.026668 1.769134 3.819586
H 0.920295 0.336826 3.218914
C -3.26762 0.326451 -1.484144
H -4.219118 0.651737 -1.939059
H -2.486116 0.373616 -2.260731
H 0.932532 -2.485282 -0.220326
C -2.915869 -1.985714 -0.338353
H -2.501898 0.134052 -0.278649
C -2.373882 -3.274457 -0.34924
H -0.553464 -4.439607 -0.314042
H -3.997879 -1.846199 -0.370835
H -3.028923 -4.146343 -0.389176
R3_P2
44
Energy=-1071.496117
C -1.612315 2.619226 0.080329
C -0.367778 3.857921 1.411102
C -1.244994 4.653822 0.704297
N -2.004105 3.864212 -0.123341
H 0.398285 4.081218 2.147339
N -0.607084 2.570836 1.007427
C -1.414515 6.116445 0.764448
C -2.391163 6.748074 -0.020079
C -0.606454 6.903816 1.601277
C -2.554436 8.133457 0.029782
C -0.771587 8.287295 1.649986
H 0.159955 6.432047 2.220549
C -1.746444 8.909873 0.864057
H -3.319112 8.608675 -0.58774
H -0.13452 8.883733 2.305892
H -1.875045 9.992907 0.903052
C 0.183317 1.432594 1.450084
H 1.07691 1.836058 1.949881
H -0.364262 0.837314 2.19231
C 0.670684 0.472054 0.360998
O 0.86551 -0.685478 0.680601
C 0.917873 0.940915 -1.036218
C 1.228692 2.273188 -1.342902
C 0.795826 -0.004509 -2.063861
C 1.408103 2.653477 -2.673176
H 1.346359 3.015954 -0.550136
C 0.961081 0.382298 -3.392196
H 0.547353 -1.03758 -1.810317
C 1.262631 1.713273 -3.697454
H 1.657464 3.688765 -2.911102
H 0.848101 -0.351621 -4.191913
H 1.388252 2.018856 -4.737829
H -3.024966 6.144086 -0.671672
C -2.116157 1.455392 -0.674363
C -2.331177 0.20639 -0.075549
C -2.354311 1.611678 -2.049326
C -2.749454 -0.878315 -0.849671
H -2.190509 0.076191 0.999866
C -2.780759 0.529332 -2.815974
H -2.180752 2.585165 -2.513417
C -2.968864 -0.722249 -2.219765
H -2.911623 -1.847698 -0.375236
C -0.373461 -2.855274 1.98743
O -1.484743 -3.221587 2.322031
C 0.726691 -3.867866 1.793286
C 2.025522 -3.502245 1.410949
C 0.431696 -5.220542 2.014765
C 3.011695 -4.478165 1.253264
H 2.281508 -2.456736 1.232472
C 1.4157 -6.194372 1.856327
H -0.581086 -5.497228 2.313137
C 2.709156 -5.823514 1.474791
H 4.019923 -4.185245 0.955667
H 1.176336 -7.244825 2.030536
H 3.482013 -6.584501 1.351005
C -0.031187 -1.386161 1.753519
H 0.837962 -1.157017 2.393221
H 0.360403 -1.3333 0.719294
N -1.15209 -0.502537 1.860266
C -1.449924 0.19742 2.920904
N -2.518827 1.035919 2.810222
H -2.768501 1.408055 3.722515
C -1.44067 2.539225 1.036876
C -2.32137 2.256453 -0.145415
C -3.675185 2.613819 -0.18984
C -1.754944 1.574416 -1.228055
C -4.447631 2.290214 -1.30599
H -4.137604 3.151338 0.639181
C -2.525073 1.25115 -2.344521
H -0.701513 1.292528 -1.177902
C -3.874495 1.612766 -2.386077
H -5.501435 2.572598 -1.334785
H -2.074395 0.716319 -3.18242
H -4.482162 1.362064 -3.257662
C -2.045359 3.121811 2.263887
H -1.395759 3.211039 3.130998
H -3.1144 3.215353 2.410359
Br -1.827729 5.318262 1.602509
O -0.242921 2.312276 0.998314
C -0.635102 0.199082 4.256895
C 0.693235 0.949583 4.034027
H 0.500975 1.982649 3.70602
H 1.258789 0.989726 4.979787
H 1.339699 0.48389 3.278061
C -1.374935 0.957973 5.375955
H -0.770219 0.915518 6.295048
H -1.519904 2.023823 5.138928
H -2.355325 0.510886 5.606189
C -0.380388 -1.221771 4.800777
H -1.305791 -1.81751 4.808704
H 0.380037 -1.783974 4.243612
H -0.018025 -1.151887 5.838937
Ts3_r4
50
Energy=-3645.959020
C -0.259628 -2.8897 2.159215
O -1.456852 -3.106937 2.190798
C 0.712633 -4.04291 2.132289

H 2.704129 4.728568 -0.131813
H 0.230056 5.019133 -0.074256
C 1.486414 -1.13219 -0.064767
H 0.856273 -1.090007 0.836939
H 0.79103 -1.070913 -0.92011
N 2.20664 -2.381227 -0.075407
C 2.674503 -2.890925 -1.258242
C 3.240594 -4.289432 -1.217807
H 4.155605 -4.331919 -0.608959
H 2.523399 -5.011306 -0.800357
H 3.491453 -4.59267 -2.239746
N 2.672432 -2.264204 -2.388556
H 2.301126 -1.315425 -2.314357
C 2.196936 -3.143084 1.150854
H 3.062197 -3.818031 1.204091
H 2.300589 -2.453275 2.00333
C 0.910314 -3.953758 1.325433
O 0.051133 -3.936812 0.463874
C 0.742412 -4.75527 2.578372
C 1.735371 -4.799502 3.567091
C -0.446017 -5.478456 2.755575
C 1.538737 -5.561173 4.720227
H 2.667728 -4.244961 3.448738
C -0.64121 -6.23617 3.907542
H -1.212691 -5.436837 1.979855
C 0.352578 -6.278268 4.891432
H 2.314538 -5.594249 5.486708
H -1.568155 -6.796115 4.041421
H 0.200768 -6.872262 5.794546
R1_IMC1
41
Energy=-3528.140488
C -1.965013 1.244064 -0.161523
O -2.026686 2.457156 -0.293078
C -1.407761 0.667958 1.109917
C -0.725421 -0.554903 1.115296
C -1.522984 1.40351 2.296839
C -0.160397 -1.034723 2.299661
H -0.643113 -1.134071 0.194345
C -0.975466 0.915356 3.482566
H -2.053235 2.358133 2.280131
C -0.289696 -0.304493 3.483716
H 0.37898 -1.983887 2.297553
H -1.078749 1.485719 4.407554
H 0.14389 -0.685642 4.410411
C -2.322366 0.304647 -1.31317
H -2.986493 0.905133 -1.968365
H -1.377813 0.154215 -1.87422
N -2.873725 -0.983421 -0.968435
C -3.99699 -0.899072 -0.264408
C -4.627788 -2.237687 0.104431
H -4.973456 -2.769408 -0.795131
H -3.894164 -2.884343 0.60849
H -5.489169 -2.107732 0.774804
N -3.347595 -1.041205 -1.007763
C -4.137136 -1.303755 0.066058
N -4.680042 -0.367027 0.758497
H -5.211386 -0.766999 1.531843
C -2.994669 -2.136592 -1.912809
H -3.736619 -2.939893 -1.829297
H -3.016293 -1.781244 -2.947264
C -1.640583 -2.730257 -1.508969
O -1.599508 -3.475937 -0.544364
C -0.374234 -2.226904 -2.139943
C -0.369294 -1.411748 -3.279755
C 0.83052 -2.4733 -1.469955
C 0.828135 -0.877645 -3.755927
H -1.29447 -1.21638 -3.822802
C 2.030606 -1.94961 -1.950366
H 0.814739 -3.083752 -0.564868
C 2.030361 -1.146086 -3.094212
H 0.824069 -0.252241 -4.650465
H 2.964853 -2.156732 -1.425023
H 2.966021 -0.727557 -3.469983
Br -2.164008 -4.437766 -3.984444
H -4.246075 -2.376028 0.281015
R2_P1_
19
Energy=-457.068664
C 0.223703 0.147709 -0.083256
C 1.908048 1.458962 -0.26635
C 0.80866 2.278857 -0.093267
N -0.250836 1.409772 0.01693
H -1.222958 1.655267 0.179965
H 2.943311 1.757118 -0.408889
N 1.531551 0.141135 -0.258827
C 0.683753 3.741189 -0.038518
C -0.555013 4.381316 -0.201788
C 1.828901 4.528165 0.172945
C -0.645012 5.773351 -0.151267
H -1.460633 3.798751 -0.384319
C 1.736339 5.917928 0.213377
H 2.79824 4.04601 0.315878
C 0.498256 6.548142 0.054287
H -1.616497 6.253745 -0.280731
H 2.636443 6.512788 0.378603
H 0.426072 7.636311 0.090862
H -0.421855 -0.722614 -0.019747
R2_P2
34
Energy=-840.558512
C -1.818471 2.517157 0.165346
C -0.351824 3.793359 1.185299
C -1.348864 4.564767 0.621378
H -2.953984 0.658007 -3.88598
H -3.292502 -1.572801 -2.822246
R3_P3
44
Energy=-1071.487837
C -0.165846 -0.12434 -0.295859
C 1.557796 1.093786 -0.691637
C 0.57207 1.963872 -0.281913
N -0.53055 1.167025 -0.027263
H 2.576221 1.343281 -0.979218
N 1.088939 -0.189139 -0.709198
C 0.645267 3.416318 -0.039138
C -0.354264 4.307254 -0.458753
C 1.758083 3.919264 0.656726
C -0.256304 5.667859 -0.159719
H -1.207773 3.945385 -1.035514
C 1.861465 5.280561 0.938147
H 2.531679 3.227613 0.9992
C 0.847229 6.158024 0.542098
H -1.044493 6.348538 -0.486449
H 2.728003 5.655499 1.486009
H 0.920073 7.221748 0.775245
C -1.853313 1.621007 0.367329
H -2.46381 0.741105 0.609414
H -2.350669 2.121817 -0.474281
C -1.92933 2.574813 1.562895
O -2.924358 3.270143 1.650783
C -0.838331 2.66359 2.581025
C 0.01867 1.595015 2.878527
C -0.671296 3.894941 3.230874
C 1.038375 1.764317 3.815734
H -0.110187 0.624381 2.395297
C 0.35909 4.065719 4.15287
H -1.342477 4.721329 2.986618
C 1.217544 3.00045 4.442827
H 1.698838 0.928681 4.052633
H 0.498251 5.031127 4.642317
H 2.027159 3.132867 5.162922
C -1.035555 -1.313756 -0.153212
C -1.077962 -2.239919 -1.205808
C -1.757042 -1.577914 1.02066
C -1.841909 -3.401538 -1.093411
H -0.508754 -2.040638 -2.115908
C -2.524946 -2.738578 1.128225
H -1.703579 -0.887516 1.866173
C -2.571711 -3.651163 0.07184
H -1.869975 -4.112498 -1.921002
H -3.07965 -2.934554 2.047367
H -3.171331 -4.558868 0.158927
R3_pHCOBr
17
Energy=-2955.922823
C -3.443975 1.94489 0.000524
C 2.100712 -3.854786 2.060901
C 0.199658 -5.346544 2.188608
C 2.958683 -4.956422 2.04635
H 2.526855 -2.851436 2.014821
C 1.056008 -6.445889 2.174529
H -0.881515 -5.485172 2.244906
C 2.439126 -6.251676 2.102781
H 4.03737 -4.801072 1.989701
H 0.646742 -7.45665 2.219581
H 3.112241 -7.111055 2.091568
C 0.317783 -1.4762 2.149797
H 1.023537 -1.421994 2.998022
H 0.96572 -1.432337 1.251623
N -0.660111 -0.438544 2.054916
C -1.121957 0.265864 3.063884
C -0.661788 0.098854 4.55091
N -2.023294 1.223262 2.770187
H -2.269459 1.784887 0.115007 7.255965
C -1.507835 2.93274 1.502767
C -0.091007 3.07952 2.007497
C 0.978628 2.31937 1.51134
C 0.161671 4.040928 2.995545
C 2.274165 2.525673 1.994322
H 0.813542 1.57193 0.734531
C 1.452901 4.244005 3.48227
H -0.674283 4.634285 3.372034
C 2.516164 3.486403 2.978771
H 3.099674 1.933601 1.593727
H 1.632688 4.997639 4.251634
H 3.530043 3.647069 3.35065
C -1.737268 1.994616 0.337428
H -2.806768 1.827921 0.191833
H -1.188542 1.052615 0.410553
Br -1.110309 2.903056 -1.319407
O -2.349424 3.792829 1.781337
C -0.789088 -1.361626 5.030052
H -1.784165 -1.770871 4.79729
H -0.03832 -2.038452 4.60353
H -0.659265 -1.397465 6.123748
C 0.783611 0.608933 4.710184
H 0.8409 1.679906 4.457728
H 1.103036 0.494507 5.759531
H 1.512524 0.079265 4.08195
C -1.518746 0.942059 5.514329
H -1.413512 2.023976 5.333169
H -2.586975 0.679492 5.462673
H -1.180495 0.758551 6.546059
Ts3_r2
38
Energy=-3488.846738
C -0.302782 -2.746294 1.894106
O -1.481526 -2.992198 1.715489
C 0.714266 -3.856419 1.836157
C 2.072709 -3.644189 2.111303
C 0.274639 -5.144532 1.499737

N -4.557336 0.245834 0.11258
H -5.40198 0.048743 0.649958
C -2.421888 -3.819215 -1.902224
H -2.88544 -4.238314 -1.006944
H -3.114595 -3.172085 -2.441798
C -1.098545 -3.174211 -1.548986
O -0.537561 -3.487139 -0.513866
C -0.471172 -2.231138 -2.526025
C -1.098774 -1.870815 -3.726525
C 0.761713 -1.656463 -2.19349
C -0.506434 -0.93363 -4.572078
H -2.061454 -2.301512 -4.007102
C 1.354557 -0.718116 -3.038031
H 1.246512 -1.943833 -1.258065
C 0.718206 -0.353582 -4.227872
H -1.003985 -0.652427 -5.501667
H 2.311216 -0.268134 -2.767521
H 1.176366 0.384577 -4.888801
Br -2.007894 -5.342123 -3.073442
R1_IMC2_TS1
41
Energy=-3528.145111
C -3.547536 0.577602 0.134403
O -4.433128 1.565557 0.163197
C -2.375107 0.755488 1.129643
C -1.446696 -0.268893 1.371477
C -2.202091 1.978384 1.781995
C -0.37226 -0.072988 2.240777
H -1.576863 -1.235913 0.875377
C -1.128102 2.182349 2.656911
H -2.935339 2.763981 1.587451
C -0.207721 1.157836 2.888095
H 0.339587 -0.883025 2.418864
H -1.009206 3.145385 3.15922
H 0.631359 1.313168 3.569413
C -2.989316 0.236323 -1.304773
H -3.104342 1.120471 -1.951595
H -1.916589 -0.029975 -1.286315
N -3.766447 -0.895192 -1.81721
C -4.415894 -1.373884 -0.805939
C -5.362291 -2.533795 -0.894111
H -6.357237 -2.241474 -0.525371
H -5.45894 -2.876301 -1.93158
H -5.01534 -3.368558 -0.266797
N -4.170105 -0.784259 0.406692
H -4.934812 -0.771361 1.078515
C -2.696703 -3.869125 -2.353147
H -3.451972 -4.395998 -1.765689
N -2.255478 3.744875 -0.013082
H 0.535683 4.041221 1.759895
N -0.66636 2.495563 0.883624
C -1.500961 6.030323 0.641787
C -2.591267 6.638043 0.000828
C -0.563383 6.845561 1.297116
C -2.739366 8.025969 0.014314
C -0.713172 8.231671 1.309495
H 0.292623 6.392817 1.802821
C -1.802231 8.829886 0.667835
H -3.593869 8.48174 -0.489774
H 0.025856 8.849078 1.823722
H -1.918804 9.914936 0.67805
C 0.079793 1.308927 1.269304
H 1.004895 1.635195 1.765896
H -0.500711 0.707105 1.979532
C 0.426112 0.425838 0.068298
O 0.157298 -0.759815 0.10362
C 1.092886 1.04016 -1.119887
C 1.79631 2.24934 -1.027646
C 1.025912 0.36384 -2.346387
C 2.430692 2.773145 -2.154654
H 1.875063 2.776213 -0.074895
C 1.648859 0.895981 -3.472737
H 0.475267 -0.576768 -2.407129
C 2.352516 2.10132 -3.376843
H 2.986985 3.708505 -2.077624
H 1.587066 0.372429 -4.428014
H 2.842608 2.517628 -4.258803
H -3.32554 6.013676 -0.510978
H -2.285665 1.605017 -0.1992
R2_P3
34
Energy=-840.554475
C -0.053827 -0.0784 -0.544728
C 1.647054 1.188334 -0.801478
C 0.621874 2.013817 -0.387972
N -0.464893 1.1752 -0.223253
H 2.666595 1.478412 -1.042946
N 1.213257 -0.106973 -0.902391
C 0.63341 3.45289 -0.078535
C -0.423398 4.303772 -0.436149
C 1.731856 3.983078 0.619214
C -0.394672 5.652402 -0.075118
H -1.267923 3.918034 -1.011838
C 1.765222 5.333599 0.962405
H 2.549358 3.320672 0.914239
C 0.695665 6.170278 0.627576
H -1.22656 6.302217 -0.352646
H 2.621099 5.731477 1.510836
H 0.715034 7.22435 0.909709
C -1.800407 1.486382 0.258517
H -2.281625 0.531458 0.520782
H -2.403753 1.949743 -0.533605
C -1.897151 2.397552 1.485985
O -2.915308 3.051321 1.615111
O -2.232164 1.90385 0.000318
C -4.276207 0.699 0.000314
C -5.678226 0.73893 0.000374
C -3.613811 -0.53659 1.4e-05
C -6.40431 -0.452332 0.000146
H -6.217575 1.687098 0.000597
C -4.343282 -1.72275 -0.000218
H -2.522666 -0.553929 -3.9e-05
C -5.741309 -1.681543 -0.00015
H -7.494931 -0.420132 0.000194
H -3.82329 -2.681931 -0.000459
H -6.315267 -2.609809 -0.000335
C -4.217507 3.251802 0.000628
H -4.856293 3.316271 -0.888791
H -4.855882 3.316379 0.890335
Br -3.071814 4.806649 0.000244
R3_Ts1
48
Energy=-3719.785747
C -2.929421 1.27174 -0.360487
O -3.013744 2.501701 -0.54446
C -2.328001 0.799878 0.954495
C -1.489059 -0.316469 1.024432
C -2.586279 1.529006 2.124153
C -0.926571 -0.710062 2.244316
H -1.274302 -0.888789 0.122645
C -2.029802 1.13936 3.341135
H -3.2336 2.407317 2.065068
C -1.194924 0.01519 3.405375
H -0.275352 -1.586825 2.27887
H -2.242599 1.712594 4.246115
H -0.756929 -0.288461 4.358269
C -2.787442 0.327635 -1.576128
H -3.292677 0.862477 -2.404785
H -1.718447 0.238613 -1.848924
N -3.361215 -0.990663 -1.363811
C -4.388747 -0.857386 -0.554814
N -4.725605 0.354973 -0.101059
H -5.348565 0.348179 0.706831
C -2.428294 -3.909253 -1.869255
H -3.056457 -4.295147 -1.062184
H -3.020533 -3.358884 -2.600235
C -1.258445 -3.13845 -1.296795
O -0.92968 -3.320359 -0.138481
C -0.499507 -2.211744 -2.192139
C -0.89134 -1.960446 -3.514599
C 0.606561 -1.535148 -1.66154
C -0.201559 -1.023574 -4.282949
H -1.748163 -2.472901 -3.955111
C 1.301896 -0.603933 -2.431411
H 0.909722 -1.738737 -0.632139
C 2.975009 -4.708224 2.052684
H 2.439318 -2.652151 2.379545
C 1.176097 -6.205007 1.435762
H -0.784334 -5.301827 1.287252
C 2.529646 -5.987603 1.712982
H 4.030111 -4.5358 2.271449
H 0.825383 -7.203614 1.169552
H 3.237567 -6.817148 1.664499
C 0.194628 -1.337742 2.195426
H 0.636601 -1.370655 3.215788
H 1.043498 -1.143718 1.511488
N -0.81131 -0.327109 2.045889
C -1.074177 0.386111 3.102251
N -1.930524 1.417257 3.10893
H -1.915649 1.891494 4.01128
C -1.619355 3.10404 1.885149
C -0.10893 3.125757 2.004131
C 0.734997 2.331696 1.214008
C 0.461683 3.971757 2.963536
C 2.120949 2.385628 1.387413
H 0.321913 1.66955 0.452283
C 1.843863 4.02281 3.142743
H -0.201133 4.591438 3.571
C 2.679874 3.228639 2.350784
H 2.767223 1.767743 0.760465
H 2.272026 4.686358 3.896835
H 3.762949 3.268982 2.48236
C -2.208578 2.311626 0.732654
H -3.288936 2.212624 0.860707
H -1.738586 1.339699 0.565579
Br -1.955924 3.361368 -0.933694
O -2.283114 4.010806 2.407884
H -0.541404 0.141971 4.048544
Ts4_r2
37
Energy=-3488.327340
C 0.651898 -1.784712 1.463341
O 1.358472 -0.816799 1.242943
C 1.093477 -3.155917 1.022157
C 0.412998 -4.319377 1.409451
C 2.224915 -3.262932 0.201437
C 0.861118 -5.570111 0.979442
H -0.46644 -4.261955 2.052845
C 2.667704 -4.51057 -0.233729
H 2.749515 -2.352598 -0.094841
C 1.98568 -5.667599 0.156804
H 0.328906 -6.47144 1.288189
H 3.545631 -4.583252 -0.878037
H 2.331364 -6.64633 -0.181289
C -0.685788 -1.650926 2.19224
H -1.433621 -2.219527 1.611186
H -0.56166 -2.214821 3.1462
N -1.120561 -0.3069 2.404931
C -0.621309 0.338064 3.458281
N -0.637741 1.627536 3.614472
C -1.298491 2.376887 2.468339

H -3.161279 -3.195044 -3.076942
C -1.696651 -3.198377 -1.432276
O -1.709896 -3.468796 -0.244722
C -0.693806 -2.255076 -2.010739
C -0.821595 -1.740997 -3.3086
C 0.339606 -1.797281 -1.182655
C 0.071886 -0.774116 -3.766678
H -1.630656 -2.068034 -3.964219
C 1.239453 -0.838556 -1.643986
H 0.428193 -2.199255 -0.171268
C 1.10287 -0.323799 -2.936602
H -0.037691 -0.368977 -4.773712
H 2.043273 -0.487336 -0.994945
H 1.800242 0.434166 -3.298209
Br -1.726201 -5.235654 -3.364599
R1_IMC2_TS2
41
Energy=-3528.196772
C -2.79753 1.298038 -0.437371
O -3.247303 2.421677 -0.5707
C -1.801801 0.997346 0.641026
C -0.745278 0.109585 0.415544
C -1.90754 1.657354 1.871453
C 0.203171 -0.119822 1.413974
H -0.641075 -0.3801 -0.553992
C -0.971205 1.414731 2.875197
H -2.736352 2.349402 2.036438
C 0.086519 0.526713 2.646571
H 1.034418 -0.803051 1.226259
H -1.062288 1.91954 3.838553
H 0.821311 0.341695 3.432284
C -3.14793 0.215454 -1.455964
H -4.073553 0.542211 -1.961138
H -2.339225 0.204569 -2.205483
N -3.278447 -1.126776 -0.917285
C -4.139842 -1.293186 0.138334
C -4.393122 -2.703395 0.616475
H -3.469831 -3.296225 0.639144
H -4.832736 -2.673303 1.620676
H -5.104649 -3.213631 -0.050319
N -4.663955 -0.240388 0.671452
H -5.231381 -0.501003 1.478228
C -2.94143 -2.237612 -1.813145
H -3.649326 -3.062395 -1.687603
H -3.029044 -1.905014 -2.853117
C -1.541992 -2.780729 -1.493726
C -0.797378 2.477059 2.494424
C 0.102782 1.42614 2.717651
C -0.665954 3.676129 3.209147
C 1.132726 1.582308 3.645886
H -0.002947 0.479634 2.182791
C 0.373343 3.834288 4.123412
H -1.371258 4.488631 3.020833
C 1.276529 2.788284 4.33809
H 1.828697 0.76139 3.825524
H 0.485522 4.77546 4.664314
H 2.093765 2.911753 5.051156
H -0.730926 -0.927102 -0.489676
R2_pHCOBr
17
Energy=-2955.922823
C -3.443975 1.94489 0.000524
O -2.232164 1.90385 0.000318
C -4.276207 0.699 0.000314
C -5.678226 0.73893 0.000374
C -3.613811 -0.53659 1.4e-05
C -6.40431 -0.452332 0.000146
H -6.217575 1.687098 0.000597
C -4.343282 -1.72275 -0.000218
H -2.522666 -0.553929 -3.9e-05
C -5.741309 -1.681543 -0.00015
H -7.494931 -0.420132 0.000194
H -3.82329 -2.681931 -0.000459
H -6.315267 -2.609809 -0.000335
C -4.217507 3.251802 0.000628
H -4.856293 3.316271 -0.888791
H -4.855882 3.316379 0.890335
Br -3.071814 4.806649 0.000244
R2_Ts1
38
Energy=-3488.844307
C -3.00356 1.279925 -0.370886
O -3.189975 2.501657 -0.566598
C -2.382566 0.878557 0.962128
C -1.45176 -0.160851 1.071758
C -2.723886 1.606087 2.111154
C -0.887425 -0.48095 2.312304
H -1.16993 -0.736765 0.188549
C -2.16513 1.289064 3.348138
H -3.439446 2.426605 2.018536
C -1.241983 0.240375 3.453002
H -0.164194 -1.296764 2.382189
H -2.444641 1.860687 4.235842
H -0.800686 -0.007768 4.420332
C -2.702464 0.355366 -1.572173
H -3.197441 0.839845 -2.436316
H -1.615035 0.349201 -1.780146
N -3.183378 -1.004491 -1.381456
C -4.240149 -0.949232 -0.6142
C 0.894652 -0.344707 -3.743169
H -0.519088 -0.824562 -5.307663
H 2.158615 -0.077261 -2.008011
H 1.431368 0.389933 -4.346405
Br -1.668433 -5.467578 -2.786421
C -5.129768 -2.091147 -0.13135
C -5.446817 -2.300982 1.217758
C -5.520307 -3.048379 -1.077332
C -6.122707 -3.456108 1.616695
H -5.145947 -1.562077 1.965608
C -6.209333 -4.196646 -0.68224
H -5.28651 -2.88324 -2.131459
C -6.509396 -4.405663 0.666982
H -6.349339 -3.614274 2.672869
H -6.516968 -4.929848 -1.430707
H -7.044719 -5.305137 0.976953
R3_Ts
48
Energy=-3719.781221
C -2.194392 1.29248 -0.397068
O -2.26386 2.501356 -0.542522
C -1.742769 0.715186 0.913755
C -1.024156 -0.487166 0.972847
C -2.005929 1.425822 2.091382
C -0.573388 -0.970161 2.203036
H -0.810483 -1.039279 0.054322
C -1.573939 0.931386 3.321828
H -2.562105 2.363941 2.032616
C -0.855145 -0.267036 3.377534
H -0.003121 -1.899879 2.245332
H -1.794904 1.480629 4.238746
H -0.512337 -0.653002 4.339535
C -2.461927 0.34515 -1.563859
H -3.091931 0.913764 -2.275479
H -1.483299 0.183701 -2.051898
N -3.018606 -0.937121 -1.205217
C -4.22732 -0.852874 -0.623169
N -4.835131 0.28585 -0.381526
H -5.70216 0.11686 0.130834
C -3.137708 -2.165074 -2.994888
H -4.093887 -2.534504 -2.636475
H -3.10275 -1.205254 -3.50107
C -1.910071 -2.862734 -2.49351
O -1.996067 -3.905858 -1.871023
C -0.569808 -2.254929 -2.791624
C -0.376636 -1.294345 -3.794198
C 0.524615 -2.691393 -2.034419
C 0.899183 -0.780315 -4.03167
H -1.212539 -0.956407 -4.410055
C -0.398591 3.641969 2.341821
C 0.987607 3.552613 2.154495
C -0.963788 4.906229 2.501524
C 1.78926 4.694682 2.1422
H 1.453572 2.568529 2.042436
C -0.170781 6.059971 2.488701
H -2.045732 4.952859 2.64397
C 1.210664 5.960382 2.304441
H 2.869685 4.603088 2.004054
H -0.631986 7.04207 2.623714
H 1.834588 6.856932 2.296684
C -1.115871 1.515318 1.168935
H -1.998696 1.064111 0.735555
H -0.141739 1.147391 0.848161
Br -1.182685 3.063172 -0.620432
O -2.582638 2.659845 2.700382
H -0.163788 -0.266141 4.272322
Ts3_r1
41
Energy=-3528.128648
C -0.329735 -2.646689 2.186892
O -1.411805 -2.836064 2.711651
C 0.668673 -3.769164 2.086847
C 1.954221 -3.581466 1.559532
C 0.292962 -5.038863 2.547696
C 2.848088 -4.651988 1.492836
H 2.271197 -2.601399 1.199435
C 1.183885 -6.107806 2.477492
H -0.708783 -5.176501 2.958649
C 2.464741 -5.914981 1.949691
H 3.847485 -4.498043 1.082565
H 0.88168 -7.093765 2.834756
H 3.164687 -6.750877 1.894865
C 0.063369 -1.284818 1.619422
H 0.959927 -0.952757 2.18515
H 0.422015 -1.463164 0.590627
N -0.999314 -0.317613 1.582429
C -1.242236 0.37316 2.663694
C -0.450789 0.173766 3.948654
H -0.33389 -0.886291 4.213783
H 0.560337 0.601277 3.823412
H -0.928541 0.689023 4.792281
N -2.150152 1.380857 2.617467
H -2.219173 1.834374 3.528517
C -1.538554 3.087138 1.678606
C -0.072813 2.982904 2.061681
C 0.863673 2.23074 1.33689
C 0.346509 3.636862 3.227133
C 2.185469 2.128847 1.781415
H 0.574125 1.719471 0.417711
C 1.665064 3.538736 3.672624
H -0.386474 4.224356 3.784243
C 2.591211 2.780754 2.94883
H 2.904056 1.541691 1.20544
H 1.972231 4.054751 4.584733
H 3.624927 2.700691 3.29088

O -1.400487 -3.520839 -0.534116
C -0.337871 -2.226999 -2.204074
C -0.427111 -1.470066 -3.37968
C 0.909108 -2.382345 -1.584056
C 0.717822 -0.898435 -3.935705
H -1.38469 -1.354298 -3.888744
C 2.055762 -1.816892 -2.140905
H 0.967551 -2.952068 -0.654338
C 1.960762 -1.070854 -3.319317
H 0.640276 -0.321035 -4.858664
H 3.022021 -1.94833 -1.650361
H 2.854422 -0.62175 -3.757019
Br -2.181355 -4.563472 -3.86711
R1_P1
22
Energy=-496.356650
C 0.22675 0.138782 -0.017868
C 1.913227 1.460817 -0.225684
C 0.815791 2.28068 -0.058701
N -0.243219 1.408489 0.066158
H -1.214166 1.656171 0.232986
H 2.947607 1.757316 -0.37857
N 1.537095 0.141606 -0.199797
C -0.648692 -1.064173 0.102232
H -1.05742 -1.161121 1.118546
H -1.495002 -1.015248 -0.597105
H -0.062031 -1.962946 -0.120914
C 0.687598 3.742585 -0.025005
C -0.555651 4.378079 -0.173835
C 1.833963 4.536971 0.152575
C -0.648675 5.770573 -0.142624
H -1.463311 3.791032 -0.329721
C 1.738254 5.926722 0.173962
H 2.807352 4.059755 0.284177
C 0.495622 6.551788 0.029215
H -1.624003 6.246254 -0.26051
H 2.639842 6.526107 0.313056
H 0.420919 7.640185 0.051138
R1_P2
37
Energy=-879.846765
C -1.830917 2.494425 0.090776
C -0.367165 3.756044 1.160712
C -1.333959 4.541513 0.571496
N -2.236536 3.735779 -0.089287
H 0.505712 3.988072 1.763705
N -0.693553 2.46176 0.84464
C -2.487198 1.263446 -0.441934
H -2.588731 0.490321 0.333834
H -3.487747 1.52177 -0.808242
H -1.921922 0.829157 -1.282055
C -1.466646 6.008593 0.591017
C -2.535865 6.631785 -0.070602
N -4.689482 0.228621 -0.170931
H -5.302945 0.167265 0.641392
C -2.513022 -3.748367 -1.87806
H -3.204391 -4.013253 -1.07355
H -3.026694 -3.207649 -2.671367
C -1.293379 -3.055436 -1.305807
O -0.986125 -3.254402 -0.144058
C -0.44454 -2.206848 -2.200571
C -0.773728 -1.959639 -3.540087
C 0.697209 -1.609666 -1.651157
C 0.019991 -1.110487 -4.310876
H -1.656885 -2.412176 -3.993975
C 1.490827 -0.759499 -2.420246
H 0.949764 -1.809708 -0.607678
C 1.151165 -0.50809 -3.752431
H -0.245849 -0.917722 -5.351461
H 2.373789 -0.292235 -1.981086
H 1.767374 0.159796 -4.357274
Br -1.877344 -5.439392 -2.654147
H -4.72716 -1.895972 -0.32935
R2_Ts2
38
Energy=-3488.845661
C -2.906481 1.300611 -0.392453
O -3.548062 2.337537 -0.383934
C -1.757089 1.11974 0.555845
C -0.625195 0.373189 0.202057
C -1.808422 1.758255 1.801442
C 0.447923 0.272934 1.090161
H -0.578459 -0.119391 -0.772327
C -0.744867 1.640142 2.695365
H -2.69521 2.338787 2.064805
C 0.386066 0.898761 2.338204
H 1.334098 -0.299152 0.807847
H -0.794667 2.127476 3.670655
H 1.221698 0.80934 3.034996
C -3.207879 0.204822 -1.414821
H -4.237118 0.402572 -1.772083
H -2.525907 0.379117 -2.270405
N -3.024758 -1.146518 -0.952725
C -3.785774 -1.527959 0.071809
N -4.586875 -0.750231 0.755112
H -5.02735 -1.289074 1.501425
C -3.084982 -2.554362 -2.58182
H -4.035077 -2.939362 -2.2216
H -3.103701 -1.6687 -3.20834
C -1.860088 -3.02794 -1.873634
O -1.930547 -3.940035 -1.064936
C 1.795109 -2.163644 -2.259155
H 0.364259 -3.443139 -1.259098
C 1.984287 -1.208389 -3.262345
H 1.045387 -0.040491 -4.820422
H 2.640003 -2.499292 -1.655287
H 2.978912 -0.797619 -3.445897
Br -3.241651 -3.358608 -4.946989
C -4.838503 -2.169871 -0.222554
C -6.218859 -2.384633 -0.341692
C -4.031676 -3.195431 0.285828
C -6.783336 -3.601807 0.044154
H -6.855828 -1.598678 -0.755881
C -4.596896 -4.407023 0.68786
H -2.9556 -3.031832 0.36614
C -5.973323 -4.614839 0.56552
H -7.857779 -3.761147 -0.065518
H -3.959306 -5.195591 1.092864
H -6.414299 -5.56526 0.872256
R4_I1.xyz
33
Energy=-690.063449
C -0.147261 -0.927715 -0.391364
O -0.222638 -2.143204 -0.353031
C -0.413106 -0.213224 -1.696963
C -0.358282 1.182958 -1.81793
C -0.727143 -0.984779 -2.824571
C -0.6145 1.793112 -3.047592
H -0.116912 1.807558 -0.956451
C -0.982656 -0.376398 -4.052262
H -0.768324 -2.07081 -2.723974
C -0.926633 1.016219 -4.165658
H -0.569811 2.880267 -3.131586
H -1.225966 -0.987351 -4.923378
H -1.126729 1.495809 -5.12569
C 0.201258 -0.074868 0.812642
H 1.069765 0.557592 0.519365
H -0.622545 0.665289 0.929596
N 0.442087 -0.825932 2.006687
C 0.740699 -0.067475 3.051107
C 1.00646 -0.807737 4.389114
N 0.816482 1.263753 3.013185
H 1.059586 1.619408 3.938487
C 0.909674 -2.330023 4.247447
H -0.089459 -2.644779 3.913299
H 1.641797 -2.71817 3.524297
H 1.10869 -2.803497 5.223173
C 2.416421 -0.462224 4.900879
H 2.534184 0.614044 5.099882
H 2.621445 -0.997086 5.842613
H 3.186906 -0.75779 4.170756
C -0.027652 -0.354986 5.435671
C -1.937933 2.490982 0.342223
H -3.026542 2.479293 0.250169
H -1.511273 1.504872 0.143162
Br -1.290123 3.691524 -1.099921
O -2.225021 4.005229 2.168128
Ts3_r3
48
Energy=-3719.779719
C -0.455662 -2.921657 1.919083
O -1.652546 -3.123898 2.018022
C 0.500595 -4.081484 1.829341
C 1.889018 -3.903702 1.744865
C -0.026371 -5.380825 1.841535
C 2.734978 -5.012253 1.675522
H 2.324587 -2.903323 1.736379
C 0.818054 -6.486743 1.769105
H -1.107943 -5.511057 1.90873
C 2.202108 -6.303297 1.68604
H 3.81447 -4.865799 1.612016
H 0.398905 -7.494407 1.778139
H 2.865838 -7.168184 1.630979
C 0.127295 -1.513248 1.89828
H 0.811875 -1.441765 2.767984
H 0.780453 -1.459982 1.007144
N -0.855922 -0.469837 1.820079
C -1.148529 0.24027 2.877629
N -1.96082 1.313438 2.779605
H -2.054569 1.785506 3.679262
C -1.492982 2.986129 1.647373
C -0.036914 3.086926 2.062075
C 0.96196 2.236765 1.563498
C 0.312561 4.059132 3.006929
C 2.277328 2.349663 2.020269
H 0.721233 1.47749 0.817999
C 1.627747 4.178225 3.460092
H -0.469893 4.719809 3.385355
C 2.615413 3.319763 2.968593
H 3.045084 1.680756 1.625915
H 1.883167 4.943081 4.19641
H 3.645566 3.408039 3.31922
C -1.78871 2.198641 0.384519
H -2.869009 2.092652 0.257663
H -1.28734 1.230139 0.327888
Br -1.159038 3.255747 -1.168038
O -2.2876 3.86549 2.018592
C -0.579607 -0.076933 4.243451
C 0.232343 0.856798 4.900426
C -0.861136 -1.297651 4.871554
C 0.762372 0.570771 6.160974
H 0.451328 1.814421 4.419006
C -0.344224 -1.57802 6.138253
H -1.497942 -2.027931 4.365518
C 0.473098 -0.646073 6.784141
H 1.401217 1.302691 6.659297
H -0.579581 -2.527976 6.62228
H 0.881805 -0.866841 7.771992

C -0.531679 6.811846 1.265336
C -2.666029 8.021647 -0.058829
C -0.663387 8.199682 1.275994
H 0.308605 6.347572 1.786759
C -1.731655 8.812983 0.613546
H -3.504484 8.488809 -0.579229
H 0.0737 8.806899 1.805054
H -1.833875 9.899499 0.622234
C 0.022395 1.266943 1.251965
H 0.902704 1.577837 1.833556
H -0.610304 0.639984 1.894692
C 0.48073 0.413659 0.066502
O 0.310423 -0.789988 0.097183
C 1.122838 1.080846 -1.10679
C 1.817326 2.291884 -0.979202
C 1.034896 0.453448 -2.357266
C 2.426842 2.863898 -2.096429
H 1.910213 2.779734 -0.006897
C 1.629303 1.036561 -3.474417
H 0.489645 -0.488673 -2.444497
C 2.327081 2.241687 -3.343434
H 2.97915 3.799149 -1.993172
H 1.549764 0.552486 -4.448998
H 2.79585 2.697209 -4.217623
H -3.268019 6.017429 -0.597379
R1_P3
37
Energy=-879.841649
C -0.071794 -0.098346 -0.449599
C 1.630649 1.180488 -0.740044
C 0.608753 2.010113 -0.33971
N -0.478555 1.170888 -0.152142
H 2.648067 1.467164 -0.994793
N 1.197836 -0.117988 -0.812718
C -0.963077 -1.291379 -0.342439
H -1.280821 -1.46915 0.696572
H -1.867494 -1.18605 -0.958933
H -0.409543 -2.17326 -0.685206
C 0.621994 3.455544 -0.063345
C -0.432548 4.301725 -0.438933
C 1.724112 4.000684 0.61783
C -0.398172 5.658995 -0.112316
H -1.280926 3.904701 -1.001219
C 1.763895 5.359408 0.925775
H 2.539723 3.343055 0.928557
C 0.696234 6.191297 0.573098
H -1.22904 6.304195 -0.403532
H 2.622911 5.768037 1.461294
H 0.720086 7.252248 0.827927
C -1.806485 1.510222 0.32959
H -2.297106 0.57655 0.644174
H -2.418927 1.942452 -0.473687
C -1.882817 2.467175 1.522525
O -2.87817 3.159361 1.625808
C -0.78879 2.530403 2.537487
C 0.071341 1.451977 2.785294
C -0.552628 -2.359108 -2.184905
C -0.411685 -1.39434 -3.191685
C 0.549738 -2.683077 -1.385577
C 0.811687 -0.747153 -3.375556
H -1.246227 -1.142379 -3.847719
C 1.770357 -2.033504 -1.564129
H 0.42994 -3.43485 -0.603299
C 1.902222 -1.060802 -2.559789
H 0.91319 0.004102 -4.160725
H 2.620181 -2.283385 -0.926182
H 2.855426 -0.54797 -2.701762
Br -2.921241 -4.00686 -4.324081
H -3.673373 -2.598762 0.322829
R3_Br
R3_I1
31
Energy=-763.876987
C 0.149101 -0.892294 -0.382876
O 0.44324 -2.074626 -0.386665
C -0.230142 -0.217976 -1.680747
C -0.561637 1.142961 -1.753345
C -0.246495 -0.989022 -2.851387
C -0.902474 1.719223 -2.978673
H -0.556337 1.766609 -0.858079
C -0.587268 -0.414375 -4.07461
H 0.011968 -2.047569 -2.788087
C -0.916004 0.943307 -4.140034
H -1.157882 2.779228 -3.025424
H -0.596963 -1.024502 -4.97952
H -1.182544 1.396637 -5.096667
C 0.143152 -0.040392 0.87194
H 0.81229 0.828079 0.677171
H -0.865613 0.425347 0.942262
N 0.503627 -0.756374 2.055737
C 0.531863 0.00153 3.141085
N 0.24503 1.300384 3.179351
H 0.3835 1.658836 4.123884
C 0.928966 -0.734137 4.407581
C 0.437861 -0.342222 5.661255
C 1.800551 -1.831724 4.348659
C 0.804475 -1.027662 6.822697
H -0.258794 0.496524 5.736451
C 2.178575 -2.511909 5.507613
H 2.181961 -2.146652 3.37595
C 1.68036 -2.113144 6.751505
H 0.39996 -0.712867 7.786827
H 2.866333 -3.357564 5.440089
H 1.970891 -2.646781 7.658531
R3_I3_O_neg
31
Energy=-763.881887
C -0.802721 0.191496 -0.135069
C 0.757927 0.34215 -0.291999
C -0.016646 2.368417 0.090244
N -1.135939 1.673608 -0.309668
H 0.138299 -0.881572 6.389771
H 0.03465 0.725452 5.637403
H -1.053462 -0.578517 5.101245
R4_IMC1.xyz
50
Energy=-3645.981908
C -1.754682 0.961926 -0.261034
O -1.88329 2.068612 -0.759381
C -1.607472 0.828507 1.23171
C -0.64987 -0.030463 1.785481
C -2.415152 1.601678 2.072795
C -0.495157 -0.105217 3.17163
H -0.022354 -0.636587 1.128797
C -2.282962 1.503331 3.458969
H -3.160922 2.266816 1.630734
C -1.319253 0.651938 4.009219
H 0.261966 -0.765046 3.599369
H -2.928259 2.094105 4.111859
H -1.209685 0.579782 5.093095
C -1.623885 -0.299914 -1.115077
H -2.150592 -0.059865 -2.062721
H -0.549651 -0.396853 -1.369653
N -2.073328 -1.51216 -0.473771
C -3.342554 -1.431952 -0.081638
C -3.85844 -2.556563 0.848915
N -4.124483 -0.397074 -0.372231
H -5.037343 -0.512095 0.069903
C -3.057345 -3.002394 -2.794292
H -3.646227 -3.468149 -2.00153
H -3.237131 -1.925046 -2.839313
C -1.604233 -3.42109 -2.685233
O -1.318144 -4.494907 -2.187853
C -0.559112 -2.517275 -3.255078
C -0.87402 -1.468485 -4.12957
C 0.774987 -2.739089 -2.889619
C 0.137071 -0.640301 -4.617971
H -1.90594 -1.282118 -4.434174
C 1.782699 -1.90477 -3.368562
H 1.008494 -3.56163 -2.211001
C 1.463545 -0.854439 -4.235675
H -0.112155 0.175579 -5.298261
H 2.818376 -2.071564 -3.067986
H 2.251519 -0.200147 -4.613335
Br -3.722221 -3.771823 -4.484478
C -2.905326 -3.754876 0.901519
H -3.326159 -4.531712 1.561341
H -2.746397 -4.198414 -0.093087
H -1.919927 -3.467998 1.29724
C -3.957022 -1.95203 2.264123
R4_Ts5
50
Energy=-3645.944188
C -0.374184 -2.855413 1.987277
O -1.485702 -3.221574 2.321261
C 0.726076 -3.868106 1.794353
C 2.024931 -3.502752 1.411867
C 0.431202 -5.220562 2.017335
C 3.011207 -4.478751 1.255311
H 2.280824 -2.457407 1.232283
C 1.415321 -6.194467 1.860069
H -0.581589 -5.497024 2.315888
C 2.708771 -5.823895 1.478236
H 4.019435 -4.186053 0.957498
H 1.176041 -7.244754 2.035392
H 3.481719 -6.584938 1.355355
C -0.031703 -1.38642 1.752875
H 0.837701 -1.157175 2.392193
H 0.359551 -1.334017 0.718484
N -1.152431 -0.502578 1.859657
C -1.450006 0.197549 2.920252
N -2.518884 1.036103 2.809665
H -2.768306 1.408349 3.721986
C -1.440443 2.539233 1.036295
C -2.321154 2.256352 -0.145961
C -3.67486 2.6141 -0.190604
C -1.754818 1.573882 -1.228371
C -4.447282 2.290458 -1.306761
H -4.137202 3.151981 0.638225
C -2.52492 1.250582 -2.344843
H -0.70146 1.291741 -1.178059
C -3.874246 1.612533 -2.386603
H -5.500995 2.573161 -1.33574
H -2.074303 0.715443 -3.182579
H -4.481894 1.361799 -3.258193
C -2.045174 3.121915 2.26322
H -1.395734 3.211057 3.13045
H -3.114224 3.215727 2.409494
Br -1.826947 5.3183 1.601668
O -0.242706 2.312221 0.99779
C -0.634891 0.199412 4.256049
C 0.693358 0.949964 4.032763
H 0.500914 1.983755 3.707168
H 1.260326 0.988006 4.977763
H 1.338506 0.485679 3.274815
C -1.374505 0.958339 5.375232
H -0.769491 0.916137 6.294143
H -1.519704 2.024122 5.138072
H -2.354748 0.511122 5.605831
C -0.379949 -1.221332 4.800089
H -1.305401 -1.816986 4.808747
H 0.380115 -1.783735 4.242644
H -0.016955 -1.151185 5.83801
R2_TS6
36
Energy=-916.421474

C -0.61957 3.736805 3.230808
C 1.100876 1.588006 3.71711
H -0.066446 0.500181 2.266152
C 0.419324 3.874486 4.148747
H -1.294134 4.570266 3.021878
C 1.28328 2.800855 4.388075
H 1.76598 0.746319 3.916627
H 0.562018 4.820618 4.673529
H 2.100129 2.908212 5.104206
R1_pHCOBr
17
Energy=-2955.922823
C -3.444017 1.944876 0.000316
O -2.232164 1.903843 0.000219
C -4.276141 0.69898 0.000211
C -5.67818 0.738864 0.000385
C -3.613806 -0.536658 -9.9e-05
C -6.404293 -0.452379 0.000205
H -6.217548 1.687052 0.000619
C -4.3433 -1.722799 -0.000238
H -2.522639 -0.554036 -
0.000234
C -5.741331 -1.681593 -7.3e-05
H -7.494936 -0.420133 0.000318
H -3.823286 -2.681992 -
0.000477
H -6.315324 -2.60986 -0.000189
C -4.217514 3.25183 0.000611
H -4.856097 3.316678 -0.888938
H -4.856045 3.316371 0.890215
Br -3.071889 4.806807 0.000843
R1_Ts1
41
Energy=-3528.130909
C -2.917926 1.301707 -0.397683
O -3.039449 2.525587 -0.600161
C -2.354949 0.867293 0.947141
C -1.48075 -0.218297 1.068203
C -2.681844 1.606969 2.092644
C -0.95802 -0.573555 2.317338
H -1.210891 -0.799316 0.185659
C -2.165064 1.254578 3.338086
H -3.353625 2.462756 1.9914
C -1.298316 0.159531 3.454692
H -0.278763 -1.425786 2.394499
H -2.432958 1.834547 4.223932
H -0.890802 -0.11575 4.429466
C -2.688861 0.345879 -1.587019
H -3.164802 0.852126 -2.450793
H -1.604231 0.288816 -1.804826
N -3.228068 -0.985995 -
1.379596
C -4.300522 -0.889012 -0.624541
C -5.006671 -2.159749 -0.199997
H -6.024322 -1.954639 0.159558
H -2.012176 1.929029 0.143287
H 1.267868 -0.39878 0.342629
N 1.092508 1.707373 0.111432
H 1.091543 0.184923 -1.333616
C -1.404969 -0.567838 -1.342833
C -2.082204 -1.769519 -1.11983
C -1.273117 -0.112266 -2.663846
C -2.61891 -2.50376 -2.183751
H -2.182196 -2.107938 -
0.086512
C -1.806611 -0.83803 -3.730615
H -0.751477 0.82886 -2.858989
C -2.482978 -2.040532 -3.494004
H -3.145569 -3.44086 -1.988248
H -1.697692 -0.464808 -
4.751498
H -2.900794 -2.609482 -4.32705
O -1.192167 -0.278873 1.040885
C -0.098556 3.813883 0.434868
C -1.240746 4.565354 0.128173
C 0.980679 4.440566 1.075009
C -1.303071 5.921673 0.456754
H -2.08344 4.096858 -0.384225
C 0.917591 5.793812 1.402575
H 1.868211 3.853344 1.317015
C -0.225722 6.539071 1.094828
H -2.196925 6.497138 0.209385
H 1.762411 6.270198 1.903383
H -0.275146 7.59875 1.351955
R3_I3_r3
32
Energy=-764.352978
C -0.897902 0.183775 -0.195616
C 0.651563 0.254121 -0.089466
C -0.095613 2.283178 0.295552
N -1.21077 1.629973 -0.188668
H 1.025018 -0.525076 0.590302
N 0.981081 1.597227 0.392001
H 1.122637 0.096121 -1.071409
C -1.406499 -0.517551 -1.44459
C -2.245529 -1.630721 -1.36595
C -1.036635 -0.021341 -2.701858
C -2.713604 -2.240885 -2.535176
H -2.539574 -2.018438 -
0.390666
C -1.501949 -0.629859 -3.865792
H -0.392156 0.85956 -2.768855
C -2.345384 -1.743765 -3.785039
H -3.371407 -3.109096 -
2.463729
H -1.209776 -0.232701 -
4.839547
H -2.712609 -2.220092 -
4.695801
O -1.481045 -0.446695 0.921224
H -1.089108 -0.075191 1.729762
H -4.68262 -1.123886 2.301608
H -4.279217 -2.718244 2.988766
H -2.9785 -1.563316 2.593889
C -5.251757 -3.054946 0.429412
H -5.246577 -3.487327 -
0.583789
H -5.591202 -3.84055 1.123578
H -6.009003 -2.25633 0.448202
R4_IMC2_Ts1.xyz
50
Energy=-3645.986434
C -3.480002 1.263895 -0.016714
O -3.614951 2.572884 0.068523
C -2.690581 0.674216 1.186999
C -1.527527 -0.094029 1.075777
C -3.183801 0.934336 2.478081
C -0.881909 -0.603773 2.212222
H -1.100011 -0.292801 0.089106
C -2.548856 0.434429 3.612577
H -4.082066 1.549921 2.582194
C -1.389932 -0.344089 3.483329
H 0.024196 -1.203982 2.094732
H -2.954089 0.64953 4.604061
H -0.889631 -0.740573 4.368962
C -2.985979 0.683504 -1.377752
H -3.412139 1.334263 -2.16453
H -1.891869 0.691385 -1.504772
N -3.534656 -0.67174 -1.499951
C -4.551117 -0.705609 -0.697615
C -5.382613 -1.953204 -0.446496
N -4.807144 0.457008 -0.016452
H -5.301406 0.378406 0.870718
C -1.400302 -2.990282 -0.287501
H -1.471523 -2.993092 0.803895
H -2.1884 -2.369469 -0.736155
C 0.008404 -2.615292 -0.710387
O 0.912517 -2.676163 0.103259
C 0.24516 -2.17809 -2.118528
C -0.8048 -2.034363 -3.037221
C 1.561806 -1.903046 -2.516252
C -0.537365 -1.592936 -4.33388
H -1.838483 -2.233124 -
2.745331
C 1.826585 -1.473279 -3.813449
H 2.370788 -2.024214 -1.793687
C 0.775402 -1.320262 -4.724477
H -1.357169 -1.470239 -
5.043304
H 2.851997 -1.259458 -4.118796
H 0.981087 -0.981317 -5.741537
Br -1.705712 -4.833798 -
0.867088
C -4.906198 -2.557289 0.88946
H -5.527482 -3.428069 1.151568
H -3.861227 -2.897176 0.819656
H -4.975829 -1.828952 1.713772
C -0.040545 -2.074827 2.019854
O -0.869695 -2.244485 2.893668
C 0.522139 -3.258043 1.277089
C 1.676821 -3.168351 0.487817
C -0.141244 -4.488385 1.389996
C 2.162316 -4.297981 -0.174986
H 2.214036 -2.223029 0.393026
C 0.335614 -5.6119 0.718024
H -1.041497 -4.54572 2.005741
C 1.491024 -5.517767 -0.065404
H 3.066878 -4.223456 -0.780886
H -0.192166 -6.563547 0.802839
H 1.86776 -6.397053 -0.591274
C 0.492035 -0.678832 1.678712
H 1.480959 -0.615343 2.186373
H 0.717619 -0.642703 0.600402
N -0.373603 0.414698 2.001678
C -0.828147 0.568912 3.239989
N -1.868081 1.328341 3.495389
H -0.349373 0.050914 4.087771
C -2.354472 1.90664 2.251125
H -3.426401 2.157193 2.349445
H -1.826074 2.850695 2.012072
C -2.162451 1.036383 0.99549
O -1.934912 1.575015 -0.098846
C -2.741321 -0.361783 0.993428
C -3.670526 -0.789065 1.947099
C -2.342545 -1.247928 -
0.017763
C -4.174598 -2.093373 1.904794
H -4.000642 -0.105255 2.732191
C -2.837229 -2.550101 -
0.056641
H -1.629186 -0.900991 -0.76975
C -3.754351 -2.978868 0.910869
H -4.900961 -2.417907 2.653224
H -2.50841 -3.236177 -0.840894
H -4.14511 -3.998256 0.882706
R2_Ts5
38
Energy=-3488.831752
C -0.380668 -2.730703 1.9133
O -1.545153 -3.081845 1.865705
C 0.719779 -3.757878 1.861969
C 2.068257 -3.419843 2.044721
C 0.371625 -5.09584 1.629704
C 3.051968 -4.409563 1.996381
H 2.364008 -2.385955 2.230197
C 1.35404 -6.082503 1.576632
H -0.680289 -5.350657 1.48891
C 2.69743 -5.739817 1.760899
H 4.099132 -4.13963 2.142536
H 1.074481 -7.121042 1.391359
H 3.468722 -6.511275 1.720019
C 0.01939 -1.265335 2.033962
H 0.548031 -1.163297 3.006934

H -5.067584 -2.872375 -1.033655
H -4.449402 -2.644767 0.6174
N -4.702842 0.317219 -0.207979
H -5.359406 0.284405 0.572777
C -2.43316 -3.85115 -1.797958
H -3.055538 -4.222327 -0.980048
H -3.027687 -3.302907 -2.529438
C -1.249213 -3.086593 -1.243512
O -0.925028 -3.253408 -0.081215
C -0.457601 -2.204818 -2.156731
C -0.843903 -1.957144 -3.481056
C 0.678965 -1.570306 -1.640417
C -0.109539 -1.073145 -4.270248
H -1.729596 -2.433721 -3.904717
C 1.413916 -0.685096 -2.428484
H 0.974571 -1.768616 -0.607902
C 1.018667 -0.4353 -3.745349
H -0.419609 -0.879564 -5.298343
H 2.294113 -0.189534 -2.015685
H 1.58886 0.259829 -4.364449
Br -1.703295 -5.432102 -2.706036
R1_Ts2
41
Energy=-3528.129660
C -2.745609 1.255526 -0.478966
O -3.28235 2.340436 -0.625465
C -1.686512 1.074887 0.569078
C -0.589475 0.235376 0.336594
C -1.768418 1.799896 1.763501
C 0.422174 0.127125 1.291874
H -0.522971 -0.323476 -0.599973
C -0.768387 1.673662 2.728265
H -2.626552 2.454194 1.93268
C 0.329087 0.83926 2.491306
H 1.282415 -0.518508 1.101234
H -0.841361 2.22849 3.665345
H 1.114505 0.745256 3.243771
C -3.044302 0.0862 -1.414233
H -4.003827 0.333438 -1.908391
H -2.257952 0.113083 -2.192867
N -3.045134 -1.222231 -0.804023
C -4.003942 -1.395506 0.12246
C -4.156989 -2.805069 0.666333
H -3.250059 -3.401396 0.510798
H -4.395411 -2.780367 1.739239
H -4.989599 -3.31624 0.157045
N -4.781241 -0.417484 0.530836
H -0.170676 3.34444 0.542921
C -2.532203 2.107719 0.018003
C -3.521708 1.869533 -0.945549
C -2.855016 2.816888 1.182414
C -4.820896 2.333058 -0.736894
H -3.270114 1.335457 -1.862991
C -4.15124 3.300283 1.370572
H -2.092746 2.981219 1.946775
C -5.140383 3.055474 0.416062
H -5.585248 2.141394 -1.492053
H -4.39005 3.857335 2.278323
H -6.155519 3.425212 0.568834
R3_I5
47
Energy=-1147.848362
C 2.242807 -0.148722 0.096572
O 3.373915 -0.349696 0.498543
C 1.638447 1.219795 0.117424
C 0.333882 1.459117 -0.336878
C 2.408322 2.283397 0.610414
C -0.19327 2.750936 -0.29606
H -0.281106 0.645076 -0.724549
C 1.880757 3.571492 0.649724
H 3.423024 2.08671 0.961055
C 0.57793 3.806028 0.196587
H -1.209184 2.93281 -0.649868
H 2.483417 4.396095 1.033882
H 0.163583 4.815408 0.227522
C 1.394179 -1.30736 -0.435029
H 0.470055 -1.372294 0.160969
H 1.074802 -1.094199 -1.467879
N 2.073665 -2.578567 -0.37256
C 2.976441 -2.915767 -1.354162
N 3.377533 -2.151827 -2.311714
H 3.034474 -1.193578 -2.221505
C 2.077828 -3.203795 0.934532
H 2.893083 -3.936242 1.010109
H 2.282571 -2.443147 1.709423
C 0.754308 -3.889153 1.265468
O -0.165266 -3.89278 0.467987
C 0.625106 -4.549814 2.603047
C 1.683801 -4.581657 3.522137
C -0.594917 -5.1594 2.929854
C 1.520349 -5.217867 4.75376
H 2.642386 -4.114629 3.289691
C -0.756287 -5.792738 4.159613
H -1.412863 -5.129804 2.207843
C 0.302768 -5.822829 5.073252
H 2.347413 -5.240849 5.465011
H -1.707651 -6.265618 4.408593
H 0.177451 -6.319915 6.036932
C 3.472529 -4.332035 -1.348585
C 2.585182 -5.403186 -1.181112
C 4.829482 -4.587975 -1.572857
C 3.052425 -6.716811 -1.237319
H 1.521698 -5.205944 -1.023334
C -6.873534 -1.59911 -0.340946
H -7.080753 -0.928504 0.506792
H -7.235865 -1.111801 -1.258949
H -7.460808 -2.517944 -0.188784
C -5.178488 -2.969305 -1.572723
H -4.115259 -3.218005 -1.70513
H -5.721207 -3.89854 -1.339072
H -5.553835 -2.582778 -2.532611
R4_IMC2_Ts2.xyz
50
Energy=-3646.034286
C -2.361879 1.583166 0.600277
O -2.67555 2.760354 0.548147
C -1.886306 0.988576 1.892197
C -0.989354 -0.085976 1.928449
C -2.327321 1.572362 3.088507
C -0.536694 -0.568044 3.160629
H -0.647426 -0.545744 0.997795
C -1.887628 1.077366 4.313983
H -3.024009 2.412134 3.04551
C -0.987529 0.00565 4.350698
H 0.168862 -1.400857 3.188338
H -2.243349 1.52589 5.243238
H -0.63853 -0.380663 5.310372
C -2.339851 0.775963 -0.692814
H -3.036647 1.278653 -1.387234
H -1.323616 0.874801 -1.11107
N -2.625826 -0.644738 -0.575009
C -3.78335 -0.97312 0.118512
C -4.062187 -2.459467 0.443072
N -4.487156 0.009914 0.561628
H -5.241166 -0.312539 1.166422
C -2.223898 -1.371045 -1.767496
H -2.657144 -2.378409 -1.79815
H -2.594548 -0.83875 -2.661878
C -0.717071 -1.57136 -1.870946
O 0.006677 -1.388381 -0.908087
C -0.140643 -1.986628 -3.191241
C -0.893607 -2.037953 -4.37079
C 1.212265 -2.34767 -3.223985
C -0.293969 -2.432513 -5.568147
H -1.952744 -1.777988 -4.375271
C 1.812145 -2.742017 -4.41672
H 1.787238 -2.321307 -2.296864
C 1.056644 -2.786919 -5.593486
H -0.886655 -2.467698 -6.483886
H 2.866878 -3.022455 -4.431445
H 1.522182 -3.09826 -6.530723
Br -1.438882 -4.937616 -2.028085
H 0.783351 -1.073831 1.257828
N -1.082578 -0.357706 1.890727
C -1.369157 0.365575 2.928541
N -2.343625 1.297806 2.95263
H -2.406895 1.672811 3.898589
C -1.436298 2.650455 0.932361
C -2.38777 2.269516 -0.163409
C -3.755458 2.569357 -0.122031
C -1.877177 1.541102 -1.244285
C -4.596653 2.14899 -1.153067
H -4.177936 3.136932 0.708172
C -2.715642 1.121008 -2.275625
H -0.812831 1.299571 -1.259462
C -4.078821 1.427888 -2.232294
H -5.660855 2.387447 -1.113721
H -2.308355 0.55078 -3.11238
H -4.739754 1.099474 -3.036686
C -1.968157 3.300576 2.163669
H -1.256339 3.472754 2.965462
H -3.024037 3.406107 2.378414
Br -1.791454 5.441728 1.297433
O -0.241123 2.432926 0.834778
H -0.775857 0.211204 3.857541
R1_Ts5
41
Energy=-3528.114470
C -0.325441 -2.7981 2.260723
O -1.345942 -3.087027 2.858484
C 0.771879 -3.817161 2.104812
C 2.009035 -3.501182 1.525773
C 0.541204 -5.120813 2.566447
C 3.00016 -4.478187 1.412571
H 2.213341 -2.492411 1.163413
C 1.528778 -6.096726 2.448981
H -0.424018 -5.358094 3.01744
C 2.761439 -5.77565 1.871235
H 3.96156 -4.224482 0.962985
H 1.33978 -7.109968 2.807798
H 3.536934 -6.538435 1.778915
C -0.109011 -1.413004 1.653528
H 0.770157 -0.976698 2.171319
H 0.217401 -1.571417 0.611317
N -1.274233 -0.571596 1.658119
C -1.547497 0.10023 2.741779
N -2.602399 0.959165 2.745816
H -2.764423 1.268601 3.704291
C -1.460479 2.543557 1.066508
C -2.344063 2.385701 -0.134941
C -3.690538 2.771563 -0.146441
C -1.792303 1.783507 -1.271536
C -4.470177 2.558819 -1.283841
H -4.141812 3.246977 0.725545
C -2.570171 1.567772 -2.408457
H -0.745444 1.475722 -1.246973
C -3.911519 1.960477 -2.417008
H -5.517969 2.863756 -1.286224

H -5.405723 -0.765084 1.260937
C -3.058148 -2.712555 -2.400507
H -3.946669 -3.156013 -1.960681
H -3.180125 -1.835656 -3.027148
C -1.741148 -3.129336 -1.823744
O -1.675593 -4.069222 -1.049335
C -0.507798 -2.373253 -2.220779
C -0.4856 -1.439916 -3.265396
C 0.65422 -2.593334 -1.469286
C 0.679781 -0.719062 -3.534634
H -1.366032 -1.278138 -3.890029
C 1.814885 -1.868437 -1.732113
H 0.626249 -3.322569 -0.657563
C 1.827282 -0.925358 -2.764913
H 0.69072 0.0049 -4.351199
H 2.710906 -2.035804 -1.131628
H 2.733546 -0.353647 -2.973481
Br -2.969045 -4.162264 -4.149767
R2_Br
R2_I1
21
Energy=-532.940125
C -0.153377 -0.923 -0.363454
O -0.229419 -2.13763 -0.312215
C -0.410827 -0.220993 -1.676382
C -0.368499 1.174719 -1.806525
C -0.70363 -1.004181 -2.801773
C -0.616893 1.773308 -3.043452
H -0.14356 1.807881 -0.946762
C -0.950687 -0.407248 -4.036643
H -0.734772 -2.089831 -2.693681
C -0.907819 0.985097 -4.159321
H -0.582768 2.860224 -3.134968
H -1.177099 -1.026915 -4.90617
H -1.101602 1.455648 -5.125108
C 0.188826 -0.061026 0.836158
H 1.056741 0.571604 0.543375
H -0.639711 0.673904 0.94819
N 0.429669 -0.809209 2.035959
C 0.71926 -0.044933 3.073959
N 0.802225 1.279252 3.099034
H 1.041203 1.577025 4.04453
H 0.906572 -0.611292 4.006499
R2_I3_O_neg
21
Energy=-532.940967
C -0.891915 0.164861 -0.121441
C 0.668119 0.376054 -0.236908
C -0.190744 2.361318 0.133722
N -1.269753 1.637842 -0.292386
C 5.298248 -5.902217 -1.61655
H 5.516726 -3.750503 -1.70735
C 4.410678 -6.968439 -1.450288
H 2.353075 -7.546009 -1.117093
H 6.359797 -6.094141 -1.782273
H 4.77676 -7.995961 -1.488607
R3_IMC1
48
Energy=-3719.795506
C -1.956237 1.307059 -0.137679
O -2.027997 2.522164 -0.236042
C -1.401892 0.693417 1.11915
C -0.794352 -0.570819 1.116235
C -1.447159 1.436314 2.306569
C -0.237744 -1.080282 2.291851
H -0.769839 -1.160875 0.197506
C -0.905222 0.919989 3.482908
H -1.917738 2.42172 2.297398
C -0.294583 -0.338689 3.474638
H 0.241304 -2.061291 2.28401
H -0.953429 1.499381 4.406652
H 0.134769 -0.742865 4.393568
C -2.322209 0.395259 -1.307939
H -2.976996 1.006434 -1.96069
H -1.377843 0.230057 -1.865338
N -2.885118 -0.88611 -0.958621
C -4.030505 -0.80408 -0.301643
N -4.680441 0.315743 -0.00274
H -5.521382 0.085938 0.528818
C -2.815879 -3.715099 -2.047972
H -3.329584 -4.180762 -1.203309
H -3.444943 -2.969636 -2.53731
C -1.450841 -3.207483 -1.626316
O -0.942699 -3.627609 -0.602572
C -0.712264 -2.281422 -2.543925
C -1.276576 -1.787922 -3.728296
C 0.571003 -1.867657 -2.168488
C -0.569665 -0.878341 -4.513819
H -2.277516 -2.090674 -4.040855
C 1.279552 -0.957887 -2.953868
H 1.005151 -2.258921 -1.246038
C 0.708624 -0.461955 -4.128751
H -1.018012 -0.491773 -5.43052
H 2.276795 -0.635518 -2.649485
H 1.257821 0.252703 -4.744589
Br -2.499476 -5.148615 -3.354918
C -4.56576 -2.142544 0.144415
C -5.782819 -2.642028 -0.334889
C -3.808831 -2.923382 1.02783
C -6.222833 -3.914459 0.041882
H -6.378314 -2.041109 -1.027461
C -2.766192 -3.125246 0.937019
H -2.963418 -4.187673 1.149542
H -1.955773 -3.095737 0.195917
H -2.417569 -2.644915 1.866007
C -5.09358 -2.539436 1.582021
H -6.088098 -2.182096 1.270029
H -5.207857 -3.593162 1.876104
H -4.775984 -1.975937 2.472949
C -4.658461 -3.233908 -0.760271
H -3.890438 -3.702663 -1.392434
H -5.284337 -4.055684 -0.377775
H -5.303459 -2.592231 -1.380513
R4_P1.xyz
31
Energy=-614.199115
C 0.139792 0.198119 -0.020766
C 1.888088 1.475765 -0.47954
C 0.800516 2.25467 -0.144007
N -0.277308 1.439476 0.137253
H 2.90608 1.717697 -0.765975
N 1.447425 0.183441 -0.394906
C 0.706917 3.723116 -0.06992
C -0.511769 4.340272 0.252545
C 1.826901 4.535244 -0.317713
C -0.60821 5.731091 0.324568
H -1.388019 3.719442 0.44702
C 1.728471 5.923965 -0.245719
H 2.787008 4.07753 -0.567235
C 0.509904 6.530653 0.075711
H -1.565105 6.192696 0.576506
H 2.610164 6.537415 -0.441437
H 0.434134 7.617875 0.132138
H 2.006707 -0.643893 -0.582062
C -0.658209 -1.070338 0.186219
C -2.112558 -0.718226 0.508659
H -2.186532 -0.113972 1.424525
H -2.581238 -0.153803 -0.310988
H -2.687066 -1.644516 0.66052
C -0.04895 -1.866929 1.354147
H -0.617025 -2.797084 1.509129
H 0.998419 -2.140628 1.152579
H -0.0785 -1.286733 2.288735
C -0.603149 -1.925762 -1.091296
H 0.429279 -2.215746 -1.341409
H -1.183495 -2.849765 -0.946303
H -1.026676 -1.38598 -1.951585
R4_pHCOBr.xyz
17
Energy=-2955.922823
C -3.443975 1.94489 0.000524
O -2.232164 1.90385 0.000318
H -2.131671 1.093397 -3.288177
H -4.525102 1.794094 -3.304471
C -2.043393 3.052595 2.335624
H -1.385963 3.051681 3.201587
H -3.105766 3.191866 2.49321
Br -1.709516 5.283454 1.816103
O -0.27432 2.262375 1.018147
C -0.668873 0.031234 3.985624
H 0.259409 0.602512 3.817561
H -1.176832 0.473401 4.852977
H -0.382358 -0.997322 4.245775
R1_Ts6
39
Energy=-955.706666
C -0.064774 -2.204849 1.930075
O -0.992973 -2.44699 2.677702
C 0.580594 -3.322198 1.152125
C 1.832137 -3.183896 0.535928
C -0.104596 -4.54154 1.052938
C 2.39183 -4.255717 -0.16282
H 2.385937 -2.246063 0.607179
C 0.448178 -5.606767 0.344437
H -1.079917 -4.636988 1.535433
C 1.70018 -5.465275 -0.263069
H 3.370755 -4.144316 -0.632118
H -0.095443 -6.549849 0.264221
H 2.136814 -6.299248 -0.815713
C 0.501969 -0.789186 1.768971
H 1.446088 -0.785928 2.362001
H 0.822189 -0.673313 0.719622
N -0.387126 0.281179 2.097094
C -0.805922 0.481305 3.345589
N -1.846286 1.263052 3.57131
C -2.335495 1.838226 2.331143
H -3.399957 2.115746 2.445871
H -1.792245 2.771957 2.081772
C -2.200419 0.980617 1.064022
O -1.948781 1.520815 -0.020849
C -2.815316 -0.398582 1.046925
C -3.744599 -0.811765 2.006244
C -2.466478 -1.273561 0.008522
C -4.302028 -2.09313 1.938606
H -4.03374 -0.134994 2.813446
C -3.014206 -2.552814 -0.05545
H -1.749723 -0.936325 -0.744649
C -3.935004 -2.967737 0.914487
H -5.029399 -2.408021 2.690218
H -2.726471 -3.231183 -0.862232
H -4.369272 -3.968625 0.86611
C -0.101359 -0.119216 4.545216
H -0.558756 0.248122 5.473089
H -0.154063 -1.216072 4.536063
H 0.963687 0.162632 4.548924
Ts6_R2

H -2.17691 1.889605 0.094396
H 1.186392 -0.349921 0.407322
N 0.944503 1.755104 0.185254
H 1.033646 0.239309 -1.270083
C -1.43057 -0.603456 -1.353583
C -2.068038 -1.831923 -1.160269
C -1.27806 -0.131592 -2.666633
C -2.545176 -2.576553 -2.24516
H -2.183304 -2.184017 -0.133254
C -1.752703 -0.867442 -3.754299
H -0.786975 0.830143 -2.839389
C -2.389356 -2.096797 -3.547209
H -3.041026 -3.534712 -2.072402
H -1.628776 -0.481167 -4.768578
H -2.761127 -2.673569 -4.396541
O -1.295575 -0.332588 1.037802
H -0.307235 3.417752 0.392825
R2_I3_r2
22
Energy=-533.427035
C -0.898285 0.232693 -0.205738
C 0.652933 0.35001 -0.190893
C -0.151063 2.36267 0.151773
N -1.23359 1.648896 -0.304289
H -2.181401 1.952334 -0.099037
H 1.096252 -0.404382 0.474094
N 0.953108 1.716755 0.252655
H 1.06444 0.198311 -1.201187
C -1.450841 -0.586131 -1.360693
C -2.030428 -1.840746 -1.151076
C -1.341093 -0.091489 -2.66703
C -2.497671 -2.59029 -2.235871
H -2.119073 -2.23172 -0.1374
C -1.805715 -0.839814 -3.748172
H -0.889988 0.889399 -2.835615
C -2.387311 -2.093878 -3.535108
H -2.950715 -3.567829 -2.059865
H -1.71569 -0.442365 -4.760793
H -2.752774 -2.679851 -4.380376
O -1.396074 -0.329347 0.994612
H -0.961257 0.110337 1.744639
H -0.25895 3.425073 0.376466
R2_I5
37
Energy=-916.917363
C 2.326597 0.018649 -0.005986
O 3.520816 -0.137731 0.160901
C 1.713553 1.384634 -0.017403
C 0.332202 1.575678 -0.161604
C 2.556376 2.495878 0.128367
C -4.258541 -4.183325 1.42712
H -2.848197 -2.540858 1.383256
C -5.462821 -4.687185 0.924927
H -7.161482 -4.304972 -0.356893
H -3.66309 -4.77981 2.121654
H -5.808871 -5.679041 1.222293
R3_IMC2_TS1
48
Energy=-3719.799389
C -3.434513 0.7536 0.293673
O -4.325472 1.734092 0.348245
C -2.176729 0.995157 1.160453
C -1.272578 -0.034767 1.460963
C -1.89835 2.283459 1.623704
C -0.11498 0.220613 2.198647
H -1.488134 -1.049799 1.113141
C -0.740955 2.54701 2.365418
H -2.617196 3.072574 1.393318
C 0.157013 1.516486 2.653238
H 0.577894 -0.59394 2.424784
H -0.539452 3.560764 2.719668
H 1.06114 1.718241 3.231344
C -3.009861 0.337778 -1.171082
H -3.169174 1.189033 -1.851528
H -1.943162 0.049594 -1.232125
N -3.835324 -0.808079 -1.551494
C -4.37076 -1.239845 -0.456086
N -4.014411 -0.596903 0.700596
H -4.721646 -0.537239 1.431002
C -2.924149 -4.025758 -1.827265
H -3.659647 -4.485494 -1.160971
H -3.41188 -3.429812 -2.603433
C -1.880207 -3.272578 -1.023525
O -1.837254 -3.421708 0.184054
C -0.902458 -2.399842 -1.741445
C -1.091497 -2.012871 -3.075534
C 0.179024 -1.882032 -1.016988
C -0.221843 -1.097532 -3.665919
H -1.932284 -2.393535 -3.657568
C 1.05948 -0.980526 -1.612666
H 0.320469 -2.19153 0.020387
C 0.855346 -0.583711 -2.937297
H -0.382722 -0.785873 -4.699046
H 1.900708 -0.58225 -1.042887
H 1.535616 0.131513 -3.403586
Br -1.989664 -5.492777 -2.724218
C -5.255662 -2.431991 -0.423557
C -5.297351 -3.252996 0.710667
C -5.986245 -2.792095 -1.564414
C -6.046795 -4.431569 0.697163
C -4.276207 0.699 0.000314
C -5.678226 0.73893 0.000374
C -3.613811 -0.53659 1.4e-05
C -6.40431 -0.452332 0.000146
H -6.217575 1.687098 0.000597
C -4.343282 -1.72275 -0.000218
H -2.522666 -0.553929 -3.9e-05
C -5.741309 -1.681543 -0.00015
H -7.494931 -0.420132 0.000194
H -3.82329 -2.681931 -0.000459
H -6.315267 -2.609809 -0.000335
C -4.217507 3.251802 0.000628
H -4.856293 3.316271 -0.888791
H -4.855882 3.316379 0.890335
Br -3.071814 4.806649 0.000244
R4_Ts1.xyz
50
Energy=-3645.977444
C -3.108528 1.487199 0.018134
O -3.14543 2.730257 0.027108
C -2.766818 0.77968 1.321503
C -1.751375 -0.176674 1.389198
C -3.466122 1.112891 2.490361
C -1.456284 -0.819414 2.59877
H -1.180993 -0.417531 0.488899
C -3.1763 0.47869 3.696353
H -4.256984 1.865685 2.434563
C -2.170387 -0.496807 3.752135
H -0.661137 -1.567994 2.637678
H -3.735161 0.738104 4.598013
H -1.945111 -0.998148 4.695457
C -2.797464 0.720016 -1.283795
H -3.2132 1.355249 -2.091764
H -1.702722 0.673814 -1.440673
N -3.3584 -0.617093 -1.286938
C -4.511102 -0.587268 -0.651923
C -5.218785 -1.918781 -0.351276
N -4.959249 0.584108 -0.183563
H -5.682807 0.490144 0.529663
C -1.154375 -2.918499 -0.38188
H -1.12505 -2.960487 0.710457
H -1.966834 -2.265135 -0.739213
C 0.218467 -2.578916 -0.928047
O 1.192161 -2.652906 -0.200379
C 0.339408 -2.196743 -2.367031
C -0.780294 -1.832467 -3.128913
C 1.611069 -2.219891 -2.956803
C -0.61285 -1.447751 -4.460425
H -1.778462 -1.794715 -2.680338
C 1.770442 -1.852411 -4.290385
H 2.471263 -2.519795 -2.355394
C 0.655613 -1.470356 -5.044838
39
Energy=-916.421474
C -0.064774 -2.204849 1.930075
O -0.992973 -2.44699 2.677702
C 0.580594 -3.322198 1.152125
C 1.832137 -3.183896 0.535928
C -0.104596 -4.54154 1.052938
C 2.39183 -4.255717 -0.16282
H 2.385937 -2.246063 0.607179
C 0.448178 -5.606767 0.344437
H -1.079917 -4.636988 1.535433
C 1.70018 -5.465275 -0.263069
H 3.370755 -4.144316 -0.632118
H -0.095443 -6.549849 0.264221
H 2.136814 -6.299248 -0.815713
C 0.501969 -0.789186 1.768971
H 1.446088 -0.785928 2.362001
H 0.822189 -0.673313 0.719622
N -0.387126 0.281179 2.097094
C -0.805922 0.481305 3.345589
N -1.846286 1.263052 3.57131
C -2.335495 1.838226 2.331143
H -3.399957 2.115746 2.445871
H -1.792245 2.771957 2.081772
C -2.200419 0.980617 1.064022
O -1.948781 1.520815 -0.020849
C -2.815316 -0.398582 1.046925
C -3.744599 -0.811765 2.006244
C -2.466478 -1.273561 0.008522
C -4.302028 -2.09313 1.938606
H -4.03374 -0.134994 2.813446
C -3.014206 -2.552814 -0.05545
H -1.749723 -0.936325 -0.744649
C -3.935004 -2.967737 0.914487
H -5.029399 -2.408021 2.690218
H -2.726471 -3.231183 -0.862232
H -4.369272 -3.968625 0.86611
C -0.101359 -0.119216 4.545216
H -0.558756 0.248122 5.473089
H -0.154063 -1.216072 4.536063
H 0.963687 0.162632 4.548924
R3_Ts5
48
Energy=-3719.766106
C -0.539477 -2.779504 2.205679
O -1.616922 -3.113458 2.664581
C 0.532826 -3.810509 1.972756
C 1.821124 -3.467166 1.538776
C 0.223439 -5.157137 2.210819
C 2.784367 -4.459703 1.347666
H 2.08714 -2.42574 1.351006
C 1.183412 -6.147822 2.015194
H -0.781491 -5.416078 2.548887
C 2.467317 -5.799336 1.583468

C -0.198025 2.867017 -0.156641
H -0.341546 0.724723 -0.274161
C 2.025821 3.783466 0.130189
H 3.630332 2.336005 0.239238
C 0.646458 3.969782 -0.011768
H -1.273874 3.011425 -0.266621
H 2.685934 4.644955 0.242727
H 0.229597 4.978628 -0.009154
C 1.401101 -1.185911 -0.2017
H 0.655096 -1.190664 0.607583
H 0.829341 -1.067865 -1.137708
N 2.113447 -2.437245 -0.192354
C 2.807304 -2.857628 -1.281322
N 2.94486 -2.276214 -2.420347
H 2.470725 -1.370445 -2.463784
C 2.149571 -3.184076 1.040142
H 2.942383 -3.945625 0.986153
H 2.409833 -2.519171 1.881637
C 0.81969 -3.874907 1.342909
O -0.119099 -3.776871 0.575219
C 0.712271 -4.670584 2.606722
C 1.785573 -4.791966 3.500567
C -0.501242 -5.314541 2.888424
C 1.642762 -5.54999 4.663997
H 2.739502 -4.301002 3.300381
C -0.641641 -6.070643 4.049472
H -1.329949 -5.215125 2.185178
C 0.431565 -6.188558 4.939136
H 2.480367 -5.641729 5.357063
H -1.587768 -6.570271 4.263769
H 0.322635 -6.780815 5.849546
H 3.29362 -3.828911 -1.127987
R2_IMC1
38
Energy=-3488.855284
C -1.98917 1.309832 -0.118025
O -2.121211 2.520963 -0.204503
C -1.418452 0.708997 1.134062
C -0.666872 -0.471156 1.09073

Continue in the next column…

H -4.713296 -2.987509 1.594537
C -6.73709 -3.966542 -1.575305
H -5.951165 -2.147018 -
2.444784
C -6.76346 -4.792948 -0.446264
H -6.061852 -5.07406 1.579497
H -7.303769 -4.241346 -
2.466954
H -7.345279 -5.716482 -
0.458445
R3_IMC2_TS2
48
Energy=-3719.846191
C -2.179305 1.213371 -0.370405
O -2.178836 2.426088 -0.478832
C -1.814863 0.561125 0.929014
C -1.150731 -0.671063 0.974831
C -2.127155 1.228954 2.120316
C -0.802577 -1.229632 2.206554
H -0.884134 -1.189841 0.052009
C -1.795417 0.661388 3.349257
H -2.64462 2.189489 2.071785
C -1.132312 -0.569776 3.392771
H -0.275115 -2.184528 2.238532
H -2.053183 1.177585 4.275675
H -0.870555 -1.014603 4.354674
C -2.455727 0.349974 -1.596081
H -3.03972 0.965317 -2.302509
H -1.482287 0.137034 -2.066999
N -3.10943 -0.916035 -1.314684
C -4.297156 -0.831931 -0.626881
N -4.727369 0.33 -0.265887
H -5.546285 0.234802 0.336563
C -2.870941 -1.973338 -2.303582
H -3.63421 -2.750228 -2.205799
H -2.950633 -1.561916 -
3.319418
C -1.506235 -2.634573 -2.058663
O -1.390604 -3.439867 -
1.150098

Continue in the next column…

H -1.480609 -1.142622 -
5.047564
H 2.760948 -1.872744 -4.7477
H 0.778758 -1.180543 -6.090091
Br -1.5505 -4.734478 -0.994121
C -4.608709 -2.471515 0.951676
H -5.147725 -3.377628 1.273596
H -3.550073 -2.739639 0.813616
H -4.670067 -1.73116 1.768578
C -6.729476 -1.732051 -0.151581
H -6.964535 -1.130846 0.740493
H -7.19639 -1.246826 -1.022759
H -7.208493 -2.714502 -
0.014707
C -4.991713 -2.921913 -1.487547
H -3.921238 -3.06115 -1.691386
H -5.420551 -3.901123 -
1.219515
H -5.472006 -2.582632 -
2.419311
R4_Ts2.xyz
50
Energy=-3645.966099
C -1.975493 0.989957 -0.270487
O -2.133782 2.124566 -0.689092
C -1.71863 0.760268 1.193247
C -0.813213 -0.214889 1.631413
C -2.379359 1.564823 2.128821
C -0.568133 -0.377037 2.996878
H -0.29766 -0.842208 0.902352
C -2.155132 1.384047 3.494074
H -3.083508 2.322157 1.776625
C -1.24649 0.413569 3.928751
H 0.147791 -1.128737 3.33441
H -2.686248 2.001323 4.220904
H -1.065467 0.274819 4.996454
C -1.933903 -0.204818 -1.222023
H -2.529441 0.101608 -2.105925

Continue in the next column…

H 3.785945 -4.184456 1.013246
H 0.932889 -7.1939 2.199634
H 3.22161 -6.573721 1.43185
C -0.22178 -1.328101 1.862295
H 0.643576 -1.038094 2.491991
H 0.154518 -1.323908 0.823072
N -1.35109 -0.444245 1.958878
C -1.496569 0.301159 3.016329
N -2.496678 1.217039 3.088313
H -2.518355 1.602515 4.032892
C -1.437432 2.621632 1.15924
C -2.202965 2.229162 -0.068094
C -3.580119 2.443973 -0.205062
C -1.507537 1.561665 -1.083307
C -4.247132 2.003018 -1.34881
H -4.144335 2.963355 0.570748
C -2.170194 1.126786 -2.229536
H -0.437875 1.383146 -0.9583
C -3.543926 1.349275 -2.36419
H -5.320109 2.175102 -1.448849
H -1.61873 0.608915 -3.016228
H -4.068903 1.007733 -3.258359
C -2.166652 3.233147 2.304978
H -1.579928 3.460519 3.190464
H -3.245694 3.277408 2.376307
Br -2.011254 5.373109 1.423675
O -0.232495 2.446133 1.234969
C -0.553999 0.225867 4.197448
C -0.508836 -0.904297 5.023868
C 0.287155 1.31206 4.476535
C 0.351388 -0.940847 6.124365
H -1.159772 -1.755278 4.808172
C 1.156314 1.27028 5.569181
H 0.265973 2.185728 3.819583
C 1.187016 0.145278 6.398492
H 0.370023 -1.821897 6.769086
H 1.812514 2.118662 5.77405
H 1.861912 0.115007 7.255965

Final.
